# Factors influencing the establishment of hospital accreditation programs in low- and middle-income countries: a scoping review

**DOI:** 10.1093/heapol/czaf011

**Published:** 2025-02-18

**Authors:** Dilantha Dharmagunawardene, Paula Bowman, Mark Avery, David Greenfield, Reece Hinchcliff

**Affiliations:** Department of Management, Griffith Business School, Griffith University, South Brisbane, QLD 4101, Australia; Ministry of Health, Colombo 01000, Sri Lanka; School of Public Health and Social Work, Faculty of Health, Queensland University of Technology, Brisbane, QLD 4059, Australia; Department of Management, Griffith Business School, Griffith University, South Brisbane, QLD 4101, Australia; School of Population Health, University of New South Wales, Sydney, NSW 2052, Australia; Department of Management, Griffith Business School, Griffith University, South Brisbane, QLD 4101, Australia; School of Public Health and Social Work, Faculty of Health, Queensland University of Technology, Brisbane, QLD 4059, Australia

**Keywords:** hospital accreditation, patient safety, quality in health care, low- and middle-income countries

## Abstract

Hospital accreditation programs are globally recognized as an important tool for enhancing quality and safety in health care; however, many programs in low- and middle-income countries (LMICs) are discontinued shortly after their establishment. This scoping review synthesized published evidence on factors influencing the establishment and sustainability of hospital accreditation programs in LMICs, to provide guidance to health stakeholders involved in these processes. Six databases were searched using the terms “accreditation,” “health,” “hospital,” and the country list of LMICs. Screening was undertaken collaboratively for validation. A framework to guide data extraction was developed by amalgamating eight existing classifications, theories, models, and frameworks concerning policy diffusion and implementation. The framework comprised the following domains: antecedent influences (A), contextual factors (C), establishment factors (E), standards, surveyors, stimulants (incentives), and survey-related factors (S-4S), governance (G), legislation (L), execution (implementation; E), and assessment and monitoring (AM), forming the ACES-GLEAM framework. Thirty-two sources were identified, with an increasing publication trend over time. The included studies reported upon a broad range of patterns, innovations, influencers, enablers, and barriers concerning accreditation program establishment in LMICs. Key questions emerged, including the degree of government involvement, incorporation of international standards versus development of bespoke standards, the use of local versus external surveyors, the use of financial and other incentives to promote engagement, and mandatory versus voluntary approaches of program implementation. Resource constraints were recognized as the most important barriers to sustainable establishment, while the influence of global accreditation and donor agencies were viewed as presenting both positive and negative impacts. Health stakeholders are encouraged to reflect upon and apply the ACES-GLEAM framework, incorporating the guiding principles outlined in this paper, to help establish hospital accreditation programs in LMICs in a way that facilitates sustainability and effectiveness over time.

Key messages

- Ineffective and non-sustainable establishment of accreditation programs in low- and middle-income countries (LMICs) has led to waste of scarce resources and lost opportunity to improve quality and patient safety, which has a high burden in these resource-constrained settings.
- Reviewed literature indicated that there are multiple challenges of accreditation program establishment in LMICs, such as undue international influences, resource constraints, health system issues, contextual mismatch of standards, and lack of appropriate incentives.
- Stakeholders involved in accreditation program establishment in LMICs can utilize the factors (patterns, innovations, influences, enablers, and barriers) identified in this scoping review to establish effective and sustainable accreditation programs.
- A novel, holistic “ACES-GLEAM” framework outlining the multiplicity of factors influencing accreditation program establishment was developed through this study, which can assist accreditation practitioners and researchers to better understand and optimize future establishment processes.

## Introduction

Low quality, unsafe health care is a significant challenge internationally to health systems (World Health Organization, 2021, World Health Organization, 2022, World Health Organization, 2024). These concerns are particularly important to resource-poor health systems in low- and middle-income countries (LMICs). In LMICs, it is estimated that 25% of those who are hospitalized experience harm and 1 in 24 die due to unsafe care (Slawomirski and Klazinga 2022).

Accreditation is a globally recognized mechanism to improve quality and safety (Q&S) in hospitals and other health care settings (Hinchcliff et al. 2012, Araujo et al. 2020, Alhawajreh et al. 2023, Tabrizi et al. 2023, Wartana et al. 2023, Schmaltz et al. 2024). The International Society for Quality in Health (ISQua) defines accreditation as “a self-assessment and external peer review process used by healthcare organisations to accurately assess their level of performance in relation to established standards and to implement ways to continuously improve the healthcare system” (Fortune et al. 2015). The process of accreditation includes periodic assessment of organizational structures, processes, clinical practices, and outcomes through self-assessment, peer surveyor site visits, and analysis of administrative and clinical data and documentation (Ng et al. 2013, Nicklin et al. 2017).

Since 1917, accreditation programs have been implemented in high-income countries (HICs) (World Health Organization 2003, Jovanovic 2005, Mansour et al. 2020) and have expanded to LMICs over the past three decades. This has commonly involved the diffusion of accreditation standards and/or programs from international accreditation agencies and HICs, with or without adaptation to local contexts (World Health Organization 2003, Braithwaite et al. 2012, Greenfield et al. 2021). Accordingly, standards of HICs in relation to infection control, medication management, health records management, procedural safety, and risk management concepts were diffused and transferred to LMICs improving Q&S (Chu and James 2015, Katz et al. 2018). For these initiatives, technical support has been frequently provided by the World Health Organization (WHO); (Marracino 1993, Núñez 2007, Orjuela de Deeb 2007, Mohssine et al. 2015, Mansour et al. 2020), and financial support sourced from international donor agencies (Lane et al. 2014, Babich 2015, Galukande et al. 2016). Accordingly, health care accreditation has been one of the leading policy initiatives implemented by national governments to improve Q&S not only in LMICs but also in HICs (Greenfield et al. 2015).

Various sources of encouragement were endured for hospital accreditation programs to be established in LMICs, including policies of the WHO and ISQua (World Health Organization 2003), and linkage to strategies promoting Universal Health Coverage (UHC); (Mate et al. 2014, Nicklin et al. 2021). These developments have occurred in combination with the reform of social security and insurance systems (Núñez 2007, Ruelas et al. 2007, Johnson et al. 2016, Spieker 2020, Gutiérrez et al. 2024); however, the diffusion of (highly complex) accreditation programs with multiple interrelated components (Fortune et al. 2015, ISQua 2015) into complex adaptive health systems (Plsek and Greenhalgh 2001) poses challenges. This is particularly difficult in LMIC environments with limited health system resources and weak institutional structures (Mansour et al. 2020).

Case studies from Zambia (Bukonda et al. 2002), Liberia (Cleveland et al. 2011), Egypt, Lebanon (Mansour et al. 2021), Lesotho, Swaziland (Babich 2015), and Sri Lanka (Ministry of Health Nutrition and Indigenous Medicine 2015, Karandagoda 2023), and the review of accreditation programs by Mansour et al. (2020), have explored different challenges in establishing accreditation programs in LMICs. Key issues include the importance of accounting for local context and considering resource implications when introducing programs. These findings indicate that evidence-informed guidance is required to better facilitate and enable hospital accreditation program establishment processes to ensure long-term effectiveness and sustainability.

This scoping review (Arksey and O’Malley 2005, Peters et al. 2015) was conducted to map, summarize, and critically appraise published evidence on the establishment of hospital accreditation programs in LMICs to identify opportunities to better promote sustainable programs. A novel classification framework was developed to guide the presentation of key insights. The results can be used by supranational organizations, accreditation agencies, and national ministries of health to formulate appropriate accreditation-related polices and to optimize the utility of accreditation programs for reducing harm from health care.

## Materials and methods

Because the intention was to broadly explore and map existing evidence in this complex, multidimensional domain, a scoping rather than systematic review, was undertaken (Peters et al. 2015, Munn et al. 2018) using the relevant Joanna Briggs Institute (JBI) framework (JBI Reviewer’s Manual; Peters et al. 2020). This scoping review was reported according to the Preferred Reporting Items for Systematic Reviews and Meta-Analysis (PRISMA-ScR) extension (Tricco et al. 2018). The review question was “What are the characteristics and critical factors that are antecedents to, and involved in, healthcare accreditation program establishment, which may have influenced program sustainability?”

According to the population, concept, and context domains of the JBI framework, the population for the scoping review was hospital accreditation programs in LMICs. The concept was the characteristics and key factors that are antecedent to, and involved in, health care accreditation program establishment processes (World Health Organization 2003, Jaafaripooyan et al. 2011, Hinchcliff et al. 2013). The context was the structures and systems of accreditation programs and the broader health system environments that impact and are impacted by the establishment of hospital accreditation programs in LMICs.

### Eligibility criteria

Studies describing characteristics and determinants of hospital accreditation programs from LMICs were included. Studies describing only nonhospital settings, educational settings, laboratory settings, and studies describing non-accreditation, quality-related programs (quality improvement, licencing, quality assurance, etc.) were excluded. Detailed description of inclusion and exclusion criteria are provided in Supplementary File I. Original research with any type of study design were included, and non-original research, such as commentary and opinion pieces, were excluded.

LMICs are defined by the World Bank as “countries with a Gross National Income per capita between US$1086 and US$4255” (The World Bank 2023). As the World Bank’s list of countries is updated annually, and because the included sources focused retrospectively on the historical evolution of accreditation programs, all countries that had been recorded as LMICs since commencement of the World Bank global classification scheme in 1987 were included. For example, Thailand became an upper middle-income country in 2010 and established its’ accreditation program in 2009. Therefore, Thailand was included to capture the historical developments in the establishment, as this information was deemed important.

No time or language restrictions were used. Translations were obtained using Google® translator to gather key information, with validation and refinement by native speakers.

### Information sources

Embase, MEDLINE, APA PsychInfo, CINAHL with Full Text, Web of Science, and JBI EBP (via Ovid) were searched during June 2023. The same search strategy was run again in June 2024 to update the original list of results. This was combined with citation chaining to capture all relevant sources.

### Search strategy

An initial pilot search was conducted on Embase and EBSCOhost and was subsequently expanded to the other databases. A comprehensive search strategy was then formulated, using lessons learned from the initial search, and input from librarians and subject matter experts. To maximize the capture of all relevant sources, the terms “Accreditation,” “Health,” “Hospital,” and the country list of LMICs were employed for the search. Specific details of the databases with search terms are illustrated in Supplementary File II.

### Selection of sources of evidence

**Figure 1.**
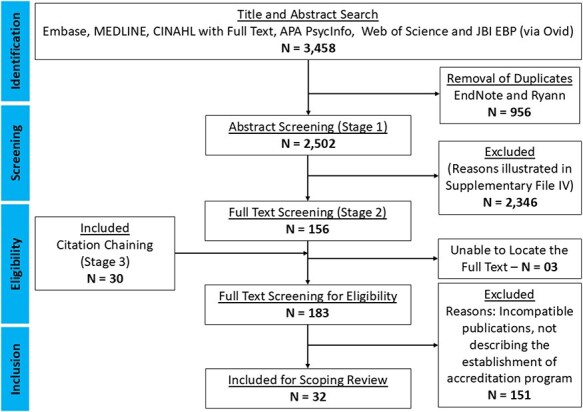
PRISMA-ScR flowchart.

Abstract and full-text screening was performed by the first author and validated by the title and abstract screening of 10% of publications with coauthors. There were no disagreements. In total, 32 publications were selected following full-text screening, which were discussed and confirmed by three authors. The process was recorded and summarized using a PRISMA-ScR flowchart (Fig. 1).

### Data extraction

To guide the categorization of results, the authors first aimed to identify whether theoretical classifications, theories, frameworks, or models had already been used within the accreditation and broader health policy literature to explain the topic of interest. Key concepts emerged from eight classifications, theories, frameworks, models, and two publications from ISQua (Fortune et al. 2015, ISQua 2015), which were amalgamated to provide a new theoretical framework (ACES-GLEAM framework) to guide the analysis of results. The key domains include:

A—Antecedent influences

C—Contextual factors

E—Establishment factors

S-4S—Standards, surveyors, stimulants (incentives), and survey-related factors

G—Governance

L—Legislation

E—Execution (implementation)

AM—Assessment and monitoring

The classifications, theories, frameworks, and models used for the development of the ACES-GLEAM framework are summarized in Table 1.

**Table 1.** Development of the ACES-GLEAM theoretical framework

Classification/theories/framework/model	Description	Example of accreditation or health policy publication
Classification of the Joint Learning Network (JLN) (Helen et al. 2014)	Four main components for developing accreditation programs, i.e. organization or accreditation body, standards with criteria, the process of surveying and surveyors and incentives and institutional support	“JLN for UHC” discussions in 2013 identified four elements of accreditation innovations in LMICs (Helen et al. 2014)
Classification by van Vliet et al. (van Vliet et al. 2023)	Three domains for developing programs, i.e. why (goals), how (implementation), and what (outcomes and lessons learned)	Used to describe implementation of accreditation programs in Australia, Botswana, Denmark, and Jordan (van Vliet et al. 2023)
Donohue and O’Leary’s Framework (Donahue and O’Leary 1997)	Seven elements that can contribute to the success of accreditation programs are mission and philosophy, infrastructure and authority, published performance standards, management of field operations, accreditation decision-making framework, accreditation database, and accreditation program sustainability. The eighth element added by Bukonda et al. (2002) was institutional resources and capacity (Bukonda et al. 2002)	Applied by Bukonda et al. (2002) to describe Zambian accreditation development by incorporating an eighth element (Bukonda et al. 2002)
Model of Successful Organizational Change (Lanteigne 2009)	This model was developed by integrating three previously developed models, i.e. Dimensions of Change (Pomey et al. 2010), Components of Change (Bridges, 2009), and Typology of Change (Denis et al. 2000, Lozeau et al. 2002)	Applied to assess the impact of the programs of Accreditation Canada in achieving organizational change and organizational learning
	Vision (acquisition of new models, comprehension of reflections, new utopia), skill (leadership, skills in quality management, human resources—internal external), incentives (high pressures—internal, external, combined, decentralization of power to the teams, constant evaluation by promoters), resources (excess capacity, legitimate actors, space, discretionary autonomy, cognitive abilities, rational actors), action plan (visibility of commitment of management, dissemination strategies of learning and membership, structure, schedule), possible outcomes (successful organizational change, transformation, strategies, acquisition of quality management, organizational—structure, process, actors, pathways/performance, relationship between the organization and its environment, typology of change, organization transformation)	Used to assess the impact of health facility accreditation in Morocco (Mohssine et al. 2015)
The Responsive Regulation Framework (Ayres and Braithwaite 1992)	This framework was initially developed as a strategy for market governance	Applied to analyze accreditation program establishment in Indonesia and Australia (Hort et al. 2013)
	Healy and Braithwaite, (2006) mentioned that ensuring Q&S should also have the balance of self-regulation through improving organizational and professional culture, and regulated mechanisms for the application of systems and practices (Healy and Braithwaite, 2006)	
	This framework mentioned that good regulatory policy has an inevitable association between state/compulsory regulation and self-regulation. Accordingly, the framework describes relationships between regulatory strategies and processes with efficient, effective, and pragmatic application of regulations (Hort et al. 2013)	
Walt and Gilson Policy Analysis Triangle Framework (Walt and Gilson 1994)	The Walt and Gilson Policy Analysis Triangle Framework has been used for policy analysis and was based on four main domains, i.e. context (systematic factors affecting the policy), content (subjects and topics covered by the policy), process (methods of initiation, formulation, communication, implementation, and evaluation of policies) and actors (individuals, state or member groups and their activities in relation to the policies)	Yousefinezhadi et al. (2017) explored the Iranian hospital accreditation policy-making process (Yousefinezhadi et al. 2017)
	Walt and Gilson (1994) mentioned that despite the simplicity of the model, there are complex inter-relationships between these domains, i.e. context would influence the actors, and actors would influence the processes, resulting in variations in the content of the policy. In addition, the traditional focus on content would diminish the attention on the other three domains, determining the effectiveness of policy options and policy implementation	
Policy Transfer Framework (Dolowitz and Marsh 2000)	This framework by Dolowitz and Marsh is used to analyze the process of transferring policies between settings. Dolowitz and Marsh mentioned that the policy transfer is “a process by which knowledge about policies, administrative arrangements, institutions, and ideas, in one system, is used in the development of policies, administrative arrangements, institutions and ideas in another system.”	Mansour et al. (2021) used the Policy Transfer Framework to describe the establishment of accreditation programs in Egypt, Jordan, and Lebanon (Mansour et al. 2021)
	The Policy Transfer Framework, modified from the previous version in 2000, has six key elements, and as described in the publication, they are as follows; “why do actors engage in policy transfer? Who are the key actors involved in the policy transfer process? What is transferred? From where are lessons drawn? What are the different degrees of transfer? What restricts or facilitates the policy transfer process? and How is the process of policy transfer related to policy “success” or policy ‘failure’?”	This framework is especially useful for explaining accreditation program establishment in LMICs, as most of the accreditation programs in LMICs are drawn from HICs (Yilmaz, 2017; Almutairi and Al Shamsi, 2020; Mansour et al. 2020, Mansour et al. 2021; Alotaibi, 2023)
Diffusion of Innovation (Rogers 1995, Rogers et al. 2014)	Diffusion of innovation framework mentioned five main domains and related subdomains of determining the rate of adopting a new innovation, i.e. perceived attributes of innovation (relative advantage, compatibility, complexity, trialability, observability), type of innovation decision (optional, collective, authority), communication channels (mass media or interpersonal), nature of the social system (social norms, degree of network interconnectedness), and extent of change agents’ promotion efforts	Babich (2015) used the Diffusion of Innovation Framework to explain the influence of global or external actors in establishment of accreditation programs in Lesotho and Swaziland (Babich 2015)
	Determinants of Diffusion, Dissemination, and Implementation of innovations in health service delivery and organization (Greenhalgh et al. 2004, Braithwaite et al. 2018)	
	This framework was expanded to include the determinants of diffusion, dissemination, and implementation of innovations in health service delivery and organization, which additionally included components of factors of outer context, system antecedents, system readiness for innovations, characteristics of adoption/assimilation and implementation process	

### Data charting process

The data extraction process was completed by the first author using a modified template from the JBI Manual, with validation of 10% randomly selected sources by the last author.

### Data items

**Figure 2.**
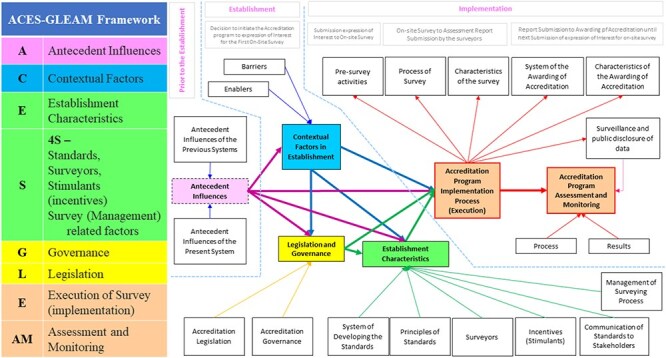
Visualization of the domains of the ACES-GLEAM framework and their relationships.

The following data were gathered: author(s), year of publication, origin/country of origin (where the source study was published or conducted), aims/purpose of the study, methodology/methods, and details of the intervention (accreditation program establishment). Details were gathered in conformance with the ACES-GLEAM framework, as illustrated in Fig. 2. The definitions and descriptions of the identified data extraction items are listed in Supplementary File III.

### Critical appraisal of individual sources of evidence

The Quality Assessment for Diverse Studies tool (Harrison et al. 2021) was used to appraise the methodological and reporting quality of the included publications, and 10% were also completed by coauthors for validation.

### Synthesis of evidence

Descriptive tabular analysis was used to present and analyze data. Basic descriptions of individual studies were analyzed, and then individual studies were mapped against the data items described in the theoretical framework. Finally, detailed summaries of individual studies were thematically synthesized, along with their policy implications.

## Results

The database search yielded 3458 publications. The selected publications were exported initially to EndNote® and then Ryann®. Before the title and abstract screening, 956 duplicates were removed. Following the abstract screening, 156 publications were selected for full-text screening, and a further 30 publications were added through citation chaining. The reasons for exclusions are illustrated in Supplementary File IV. Full-text screening was conducted using a Google® Form, and 151 publications were excluded. The main reasons for exclusions during full-text screening were incompatible studies such as commentaries and opinion pieces (82) and no description of the establishment of accreditation programs (75). Finally, 32 publications were extracted using the search process. The update of the search in June 2024, 1 year after the first search, yielded another 52 initial publications, with 12 duplicates, and no inclusions following full-text review.

## Study characteristics

**Figure 3.**
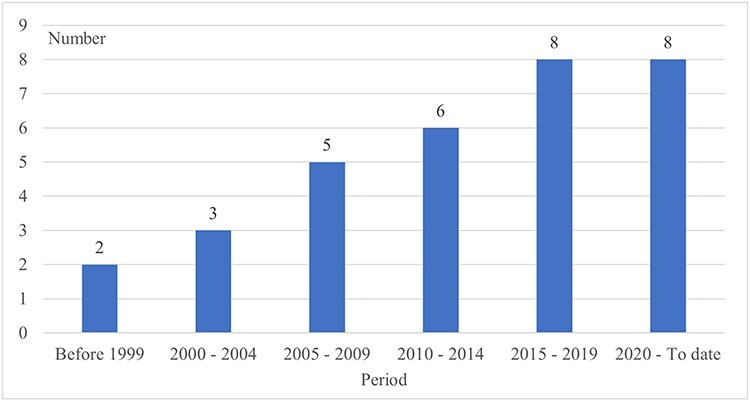
Temporal distribution of publications.

The number of publications gradually increased over time, as illustrated in Fig. 3. Most publications were from the Islamic Republic of Iran (5), followed by Jordan (3), but most countries had only one study completed (Table 2).

**Table 2.** Distribution of publications according to country

Country	Number of publications
Iran, Islamic Republic	5
Jordan	3
India	2
Indonesia	2
Kenya	2
Mexico	2
Lebanon	2
Uganda	2
Argentina	1
Botswana	1
Colombia	1
Costa Rica	1
Egypt	1
Lesotho	1
Liberia	1
Morocco	1
Rwanda	1
South Africa	1
Swaziland	1
Tanzania	1
Thailand	1
Ukraine	1
Zambia	1
Multiple countries (more than 3)—LMICs included	3

## Thematic domains

**Figure 4.**
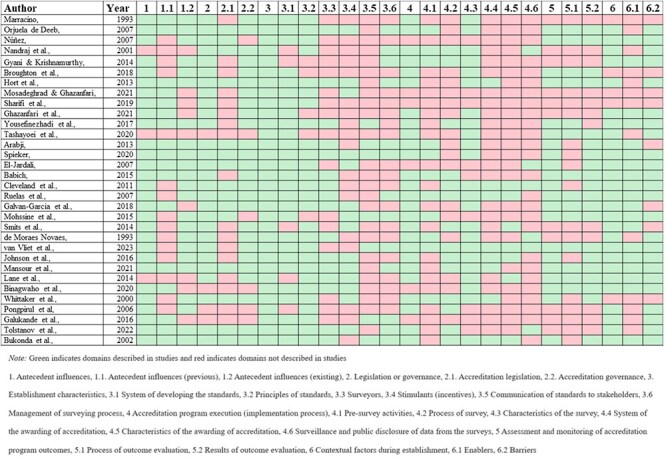
Thematic mapping of individual studies.

The number of publications that reported thematic domains captured in the ACES-GLEAM framework is illustrated in Table 3, with thematic mapping of individual studies depicted in Fig. 4. The most frequently described theme was “Establishment characteristics,” and most frequently described subtheme was “System of developing standards and principles of standards.” Few publications described the subthemes of “Surveillance and public disclosure of data from the surveys,” “Characteristics of the awarding of accreditation,” and “Communication of standards to stakeholders.” “Assessment and monitoring of accreditation programs” was the least described theme. The results are described below according to the thematic domains of the ACES-GLEAM framework. Details of the individual studies are set out in Supplementary File V.

**Table 3.** Distribution of publications according to themes

Thematic area	Number	Percentage
1. Antecedent influences	29	90.6
1.1. Antecedent influences (previous)	17	53.1
1.2 Antecedent influences (existing)	23	71.9
**2. Legislation or governance**	**28**	**87.5**
2.1. Accreditation legislation	15	46.9
2.2. Accreditation governance	26	81.3
**3. Establishment characteristics**	**32**	**100.0**
3.1 System of developing the standards	27	84.4
3.2 Principles of standards	27	84.4
3.3 Surveyors	16	50.0
3.4 Stimulants (incentives)	14	**43.8**
3.5 Communication of standards to stakeholders	7	21.9
3.6 Management of surveying process	13	40.6
**4. Accreditation program execution (implementation process)**	**28**	**87.5**
4.1 Pre-survey activities	11	34.4
4.2 Process of survey	14	43.8
4.3 Characteristics of the survey	23	71.9
4.4 System of the awarding of accreditation	8	25.0
4.5 Characteristics of the awarding of accreditation	7	21.9
4.6 Surveillance and public disclosure of data from the surveys	6	18.8
**5. Assessment and monitoring of accreditation program outcomes**	**20**	**62.5**
5.1 Process of outcome evaluation	13	40.6
5.2 Results of outcome evaluation	20	62.5
**6. Contextual factors during establishment**	**27**	**84.4**
6.1 Enablers	19	59.4
6.2 Barriers	24	75.0

## Theme 1: antecedent influences

The main reason reported for establishing accreditation programs in LMICs is concern over Q&S performance. In addition, the perception of accreditation as a useful tool to help address various challenges of weakened health systems has led to their funding and policy support by international donor agencies (Bukonda et al. 2002, Cleveland et al. 2011, Johnson et al. 2016, Mansour et al. 2021). The objective of achieving UHC has also fuelled the establishment of accreditation programs, as exemplified by the integration of accreditation with the insurance and social security systems (Núñez 2007, Ruelas et al. 2007, Helen et al. 2014, Spieker 2020).

Establishing accreditation programs in LMICs reflects regional influences. Three key accreditation regions were identified from the included literature: the East Mediterranean Regional Organization (EMRO)/WHO model in the Eastern Mediterranean Region (Mohssine et al. 2015, Yousefinezhadi et al. 2017); the African (Southern and Eastern) Region with the influence of Council for Health Services Accreditation of Southern Africa (COHSASA); (Cleveland et al. 2011, Babich 2015, van Vliet et al. 2023) and SafeCare (Johnson et al. 2016, Spieker 2020); as well as the Pan American Health Organization (PAHO) manual in the Latin American and Caribbean Region (de Moraes Novaes 1993, Núñez 2007, Orjuela de Deeb 2007).

## Theme 2: legislation and governance

The literature indicates that LMIC programs were initiated as part of reforms of health systems and implemented through modifications in legislation, regulation, policies, operating and controlling systems, and accountability mechanisms (El-Jardali 2007, Hort et al. 2013, Tolstanov et al. 2022). These reforms are more prominent and wider in scope in sustained programs, such as those in South Africa and Jordan (Whittaker et al. 2000, Mansour et al. 2021). In addition, some modifications were linked with social security legislation, as in Mexico (El-Jardali 2007) and Indonesia (Broughton et al. 2018); however, most of the accreditation programs in LMICs did not report adequate legal enforcement of standards through legislative enactment (Bukonda et al. 2002, Orjuela de Deeb 2007).

The literature identified that most of the accreditation programs in LMICs were either fully or partially government-owned (Malaysia, India, and Thailand) or owned by insurers with support from a ministry of health (Ghana and Kenya) (Helen et al. 2014, Johnson et al. 2016). Some programs were funded solely by governments (India, Iran, and Mexico) (Ruelas et al. 2007, Gyani and Krishnamurthy 2014, Ghazanfari et al. 2021) and others through collaboration between donor agencies and governments (Liberia and Colombia) (Orjuela de Deeb 2007, Cleveland et al. 2011). Few accreditation programs were conducted by private organizations in Mexico and Lebanon, but they were suspended due to variations in assessments, incorrect practices, and poor sustainability (Galvan-Garcia et al. 2018, Mansour et al. 2021).

Many accreditation programs in LMICs were initiated with funding of international donor agencies, including the United States Agency for International Development (Uganda, Zambia, Jordan, Egypt, Lesotho, and Swaziland) (Arabji 2013, Lane et al. 2014, Babich 2015, Galukande et al. 2016). A smaller number of programs were funded solely through insurers (Ghana, Kenya, Nigeria, Namibia, Tanzania, and Zambia through the SafeCare program of PharmAccess®) (Helen et al. 2014, Lane et al. 2014, Johnson et al. 2016, Spieker 2020).

Most of the accreditation programs identified had multi-stakeholder composition for the accreditation committee or the accreditation body, including representation of the public sector, private sector, national-level quality organizations, professional associations (medical, nursing, and allied health), university academics (de Moraes Novaes 1993, Marracino 1993, Whittaker et al. 2000, Bukonda et al. 2002, Hort et al. 2013, Mansour et al. 2021, Tolstanov et al. 2022, van Vliet et al. 2023), and in some cases, also consumer groups, as in Ukraine, South Africa, Argentina, Jordan, and Zambia (Marracino 1993, Whittaker et al. 2000, Bukonda et al. 2002, Mansour et al. 2021, Tolstanov et al. 2022).

## Theme 3: establishment characteristics

### Theme 3.1: system of developing standards

**Figure 5.**
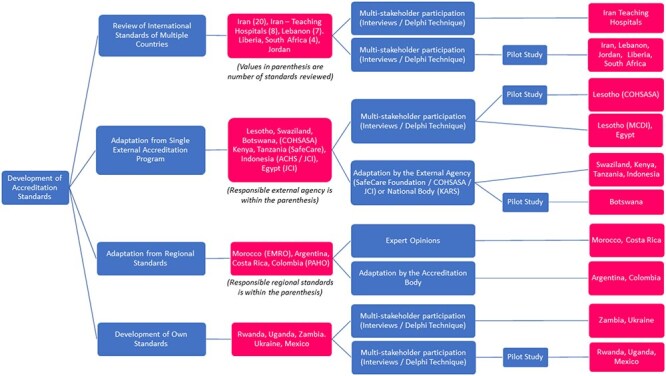
Patterns of accreditation program establishment found in low- and middle-income countries.

Most standards development processes were initiated by adapting international or regional standards (Fig. 5). In some examples, multiple international programs were reviewed and amalgamated (Whittaker et al. 2000, El-Jardali 2007, Cleveland et al. 2011, Sharifi et al. 2019, Mansour et al. 2021, Mosadeghrad and Ghazanfari 2021), while others were adapted from a single international program, such as Joint Commission International (JCI) (Broughton et al. 2018, Mansour et al. 2021), COHSASA (Babich 2015, Mansour et al. 2021), SafeCare (Lane et al. 2014, Johnson et al. 2016, Spieker 2020), or a regional organization (EMRO, PAHO) (Marracino 1993, Núñez 2007, Orjuela de Deeb 2007, Mohssine et al. 2015).

A critical aspect of the establishment process identified in the included studies is the development of country-specific standards, through consultation with multiple stakeholders (Whittaker et al. 2000, Bukonda et al. 2002, El-Jardali 2007, Cleveland et al. 2011, Babich 2015, Galukande et al. 2016, Galvan-Garcia et al. 2018, Sharifi et al. 2019, Binagwaho et al. 2020, Mansour et al. 2021, Mosadeghrad and Ghazanfari 2021, Tolstanov et al. 2022), or the opinion of only a small number of key experts (Núñez 2007, Mohssine et al. 2015). Alternatively, bespoke standards of some accreditation programs were developed by national accreditation bodies (Marracino 1993, Orjuela de Deeb 2007) or through consultation with an external accreditation agency (Lane et al. 2014, Babich 2015, Johnson et al. 2016, Broughton et al. 2018, Spieker 2020, Mansour et al. 2021, van Vliet et al. 2023).

Accordingly, the most common approach in many LMICs has been to develop standards on the basis of a literature review or review of multiple external standards, informed by consultation from local stakeholders for contextualization and, in some cases, pilot testing of the finalized standards. Deviation from this common approach of review and adapting external standards was evidenced in Rwanda (Binagwaho et al. 2020), Uganda (Galukande et al. 2016), Zambia (Bukonda et al. 2002), Ukraine (Tolstanov et al. 2022), and Mexico (Galvan-Garcia et al. 2018). These accreditation programs developed their own accreditation standards with the involvement of national-level experts.

A notable proportion of countries piloted developed standards before wider implementation (Whittaker et al. 2000, El-Jardali 2007, Cleveland et al. 2011, Lane et al. 2014, Babich 2015, Galukande et al. 2016, Yousefinezhadi et al. 2017, Galvan-Garcia et al. 2018, Binagwaho et al. 2020, Mansour et al. 2021, van Vliet et al. 2023).

### Theme 3.2: principles of standards

Based on the published literature, it appears that almost all sets of standards evaluate the structural aspects of care delivery and processes, with less coverage of outcome measures; however, it appears that more mature and evolved programs use standards that are more inclined to measure outcomes, such as those of SafeCare and COHSASA (Johnson et al. 2016), Rwanda (Binagwaho et al. 2020), Lebanon (El-Jardali 2007), and Iran (Yousefinezhadi et al. 2017, Mosadeghrad and Ghazanfari 2021). In contrast, the Mexican accreditation system targeted the assessment of capacity, safety, and quality of health care institutions in ensuring financial accessibility and affordability (Ruelas et al. 2007).

Publications reviewed highlighted that the process of development of standards should be focused on continuous quality improvement and should be developed through systematic, collaborative, and participatory approaches (Orjuela de Deeb 2007, Arabji 2013, Ghazanfari et al. 2021, van Vliet et al. 2023). The need for discussions with more inclusive groups and pre-testing to align with the national context was also highlighted (Babich 2015).

Some programs utilized stepwise or graded implementation of standards. Malaysia and Thailand initiated their accreditation programs with achievable standards and then gradually upgraded the standards with multiple revisions (Helen et al. 2014). The Accreditation Steering Committee in Rwanda similarly designed a three-levelled system (Binagwaho et al. 2020). Additionally, some accreditation programs have had two sets of standards, i.e. ideal or advanced, and basic or minimum levels: Uganda (Galukande et al. 2016), Lebanon (El-Jardali 2007), Accreditation Manual of PAHO (de Moraes Novaes 1993), Argentina (Marracino 1993), and Jordan (Arabji 2013).

### Theme 3.3: surveyors

Most of the surveyors of accreditation programs described in the review were existing health professionals (peer surveyors), as reported for Zambia (Bukonda et al. 2002), Liberia (Cleveland et al. 2011), Mexico (Ruelas et al. 2007), and Uganda (Lane et al. 2014); however, accreditation programs mediated by insurers and related organizations had field officers as surveyors, including in Kenya and Tanzania (Lane et al. 2014). External agencies employed external assessors in Zambia (Bukonda et al. 2002), Lesotho, and Swaziland (Babich 2015). Most of the programs discussed in the literature involved the conduct of surveyor training (Bukonda et al. 2002, Ruelas et al. 2007, Galvan-Garcia et al. 2018, Binagwaho et al. 2020, Ghazanfari et al. 2021), and three surveyor training programs in Jordan, Kenya, and Egypt were ISQua-accredited (Arabji 2013, Johnson et al. 2016, Spieker 2020, Mansour et al. 2021).

### Theme 3.4: stimulants (incentives)

Fast-track payments and greater insurance reimbursement rates for accredited hospitals in collaboration with insurance schemes were the most common mode of monetary incentive identified in the literature: Iran (Mosadeghrad and Ghazanfari 2021), Lebanon (El-Jardali 2007), Mexico (Galvan-Garcia et al. 2018), Kenya, and Tanzania (Lane et al. 2014). In Indonesia, the implementation of National Health Insurance (JKN) had a highly positive impact on accreditation due to its incentive effect (Broughton et al. 2018).

Examples of other monetary incentives identified in the literature were greater funding for accredited Lebanese (Mansour et al. 2021) and Ukrainian (Tolstanov et al. 2022) hospitals, promotion of medical tourism (Galvan-Garcia et al. 2018, Mansour et al. 2021), and making accreditation status mandatory to engage in contractual agreements with the Mexican government (Galvan-Garcia et al. 2018). Two other notable financial incentives were linking accreditation with Performance Based Financing in Rwanda (Binagwaho et al. 2020) and pay-for-performance incentives and discounted rates for the loans and supply contracts in the SafeCare program of Kenya and Tanzania (Lane et al. 2014).

Nonfinancial incentives were also described in the reviewed literature. In South Africa, accreditation provided data for evidence-based policy and planning decisions for government facilities (Whittaker et al. 2000). Moroccan hospital staff perceived that recognition and encouragement by management induced motivation to improve practices and learn (Mohssine et al. 2015). Jordan recognized health care organizations and health staff by awarding appreciation certificates on the National Day of Quality (Mansour et al. 2021). The recognition of Centres of Excellence was introduced in the SafeCare Program in Kenya (Johnson et al. 2016). Finally, branding and promoting public awareness about the accreditation system reportedly motivated the health staff and created healthy competition between facilities in Liberia (Cleveland et al. 2011).

### Theme 3.5: communication of standards

The most common mode of communicating standards in the reviewed publications was the conduct of training programs, as in Iran (Yousefinezhadi et al. 2017), Kenya (Spieker 2020), Morocco (Mohssine et al. 2015), and Botswana (van Vliet et al. 2023). Jordan had a unique participatory approach to communication, using top-down (standards were developed and communicated to the hospitals) and bottom-up approaches (working with hospitals to raise awareness about accreditation standards (Arabji 2013). Additionally, training programs on communication, assessment, scoring, and interpretation of accreditation standards were conducted, and accreditation-related manuals were distributed among users (van Vliet et al. 2023). Similarly, Liberia had early and regular engagement with relevant stakeholders through communication campaigns, meetings, sharing of standards, tools, and schedules, feedback on standards, and press releases, prior to the implementation (Cleveland et al. 2011).

The introduction of the Ugandan and Zambian standards had two unique methods of communication. Initial self-assessment completed by the hospital staff was validated by a visit of experts, combined with training, de-briefing, and clarification of issues in Uganda (Galukande et al. 2016). Zambia conducted educational surveys similar to accreditation surveys without decision on accreditation, develop staff awareness about accreditation, and self-assess their performance against standards (Bukonda et al. 2002).

### Theme 3.6: management of surveying process

The review of publications highlighted that the distribution of templates, gap identification with implementation of quality improvement plans, and establishment of institutional structures were important components of survey management and were responsibilities of the accreditation body (Hort et al. 2013, Binagwaho et al. 2020). In Jordan, the self-assessment templates were provided after training, and mock surveys were conducted for gap identification. Subsequently, health facilities were instructed to employ quality improvement strategies (Plan-Do-Study-Act cycles) to bridge identified gaps (van Vliet et al. 2023). Similarly, health staff in Kenya were trained by the Quality Officers of PharmAccess and Quality Improvement Plans were developed by the hospital quality team with the Quality Officer (participatory approach of the staff to ensure local ownership) (Spieker 2020).

## Theme 4: accreditation program execution (implementation process)

Publications in the scoping review indicated that most accreditation surveys comprised self-assessment followed by periodic, on-site surveys based on standards, every 2 [Jordan (Arabji 2013), Mexico (Ruelas et al. 2007), and Kenya (Spieker 2020)] to 3 years [Colombia (Orjuela de Deeb 2007) and Indonesia (Broughton et al. 2018)]. South African health facilities were reported to undergo an on-site survey and maintain accreditation status at least every 2 years, but accreditation status is awarded for 3 years for consistently well-performing facilities, combined with random inspections (Whittaker et al. 2000). Similarly, Mexican accreditation status was reportedly valid for only 2 years or 3 years for facilities that provide services for six diseases associated with catastrophic health expenditure, combined with follow-up assessments (Ruelas et al. 2007). In India, once accreditation is granted, it is valid for 4 years, but surveillance assessment is conducted within 20–24 months (Gyani and Krishnamurthy 2014).

No consensus was reported in the reviewed publications about voluntary versus mandatory accreditation. Programs described in Liberia (Cleveland et al. 2011), Indonesia (Broughton et al. 2018), and Iran (Tashayoei et al. 2020) were mandatory, but in Colombia (Orjuela de Deeb 2007), Costa Rica (Núñez 2007), and India (Gyani and Krishnamurthy 2014), programs were voluntary.

Some of the programs described in the scoping review employed staged implementation. Argentinian accreditation assessment comprised three levels of increasing demand (Marracino 1993). In India, the same standards were used to grant three categories of accreditation level (Helen et al. 2014), and in Kenya, a stepwise certification process was conducted, complemented by the provision of technical support (Spieker 2020). Similarly, the use of stage-wise implementation as foundation level, basic level, and full accreditation level was viewed as having contributed to the success of implementation in Egypt (Mansour et al. 2021).

The innovative use of information and communication technology (ICT) for program implementation was evident in Liberia (the assessment data were entered using online tools and uploaded to the central server) (Cleveland et al. 2011); the SafeCare system (facilitation of scoring by the on-site use of tablets and laptops) (Johnson et al. 2016); Iran (submission of the assessment scores to the MOH through an online platform) (Yousefinezhadi et al. 2017); and Botswana (the data was uploaded to an online data system) (van Vliet et al. 2023).

### Theme 4.1: pre-survey activities

The pre-survey phase of most accreditation programs mentioned in the scoping review comprised self-assessment, application, and coordination with the accreditation agency. In addition, preparation of a survey schedule (Botswana) (van Vliet et al. 2023) and sending of an agenda, survey plan, and tools to the health facility (Jordan) (van Vliet et al. 2023), along with the distribution of self-assessment documents, were strategies used to facilitate self-assessment.

The SafeCare accreditation program conducted the initial assessment as a situational analysis, identified priorities for improvement, drafted and implemented a quality improvement plan with hospital staff, supported by training and capacity building (Spieker 2020), prior to formal implementation. Similarly, the South African accreditation program implementation was initiated with a baseline self-assessment by hospital staff to identify areas of non-conformance, and then COHSASA provided a blueprint for hospital staff following the analysis of self-assessment report to apply quality improvement methods with a facilitator, assigned by COHSASA, who conducted in-service training for the staff (Whittaker et al. 2000).

### Themes 4.2 and 4.3: process and characteristics of the on-site surveys

The most common survey approach described in the literature was on-site surveys, comprising interviews with staff and consumers to assess the processes and structural aspects of the patient journey and analyze deviations through comparison with a program’s standards (Tolstanov et al. 2022). Most of the described surveys were conducted using document and medical record reviews, site tours, staff interviews, patient interviews, and observations (Bukonda et al. 2002, Cleveland et al. 2011, Yousefinezhadi et al. 2017, van Vliet et al. 2023).

### Themes 4.4 and 4.5: characteristics and system of awarding of accreditation

An entity separate from the accreditation agency usually decided and confirmed the award of accreditation, upon submission of the evaluation report, such as in Mexico (Ruelas et al. 2007) and Liberia (Cleveland et al. 2011). In South Africa, on the recommendation of the technical committee, the COHSASA board decides on the award of accreditation (Whittaker et al. 2000); however, interpreting survey data and making accreditation decisions were responsibilities of Zambia Health Accreditation Council in Zambia (Bukonda et al. 2002).

### Theme 4.6: surveillance and public disclosure of data

Overall, the status of surveillance and use of accreditation data were the least described ACES-GLEAM sub-domain. Some exceptions were in Botswana, where post-survey standards compliance data were regularly reviewed by COHSASA advisers (van Vliet et al. 2023), and Jordanian leaders used accreditation reports for managing health facilities (van Vliet et al. 2023).

Most of the results of accreditation surveys were not disclosed to the public or the status of disclosure was not mentioned in the selected publications. Although the results were not made to the public, some accredited hospitals used accreditation scores for marketing, as was reported in Lebanon (El-Jardali 2007). The PAHO discussions on the development of the accreditation manual mentioned that the final report should be confidential (de Moraes Novaes 1993). Indian stakeholders had divided opinions on disclosure of information, where hospital owners/administrators and professional associations disagreed (Nandraj et al. 2001).

## Theme 5: assessment and monitoring of accreditation programs

Most of the accreditation program assessments described in the included sources were based on qualitative methods, such as interviews, discussions, reviews of documents, and results of on-site surveys (Ruelas et al. 2007, Hort et al. 2013, Babich 2015, Broughton et al. 2018, Mansour et al. 2021, van Vliet et al. 2023), with the exception of Iran (Tashayoei et al. 2020), Mexico (Galvan-Garcia et al. 2018), and Morocco (Mohssine et al. 2015). In Iran, a quantitative technique was employed (Tashayoei et al. 2020), and a “Change Analysis Model” was used in Morocco, adapted from Accreditation Canada (Mohssine et al. 2015). The Mexican assessment used an evaluation card with approved JCI standards (Galvan-Garcia et al. 2018).

Most of the described accreditation programs achieved results at system performance levels such as an increase in the number of accredited hospitals (Nandraj et al. 2001, El-Jardali 2007, Cleveland et al. 2011, Arabji 2013, Lane et al. 2014, Johnson et al. 2016, Binagwaho et al. 2020); expansion of the program (Whittaker et al. 2000, Johnson et al. 2016); increased adherence to standards (Bukonda et al. 2002, Ruelas et al. 2007, Galvan-Garcia et al. 2018, Spieker 2020, van Vliet et al. 2023); increase in the number of surveyors (Arabji 2013, Johnson et al. 2016); transformations in organizational processes such as documentation and record management (Babich 2015, Broughton et al. 2018, Spieker 2020, Mansour et al. 2021); improved training for health staff (Arabji 2013, Johnson et al. 2016); and enhanced positive perceptions of health staff about the benefits of accreditation (Whittaker et al. 2000, Cleveland et al. 2011, Babich 2015, Mohssine et al. 2015, van Vliet et al. 2023).

Significant improvements in performance were reported for all the 10 audited domains in accredited hospitals compared to nonaccredited hospitals (Broughton et al. 2018) and chronological improvements in adherence to standards indicated by improved trends in on-site assessment scores (Lane et al. 2014, Galvan-Garcia et al. 2018, van Vliet et al. 2023) after the implementation of programs Mexico reported a high degree of regional disparities in assessment results (Galvan-Garcia et al. 2018) and the hospital accreditation program in Iran did not achieve the expected results due to constraints in implementation (Yousefinezhadi et al. 2017). Similarly, a previous accreditation program in Indonesia reported a variety of assessment barriers, leading to poor coverage and compliance (Hort et al. 2013).

## Theme 6: contextual factors

### Theme 6.1: contextual factors—enablers

The level of internal and external support was viewed as the most critical element of enabling program establishment. Internal support within the national-level systems included commitment and positive engagement from political entities (Mansour et al. 2021, van Vliet et al. 2023), ministries of health (Binagwaho et al. 2020, Mansour et al. 2021, van Vliet et al. 2023), and accreditation agencies (Bukonda et al. 2002, Galvan-Garcia et al. 2018, Spieker 2020) and were combined with the existence of an appropriate legal framework (Whittaker et al. 2000, Mansour et al. 2021). As activities of accreditation agencies were important enablers (Galvan-Garcia et al. 2018, Spieker 2020), clearly defined entities, responsibilities, and functions for the accreditation agency (Bukonda et al. 2002) were highlighted as being important. Supportive supervision and guidance instead of inspections and enforcement were important enablers in Kenya (Spieker 2020). External organizational support from international entities was in the form of financial support for providing resources and provision of technical support for program establishment and related training (Bukonda et al. 2002, Cleveland et al. 2011, Babich 2015, Mansour et al. 2021, van Vliet et al. 2023).

Process factors in relation to standards development and program implementation were also key enablers. Participatory approaches (Bukonda et al. 2002, van Vliet et al. 2023), adaptation from internationally recognized standards (Spieker 2020, Mansour et al. 2021), and pilot testing (Bukonda et al. 2002, Binagwaho et al. 2020) were the main enablers during standards development. Using stepwise or gradual implementation (Babich 2015, Binagwaho et al. 2020, Spieker 2020, Mansour et al. 2021), educational surveys (Bukonda et al. 2002, Galukande et al. 2016), early and continuous stakeholder engagement (Cleveland et al. 2011), use of online tools (van Vliet et al. 2023), and availability of templates, and guidelines (Cleveland et al. 2011, Spieker 2020, van Vliet et al. 2023) were highlighted as enablers during program implementation.

The review found evidence that surveyors and incentives are important constituents of any accreditation program. Prominent incentive-related enablers were the availability of financial and nonfinancial incentives (Cleveland et al. 2011, Mansour et al. 2021), linkages with insurance systems and insurance reimbursements (Gyani and Krishnamurthy 2014, Lane et al. 2014, Galvan-Garcia et al. 2018, Spieker 2020, Mansour et al. 2021), medical tourism (Gyani and Krishnamurthy 2014, Galvan-Garcia et al. 2018, Mansour et al. 2021), and Performance Based Financing (Binagwaho et al. 2020). The availability of competent surveyors (Bukonda et al. 2002, Galvan-Garcia et al. 2018, Binagwaho et al. 2020), training programs for surveyors (Binagwaho et al. 2020, Mansour et al. 2021), engagement of clinicians (Cleveland et al. 2011), and national-level peer reviewers as surveyors (Ruelas et al. 2007) were emphasized as surveyor-related enablers.

The availability of resources was an important enabler, and these were related to human resources, finance, and infrastructure development (Spieker 2020). One of the main human resource-related enablers was capacity development by implementing training programs (de Moraes Novaes 1993, Bukonda et al. 2002, Arabji 2013, Mansour et al. 2021, van Vliet et al. 2023). Similarly, positive perceptions of health staff toward accreditation was also an important enabler (Arabji 2013, Mohssine et al. 2015), and in Zambia, staff favored accreditation over supervision, as supervision was deemed fault-finding and not facilitative (Bukonda et al. 2002). Health staff in Lesotho and Swaziland considered accreditation a measure of accountability and improving equity of quality care (Babich 2015).

In addition, the accreditation program in Jordan had some characteristic enabling factors at multiple levels (support from international agencies, reformed legislation, support from higher leadership, system-level innovations, institutional-level improvements, and inter-sectoral collaborations with universities and insurance), which contributed to its sustainability and successfulness (Arabji 2013, Mansour et al. 2021, van Vliet et al. 2023).

Additionally, the scoping review highlighted some innovative strategies of implementing accreditation programs in LMICs, such as the use of stepwise implementation, use of ICT, and rapid reporting of results. The use of a stepwise implementation facilitates the continuous improvements amidst the capacity limitations and instils continued motivation for health staff (Helen et al. 2014). Furthermore, multiple levels of implementation with increasing demand facilitated the monitoring, gradual achievement of standards and education of surveyors on how to achieve the next level of demand (Marracino 1993). The use of ICT improved the efficient and accurate data processing and prompt feedback to the health care facility (Johnson et al. 2016). In addition, it improved timely and accurate data communication, facilitation of data analysis, and automated report generation (Cleveland et al. 2011). As delayed reporting was highlighted as one of the failures (Bukonda et al. 2002), Malaysian surveyors conducted on-site night meetings to prepare for the exit conference, which highlighted the areas for improvement and commendations, without revealing the accreditation decision (Helen et al. 2014).

### Theme 6.2: contextual factors—barriers

Resource inadequacy was one of the main reported barriers to accreditation program establishment in LMICs (Bukonda et al. 2002, Hort et al. 2013, Babich 2015, Mohssine et al. 2015, Galukande et al. 2016, Mansour et al. 2020, van Vliet et al. 2023). Financial resources were the critical resource limitation as they contributed to achieving all other resources, such as infrastructure and staffing (Nandraj et al. 2001, Bukonda et al. 2002, Pongpirul et al. 2006, Lane et al. 2014, Babich 2015, Binagwaho et al. 2020, van Vliet et al. 2023).Cessation of donor funding led to the termination of programs in LMICs (Bukonda et al. 2002, Babich 2015, Galukande et al. 2016).

Human resource limitations, such as inadequate human resources (Nandraj et al. 2001, Bukonda et al. 2002, Pongpirul et al. 2006, Núñez 2007, Lane et al. 2014, Babich 2015, Galukande et al. 2016, Yousefinezhadi et al. 2017, Binagwaho et al. 2020, Mansour et al. 2020, Tashayoei et al. 2020), lack of capacity among health staff to establish accreditation programs (Bukonda et al. 2002, Pongpirul et al. 2006, Hort et al. 2013, Gyani and Krishnamurthy 2014, Babich 2015, Mohssine et al. 2015, Galukande et al. 2016, Mansour et al. 2020, Spieker 2020, van Vliet et al. 2023), poor commitment and engagement of staff, especially from leaders and physicians (Pongpirul et al. 2006, Hort et al. 2013, Gyani and Krishnamurthy 2014, Yousefinezhadi et al. 2017), and rapid turnover of human resources (Binagwaho et al. 2020, Mansour et al. 2020, van Vliet et al. 2023), were further barriers identified.

The most notable barrier elicited from the literature, after resource limitations, was the variety of health system issues in relation to program establishment such as leadership changes, less organized structures, poor integration of information, etc. These key system issues were evident in accreditation program establishment in India (Gyani and Krishnamurthy 2014), Indonesia (9), Iran (Yousefinezhadi et al. 2017), Lebanon (El-Jardali 2007), Liberia (Cleveland et al. 2011), Latin American countries (de Moraes Novaes 1993), Zambia (Bukonda et al. 2002), Lesotho, and Swaziland (Babich 2015).

Barriers related to standards, incentives, and staff perceptions were also identified. The main standards-related barriers were high numbers of standards (Yousefinezhadi et al. 2017, Tashayoei et al. 2020); ambiguity (Yousefinezhadi et al. 2017, Tashayoei et al. 2020, Ghazanfari et al. 2021); complexity (Babich 2015, Ghazanfari et al. 2021); compliance gaps (Núñez 2007); more emphasis on documentation (Ruelas et al. 2007, Tashayoei et al. 2020); limited focus on outcomes (Yousefinezhadi et al. 2017); inadequate training (Galukande et al. 2016); and non-alignment with context, budget, incentive systems, and resources (Galukande et al. 2016). These barriers led to limited sustainability of accreditation programs in some LMICs.

Unclear linkages with insurance reimbursements (Lane et al. 2014) and inadequate integration with incentives (Bukonda et al. 2002, Hort et al. 2013, Mohssine et al. 2015, Galukande et al. 2016, Mansour et al. 2021, van Vliet et al. 2023) were the main concerns in relation to incentives. Some perceptual barriers were mentioned, including lack of ownership (Lane et al. 2014, Babich 2015, van Vliet et al. 2023); perception of accreditation merely producing additional work (Spieker 2020), without adding value (Gyani and Krishnamurthy 2014); perceived as time-consuming (Spieker 2020, van Vliet et al. 2023); inherent resistance to change among staff (Spieker 2020, van Vliet et al. 2023); and uncertainty about achieving accreditation status (Babich 2015, van Vliet et al. 2023).

## Discussion

This scoping review identified and synthesized a comprehensive suite of factors involved in the establishment of accreditation programs, which are reported to influence the sustainability. The novel ACES-GLEAM framework was used to categorize these factors, incorporating concepts from eight different classifications, theories, frameworks, and models. The ACES-GLEAM framework includes antecedent, contextual, establishment, governance, legislative, execution, and assessment factors related to accreditation program establishment and represents their interrelationships.

Previous reviews by WHO (World Health Organization 2003) and ISQua (2012 and 2019) (Fortune et al. 2015, ISQua 2015) revealed important characteristics of accreditation programs but did not present in-depth analyses of the influences on and processes involved in program establishment (World Health Organization 2003, Braithwaite et al. 2012, Greenfield et al. 2021). A review by Mansour et al. addressed some of the domains explored in the present study but only searched three databases, included articles published before 2017, and did not include any foreign language publications (Mansour et al. 2020).

The results of this scoping review synthesized themes emerging from the included sources that related to each component of the ACES-GLEAM framework. The discussion will be organized again to cover each of these main themes, to extend the practical and academic value of these results, but through the lens of key issues and challenges for stakeholder consideration. Theoretical literature from the accreditation, Q&S, and health policy literature will be integrated throughout the discussion to unpack key concepts, emphasize key issues for health care stakeholder consideration, and demonstrate how the topic of program establishment in LMICs can best be understood.

A detailed synthesis of policy implications identified within the publications of the scoping review is summarized in Table 4, aligned with the theories, frameworks, and models used to develop the ACES-GLEAM framework.

**Table 4.** Policy implications identified in the scoping review in relation to the theories/models/frameworks used in the development of the ACES-GLEAM theoretical framework

Framework/model	Relevant policy implications identified in the scoping review
Model of Successful Organizational Change (Lanteigne 2009)	The scoping review reported that some of the accreditation programs in LMICs have failed due to rapid turnover of the leadership (Binagwaho et al. 2020, Mansour et al. 2020, van Vliet et al. 2023), and negative influences of external agencies (Bukonda et al. 2002, Babich 2015, Galukande et al. 2016, Mansour et al. 2021) combined with less engagement of front-line and middle-level managers (Pongpirul et al. 2006, Hort et al. 2013, Gyani and Krishnamurthy 2014, Yousefinezhadi et al. 2017) leading to a lack of ownership (Lane et al. 2014, Babich 2015, van Vliet et al. 2023) and resistance to change (Spieker 2020, van Vliet et al. 2023). This emphasizes the requirement of engagement of all strata of institutional structures in establishing accreditation programs in LMICs
	Multiple incentives of accreditation programs were mentioned by the JLN participants (Helen et al. 2014). Availability of monetary incentives and/or linkage with insurance reimbursements was a strong enabling factor (Cleveland et al. 2011, Gyani and Krishnamurthy 2014, Lane et al. 2014, Galvan-Garcia et al. 2018, Binagwaho et al. 2020, Spieker 2020, Mansour et al. 2021)
	Resource-related factors were the most significant contextual barrier identified in the scoping review (Nandraj et al. 2001, Bukonda et al. 2002, Pongpirul et al. 2006, Núñez 2007, Hort et al. 2013, Lane et al. 2014, Babich 2015, Mohssine et al. 2015, Galukande et al. 2016, Yousefinezhadi et al. 2017, Binagwaho et al. 2020, Mansour et al. 2020, Tashayoei et al. 2020, van Vliet et al. 2023)
The Responsive Regulation Framework (Ayres and Braithwaite 1992)	One of the key characteristics identified in the scoping review was the prominent role of policy influencers and top-down approach, such as the involvement of higher levels of organization/system hierarchy (Bukonda et al. 2002, Galvan-Garcia et al. 2018, Binagwaho et al. 2020, Spieker 2020, Mansour et al. 2021, van Vliet et al. 2023), combined with the formulation of legal frameworks (Whittaker et al. 2000, Mansour et al. 2021). This has led to the success of some accreditation programs, such as in Rwanda, South Africa, and Jordan (Whittaker et al. 2000, Binagwaho et al. 2020, Mansour et al. 2021) as accreditation is a complex intervention requiring more technical expertise and involvement of the government, especially in LMICs
	Additionally, the absence of authority associated with government backing or involvement deterred health facilities from applying for accreditation or implementing recommendations of independent accreditation bodies, leading to poor coverage (de Moraes Novaes 1993, Bukonda et al. 2002, Núñez 2007, Hort et al. 2013); however, relying on top-down regulations had drawbacks, especially in LMICs, due to bureaucratization (Nandraj et al. 2001) and political interference (El-Jardali 2007), causing delays, variations, and diversions of accreditation implementation processes (de Moraes Novaes 1993, Bukonda et al. 2002)
	However, relying on a top-down approach without legal backing, with frequent changes in the leadership resulted in the failure of some accreditation programs (Bukonda et al. 2002, Galvan-Garcia et al. 2018, Binagwaho et al. 2020, Mansour et al. 2021, van Vliet et al. 2023). This highlights the importance of balancing self-regulation and compulsory regulation, which may be difficult to achieve within the contextual constraints of LMICs
Walt and Gilson Policy Analysis Triangle Framework (Walt and Gilson 1994)	The evidence from the scoping review indicates that establishing hospital accreditation programs in LMICs, there is generally more focus on, Actors (Bukonda et al. 2002, Galvan-Garcia et al. 2018, Binagwaho et al. 2020, Spieker 2020, Mansour et al. 2021, van Vliet et al. 2023), and Processes (Whittaker et al. 2000, Bukonda et al. 2002, Cleveland et al. 2011, Babich 2015, Galukande et al. 2016, Binagwaho et al. 2020, Spieker 2020, Mansour et al. 2021, van Vliet et al. 2023) **rather than** Content (Núñez 2007, Ruelas et al. 2007, Babich 2015, Galukande et al. 2016, Yousefinezhadi et al. 2017, Tashayoei et al. 2020, Ghazanfari et al. 2021, van Vliet et al. 2023) and Context (Nandraj et al. 2001, Bukonda et al. 2002, Pongpirul et al. 2006, Hort et al. 2013, Lane et al. 2014, Babich 2015, Mohssine et al. 2015, Galukande et al. 2016, Binagwaho et al. 2020, Mansour et al. 2020, van Vliet et al. 2023)
Policy Transfer Framework (Dolowitz and Marsh 2000)	Some of the accreditation programs in LMICs were coercive transfers following the influence of pressure groups and policy influences through direct copying from HICs as a “quick-fix” remedy to existing problems, which created inappropriate transfers due to poor attention being paid to the local contexts of LMICs (Hort et al. 2013, Babich 2015, Broughton et al. 2018, Mansour et al. 2021)
	In contrast, a notable proportion of programs developed their own standards, with the engagement of local experts through emulation of international standards (Whittaker et al. 2000, Bukonda et al. 2002, El-Jardali 2007, Cleveland et al. 2011, Babich 2015, Galukande et al. 2016, Yousefinezhadi et al. 2017, Galvan-Garcia et al. 2018, Sharifi et al. 2019, Binagwaho et al. 2020, Ghazanfari et al. 2021, Mansour et al. 2021, Mosadeghrad and Ghazanfari 2021, Tolstanov et al. 2022); however, some of these programs were not sustained, took comparatively longer time to establish, and needed frequent changes (Bukonda et al. 2002, Galukande et al. 2016, Yousefinezhadi et al. 2017, Galvan-Garcia et al. 2018, Ghazanfari et al. 2021, Mosadeghrad and Ghazanfari 2021), either due to uninformed or incomplete transfers (Dolowitz and Marsh 2000)
	As with many of the domains explored in this study, there does not appear to be a single “best method” for successful, sustainable establishment. Instead, there are key principles that should inform decisions and processes, regardless of the specific approaches taken in each domain
	Notably, some programs were established with the regional influences of PAHO and EMRO through direct copying of regional standards (Dolowitz and Marsh 2000), and these were voluntary transfers with the involvement of more inclusive groups of local experts (Marracino 1993, Núñez 2007, Orjuela de Deeb 2007, Mohssine et al. 2015). Therefore, these transfers of programs were more sustainable and effective, emphasizing the importance of developing programs with a more participatory approach based on regionally guided working principles
Diffusion of Innovation (Rogers 1995, Greenhalgh et al. 2004, Rogers et al. 2014)	There are multiple factors that challenge the establishment and implementation of accreditation programs, especially in LMICs. One group of factors is related to the nature of accreditation as an innovation, such as high complexity, restricted trialability, and limited observability due to multiple interrelated components of accreditation systems
Determinants of Diffusion, Dissemination, and Implementation of innovations in health service delivery and organization (Greenhalgh et al. 2004), (Braithwaite et al. 2018)	Another group is system related, which is less adaptiveness of soft periphery (required structures and systems) with the hardcore (innovation) creating poor “innovation-system fit” (Rogers 1995, Greenhalgh et al. 2004, Rogers et al. 2014)
	Therefore, despite the rapid rate of diffusion (Bukonda et al. 2002, Cleveland et al. 2011, Babich 2015, Mansour et al. 2021, van Vliet et al. 2023) due to compatibility with the perceived needs of policymakers, and due to the relative advantage, the diffusion of accreditation from HICs to LMICs has often been poorly sustained and ineffective due to the above-mentioned challenges. This may have resulted from the lack of system readiness due to inadequate required competencies (Bukonda et al. 2002, Pongpirul et al. 2006, Hort et al. 2013, Babich 2015, Mohssine et al. 2015, Galukande et al. 2016, Mansour et al. 2020, Spieker 2020, van Vliet et al. 2023) and poor adaptiveness of systems and structures (Cleveland et al. 2011, Lane et al. 2014, Babich 2015, Broughton et al. 2018, Spieker 2020, Mansour et al. 2021, van Vliet et al. 2023), leading to inappropriate “innovation-system fit”. This accentuates the need for capacity development of systems and structures of LMICs, combined with improved competencies prior to the establishment of accreditation programs

## Theme 1: antecedent influences

Similar to the scoping review, previous international surveys have reported that improving Q&S, increasing access to government funding, achieving UHC, necessity of compliance with regulations, and promoting medical tourism were the main drivers of global accreditation programs (World Health Organization 2003, Braithwaite et al. 2012, Shaw et al. 2013, Shaw 2015). In addition, international accreditation and donor agencies had major influence in a considerable number of accreditation programs due to the technical and resource limitations in LMICs (Yamey 2012, Mansour et al. 2020, Hinchcliff 2021). This contribution necessitates the developing guiding principles for these stakeholders for their involvement with LMICs, as donor agencies may, in some circumstances, use accreditation programs naively as an overly simplistic remedy to strengthen fundamentally “broken” health systems (Bukonda et al. 2002, Cleveland et al. 2011, Mansour et al. 2021).

## Theme 2: legislation and governance

The recent WHO Global Patient Safety Report (World Health Organization 2024) highlighted that the inadequacy of legislative enactment to support standards implementation in most of the accreditation programs in LMICs, similar to this review. Consequently, lack of legal backing, (Aryankhesal 2016) with frequent changes in the leadership resulted in the failure of some accreditation programs, as the leadership is essential for successful organizational change (Lanteigne 2009) (Table 4) and robust regulations were identified as an important enabler for effective implementation (Hinchcliff et al. 2013).

Braithwaite et al. (2012) and Spieker (2020) indicated the importance of government involvement in the governance of accreditation, with or without the involvement of insurers (Braithwaite et al. 2012, Spieker 2020). The scoping review also highlighted that the support of the government, especially during the initial stages of accreditation program establishment, was essential in LMICs improving coverage, for ensuring credibility, and to benefit from financial subsidies or government reimbursement schemes (Núñez 2007, Cleveland et al. 2011, Galvan-Garcia et al. 2018, Mansour et al. 2021).

However, there were reported drawbacks of the government-managed accreditation programs in LMICs in the scoping review due to the negative aspects of top-down regulation (Ayres and Braithwaite 1992) (Table 4), highlighting the need to ensure a balance between government dependency and independence in governance during the accreditation program establishment in LMICs. This is consistent with the recommendation of Núñez (2007) to ensure the balance of independence between the evaluated (hospitals), regulators (state), and evaluators (accreditation agency and assessors).

## Theme 3: establishment characteristics

### Theme 3.1: system of developing standards

Similar to other published Q&S interventions (Jafari et al. 2018, Marx et al. 2018, García et al. 2019), most reported accreditation programs in LMICs used an approach of adopting international standards to the local context with multi-stakeholder participation; however, some programs intuitively developed their own standards, and the scoping review indicates that there can be drawbacks of this approach, such as poor sustainability (Bukonda et al. 2002, Galukande et al. 2016), and lengthy standards development and finalization processes (Galvan-Garcia et al. 2018).

Additionally, the scoping review highlighted the importance of contextual alignment in the standards development in LMICs to ensure sustainability and avoid repeated unwarranted changes, which was also reflected in other publications (Aryankhesal 2016, Marx et al. 2018, García et al. 2019), and was expected to be achieved through adaptation of external standards with consensus (Becker 1995, Arce 1999, Ammar et al. 2007). In contrast, the scoping review reported that diffusion of standards as coercive transfers due to external influences without local contextual alignment (Dolowitz and Marsh 2000), and inadequate attention to the content and the context (Walt and Gilson 1994) (Table 4) led to the failure of some accreditation programs in LMICs.

A review by Ng et al. (2013) concluded that the development of standards based on legislations, expert opinions, research, international experiences, and existing practices and adaptation of standards in conformance with the local legislations, organizations, expectations, and resources are important considerations (Ng et al. 2013). Therefore, WHO highlights the importance of carefully considering the local adaptation to align with contextual determinants and ensuring the achievement of quality improvement, during the development of standards (World Health Organization 2022).

### Theme 3.2: principles of standards

As valid and relevant standards are important enablers of implementing any accreditation program (Hinchcliff et al. 2013), JLN discussions mentioned that the development of standards should include quality improvement, outcome standards, and use of indicators, in addition to structural and process standards (Helen et al. 2014). Núñez (2007) noted that this may be difficult to achieve in practice as more inclination to process and outcome standards might demotivate health staff when improvements cannot be achieved without resources, but without improvements in processes and outcomes, continuous improvement of quality and care processes cannot be ensured (Núñez 2007). Accordingly, this Costa Rican study (2007) recommended having a balance between structural standards with process and outcome standards, during the development of standards.

### Theme 3.3: surveyors

An analysis of 44 global accreditation programs reported that processes and operational activities of recruitment and development of surveyors were almost similar between HICs and LMICs, but LMICs had more inclination toward certification of new surveyors (Braithwaite et al. 2012), which is a positive factor. Similarly, the scoping review and similar publications have positioned surveyor training as being extremely important because program sustainability was proposed as being strongly influenced by surveyor competencies and reliability (Awadalla and Elhussein 2018, Galvan-Garcia et al. 2018, Mansour et al. 2020, Ghazanfari et al. 2021). The PAHO discussions mentioned that surveyors must have competency and personal qualities of professionalism with a good reputation and experience to make pertinent recommendations for improving the institutional processes and resolving problems during on-site visits and internal discussions. (de Moraes Novaes 1993). Comparably, the scoping review identified that the involvement of clinicians and managers as trained, peer review surveyors in cross-regional assessments, made the process more effective (Cleveland et al. 2011) and ensured regional buy-in for programs (Spieker 2020). Similarly, Sax and Marx (2014) reported that the involvement of local experts as surveyors improved the awareness about accreditation process and tools (Sax and Marx 2014).

Trained surveyors employed from the local health system environment were familiar with the local contexts, ensured minimal costs, and a greater likelihood of sustainability. A high cost/benefit ratio for the Mexican program (Ruelas et al. 2007) and sustainability of SafeCare program (Johnson et al. 2016) were attributed to employing local surveyors. In contrast, external assessors from external agencies were unfamiliar with the local contexts (Babich 2015) and contributed to the high cost in Zambia (Bukonda et al. 2002). However, it appears that employing local and peer surveyors may lead to peer pressure and subjectivity, but external surveyors may be more knowledgeable, objective, and reliable (Ruelas et al. 2007, Ghazanfari et al. 2021). Therefore, employing mixture of local and external surveyors was presumed to be more advantageous during accreditation assessments.

### Theme 3.4: stimulants (incentives)

Incentives were a strong motivation and enabling factor (Hinchcliff et al. 2013) and inadequate incentives were regarded as a barrier (Algunmeeyn et al. 2021). Comparable to the scoping review, the linkages with incentives are common in most of the global accreditation programs (Braithwaite et al. 2012, Ammar et al. 2013, Ng et al. 2013, Shaw 2015, Zaman and Fatima 2015, Mansour et al. 2020). Spieker (2020) highlighted the importance of creating a balance between quality, monetary incentives, and enforcement of regulations to prevent opportunistic behaviour (Spieker 2020), and Ng et al. (2013) concluded that the balance between quality improvement initiatives and incentive-based regulations as important considerations for successful implementation (Ng et al. 2013). Accordingly, systems linked with reimbursements created opportunistic behaviors in Mexico (Gutiérrez et al. 2024) and diverted attention from investing in quality to complex and advanced medical technologies in Lebanon (El-Jardali 2007). In contrast, some studies of the scoping review highlighted the drawbacks of non-availability of monetary incentives, such as staff dissatisfaction (Mohssine et al. 2015) and low motivation of staff (Tolstanov et al. 2022), and not linking with monetary incentives have led to the failure of some accreditation programs (Galukande et al. 2016, Mansour et al. 2021).

## Theme 4: accreditation program execution (implementation process)

A comparison of characteristics between HICs and LMICs by Braithwaite et al. (2012) revealed that there were similarities in processes and operations of accreditation in relation to on-site surveys, evaluation of survey reports, and awarding of accreditation status, but the contrasting feature was more use of mathematical scoring or algorithms for accreditation decisions by the LMICs. Then, while not specifically focused on LMICs, the same study concluded that continued refinement and improvement of program delivery and operations of the accreditation agency were main factors for the sustainability of accreditation programs (Braithwaite et al. 2012). In addition, this scoping review and other reviews highlighted the importance of the supportive and educational role of accreditation (Ammar et al. 2007, Greenfield and Braithwaite 2008, Alkhenizan and Shaw 2011, Lewis and Hinchcliff 2022, Alhawajreh et al. 2023) rather than authoritative inspection processes (Becker 1995, Bateganya et al. 2009, Babich 2015) for improving Q&S.

The scoping review indicated that there were drawbacks of both mandatory and voluntary programs. Similarly, Ensor and Weinzierl (2007) concluded that as a mandatory process, accreditation provides a minimum level of standards for providers to participate in the health care market, but the voluntary basis of accreditation will strive to establish optimum achievable performance (Ensor and Weinzierl 2007). A systematic review of Ng et al. (2013) and this scoping review reported that mandatory programs were more focused on obtaining the compliance certificate rather than improving the quality of care (El-Jardali 2007, Ng et al. 2013, Tolstanov et al. 2022). Additionally, mandatory government programs linked with incentives, without institutional capacity, have led to laissez-faire implementation (Aryankhesal 2016) and even resorting to unwarranted practices such as the use of mobile resources (Gutiérrez et al. 2024). In contrast, few health care organizations were accredited where a voluntary system was in place, resulting in a reduced number of health care institutions that opted for the accreditation (Hort et al. 2013, Gyani and Krishnamurthy 2014).

## Theme 5: contextual factors

Multiple reviews concluded that there were a range of internal and external contextual factors that determined the accreditation program establishment in both HICs and LMICs (Hinchcliff et al. 2013, Ng et al. 2013, Desveaux et al. 2017). Similarly, Braithwaite et al. (2012) reported that the sustainability of any accreditation programs will be impacted by continuous government policy support, stabilized ongoing funding mechanisms, and encouragement of the participation for health care organizations through diversified incentives (Braithwaite et al. 2012). The results of this scoping review verify the global relevance of this principle and identified that the internal support from the national-level systems, external organizational support, incentives, and resources were the most important enablers for the LMICs (Mansour et al. 2020).

The resource-related factors were the most significant barrier, reported in the scoping review and the recent WHO Global Patient Safety Report highlighted that only 11% of global health systems reported to have adequate financial and human resources to implement patient safety-related policies, strategies, action plans, and programs (World Health Organization 2024). Braithwaite et al. (2012) reported that the sustainability of accreditation programs in LMICs is comparatively more inclined toward resource implications and contextual factors, such as behaviors and attitudes of health care workers, than HICs. Accordingly, perceptions, competencies, and engagement of health care workers were thematically classified within contextual factors in this scoping review and were recognized as main influencing factors in many similar studies and reviews (El-Jardali et al. 2012, Hinchcliff et al. 2013, Ng et al. 2013, Awadalla and Elhussein 2018, Mansour et al. 2020, Zapata-Vanegas and Saturno-Hernandez 2020, Algunmeeyn et al. 2021, Hinchcliff 2021).

Overall, this scoping review and the above evidence highlight the importance of providing due attention to the basic resources, especially financial and human resources (including improvement of competencies and perceptions of health workers), to maximize “innovation-system-fit” (Rogers 1995, Greenhalgh et al. 2004, Rogers et al. 2014) (Table 4) for the effective establishment of accreditation programs, particularly in resource-constrained settings of LMICs. These establishment efforts should be supplemented with appropriate external and internal support (Melo 2016; World Health Organization 2022) and innovative strategies, such as stepwise implementation (Ammar et al. 2007, Mansour et al. 2020) and use of digital health (Nasir and Marikar 2018, Hinchcliff 2021, Cayirtepe and Senel 2022), as highlighted in similar studies and reviews.

## Novel ACES-GLEAM framework

The ACES-GLEAM framework was able to classify and describe the characteristics of establishing accreditation programs in LMICs. When compared with the existing frameworks (Policy Transfer Framework, Policy Triangle Framework, and Diffusion of Innovation Model), it is more appropriate and specific to the diffusion or transfer of accreditation programs from external settings to the local settings as a policy intervention (antecedent influences to legislation and governance) (Walt and Gilson 1994, Rogers 1995, Dolowitz and Marsh 2000, Greenhalgh et al. 2004, Rogers et al. 2014, Braithwaite et al. 2018). Although the Responsive Regulatory Framework was relatively more specific to accreditation establishment, it only described balancing self-regulation and compulsory regulation during policy development (Ayres and Braithwaite 1992).

In addition, the ACES-GLEAM framework was able to describe the characteristics of implementing accreditation programs (from establishment to assessment in combination with the contextual factors), from policy-making level to the institution level when compared to other frameworks (Model of Successful Organizational Change, Modified Donohue and O’Leary’s Framework, and Classifications of JLN and van Vliet et al.) (Donahue and O’Leary 1997, Bukonda et al. 2002, Lanteigne 2009, Helen et al. 2014, van Vliet et al. 2023) Although the Model of Successful Organizational Change more comprehensively described the components, other frameworks and classifications were not adequately comprehensive to describe the accreditation program implementation. Overall, all these models and classifications were more specific to implementation aspects and less capable than the ACES-GLEAM framework for describing the continuum of establishment to implementation. In addition, except for JLN classification and Model of Successful Organizational Change, other models and frameworks poorly described the relationship of incentives to the establishment and implementation of accreditation programs.

**Figure 6.**
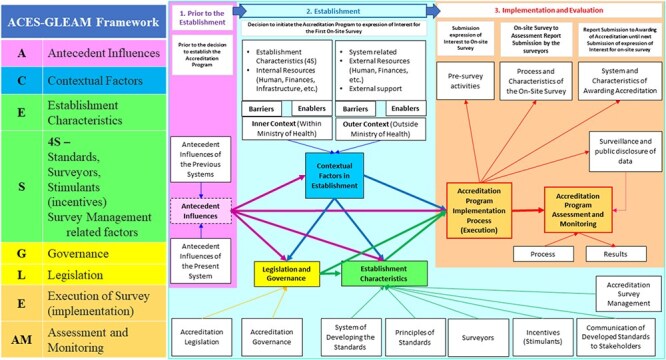
The revised ACES-GLEAM framework and their relationships.

The ACES-GLEAM framework was able to describe the accreditation program establishment trajectory in a simple yet comprehensive manner; however, the contextual factors could be more elaboratively classified than simply as enablers and barriers, as became evident through the results of this scoping review. In the review, the enablers were further classified into internal and external support, process-related factors, resource-related enablers, and incentive-related enablers. Barriers were further classified as resource-related, health system-related, standards-related, incentive-related, and staff perception-related barriers. These additional elements and modifications were incorporated into the revised ACES-GLEAM framework (Fig. 6).

## Limitations

This scoping review employed a broad search strategy to avoid the risk of excluding important literature; however, this created the labor-intensive work of excluding a large volume of publications manually through screening, which introduces the risk of human error. Foreign language publications were translated through Google® translator which may not have the capacity of professional translation. Nevertheless, we were able to capture the essence of foreign language publications adequately.

The ACES-GLEAM framework developed as part of this study has been improved subsequently, especially in relation to contextual factors, but these elements were captured during the thematic analysis of results. Finally, there were subtle differences between identified themes, such as in the management of surveying processes and characteristics of the survey, resulting in some difficulties in explicitly including those themes to a specific category.

## Conclusions

This review indicates that accreditation programs are challenging to establish and implement effectively and sustainably in LMICs. This is due to the inherent complexity of programs, the need for them to integrate synergistically with other regulatory mechanisms, as well as their resource implications. These factors were described and classified according to a novel ACES-GLEAM framework, and common patterns, influences, innovations, enablers, and barriers were elicited. According to the results of this scoping review, the authors postulate that accreditation program establishment, without considering the local context and resource implications, can affect the long-term sustainability of programs and their ability to contribute positively to quality improvement, patient safety, and, therefore, UHC.

These findings suggest that it would be useful to implement accreditation programs in selected health care contexts initially within a broad framework and a set of principles, as each country will have specific contextual characteristics (Mate et al. 2014). The Recommendations of the WHO/ISQua Workshop on Quality Improvement for Middle and Low Income Countries, Dublin, 2000, recommended context-specific or locally developed standards combined with integrated training (World Health Organization 2003) and incorporating innovations such as stepwise implementation (Mansour et al. 2020) and using digital health (Hinchcliff 2021). Devising a standard mechanism, guided by working principles, for the establishment of accreditation programs in LMICs may help to ensure their effectiveness and sustainability. To complete this task successfully, it is important to formulate principles and guidelines that can be adapted to setting-specific characteristics. This study has assisted that process by exploring the existing evidence on prominent factors that influence the way accreditation programs in LMICs have been established and the extent to which they remain implemented sustainably over time.

## Supplementary Material

czaf011_Supp

## Reflexivity statement

The authors include four men and one woman, with expertise in the fields of accreditation practice and research, quality improvement and patient safety, and health policy. The first author is a medical administrator from Sri Lanka, who has held multiple health policy leadership roles and is currently pursuing a PhD. Two of the authors were health care executives who held leadership roles in health care institutions and policy agencies across Australia and the UK. The remaining authors have published extensively in the field of health services accreditation and contribute to accreditation research projects in various countries.
