# Supplementary material for: Factors influencing the establishment of hospital accreditation programs in low- and middle-income countries: a scoping review
Source: Health Policy Plan. 2025 Feb 18;40(4):496–517. doi: 10.1093/heapol/czaf011 (PMC11979593; doi:10.1093/heapol/czaf011)
Supplement: czaf011_Supp [file czaf011_supp.zip › Supp/25-02-15_Scoping-Review-of-Hospital-Accreditation_V16_Supplementary_File_I.docx]

**Supplementary File I: Definitions of Inclusion and Exclusion Criteria**

| **Inclusion Criteria** | **Definition / MESH Term** |
| --- | --- |
| Accreditation | The International Society for Quality in Health (ISQua)  “a self-assessment and external peer review process used by healthcare organizations to accurately assess their level of performance in relation to established standards and to implement ways to continuously improve the healthcare system” (Smits et al., 2014)  MESH - Accreditation (Certification as complying with a standard set by non-governmental organizations, applied for by institutions, programs, and facilities on a voluntary basis.) |
| Hospitals (Secondary and Tertiary Care Centres) | MESH  Health Care Facilities, Manpower, and Services (The services provided in the delivery of health care, associated facilities in health care, and attendant manpower required or available.)  Hospitals  Institutions with an organized medical staff that provide medical care to patients.  Secondary Care Centres  A healthcare facility equipped to provide all but the most specialized forms of care, surgery, and diagnostic techniques.  Secondary Care  Specialized healthcare is delivered as a follow-up or referral from a Primary Care provider.  Tertiary Care Centres  A medical facility that provides a high degree of subspecialty expertise for patients from centres where they received Secondary Care.  Tertiary Healthcare (Care of a highly technical and specialized nature, provided in a medical centre, usually one affiliated with a university, for patients with unusually severe, complex, or uncommon health problems.) |
| Characteristics and Determinants | Factors believed or empirically shown to influence implementation outcomes. Many terms are used for determinants, including barriers, hinders, obstacles, impediments, enablers, and facilitators – Implementation Science *(Nilsen, P., & Bernhardsson, S., 2019)* |
| Lower- and Middle-Income Countries | The World Bank defined lower-middle-income economies as countries those with a Gross National Income per capita between US$ 1,086 and US$ 4,255, in the most recent update of 2023 (The_World_Bank, 2023).  The list of countries is available on the World Bank website (<https://datahelpdesk.worldbank.org/knowledgebase/articles/906519-world-bank-country-and-lending-groups>) |
|  |  |
| **Exclusion Criteria** | **Definition** |
| **Settings and Programs** |  |
| Non-Hospital Settings and Programs | MESH  Organization and Administration  The planning and managing of programs, services, and resources. **(Ministries of Health, Regional Administration Settings)**  Organization and Administration [Subheading]  Used for administrative structure and management  Public Policy  A course or method of action selected, usually by a government, from among alternatives to guide and determine present and future decisions.  Occupational Health  The promotion and maintenance of physical and mental health in the work environment  Education [Subheading]  Used for education, training programs, and courses in various fields and disciplines and for training groups of persons.  Public Health  Branch of medicine concerned with the prevention and control of disease and disability and the promotion of physical and mental health of the population on the international, national, state, or municipal level. |
| Primary Care Settings and Programs | MESH  Primary Health Care  The care which provides integrated, accessible healthcare services by clinicians who are accountable for addressing a large majority of personal healthcare needs, developing a sustained partnership with patients, and practicing in the context of family and community. (JAMA 1995;273(3):192) |
| **Settings** |  |
| Education Settings | MESH  Acquisition of knowledge as a result of instruction in a formal course of study.  Education [Subheading]  Used for education, training programs, and courses in various fields and disciplines and for training groups of persons. |
| Laboratory Settings | MESH  Laboratories  Facilities equipped to carry out investigative procedures.  Clinical Laboratory Services  Organized services provided by MEDICAL LABORATORY PERSONNEL for the purpose of carrying out CLINICAL LABORATORY TECHNIQUES used for the diagnosis, treatment, and prevention of disease. |
| **Programs** |  |
| Licensing | MESH  Licensure  The legal authority or formal permission from authorities to carry on certain activities which by law or regulation require such permission. It may be applied to the licensure of institutions as well as individuals. |
| Credentialing | MESH  Credentialing  The recognition of professional or technical competence through registration, certification, licensure, admission to association membership, the award of a formal and recognized qualification |
| Quality Assurance | MESH  Quality Assurance, Health Care (Activities and programs intended to assure or improve the quality of care in either a defined medical setting or a program. The concept includes the assessment or evaluation of the quality of care, identification of problems or shortcomings in the delivery of care, designing activities to overcome these deficiencies, and follow-up monitoring to ensure the effectiveness of corrective steps.)  Periodic checks to ensure the service is meeting the needs of the customer and population, Actions to address the gaps identified (Shah, 2020)  Assuring quality – External assessment processes and mechanisms to ensure that services are fulfilling stated requirements for quality (external reviews may be referred to as quality assurance) (Introducing the WHO Quality Toolkit: WHO, 2022) |
| Quality Control | MESH  Quality Control  A system for verifying and maintaining a desired level of quality in a product or process by careful planning, use of proper equipment, continued inspection, and corrective action as required. (Random House Unabridged Dictionary, 2d ed)  Identify clear measures of quality for the service and monitor these over time, Take corrective action when appropriate, and internal vigilance to hold gains made through improvement (Shah, 2020)  Assuring quality – Internal assessment processes and mechanisms to ensure that services are fulfilling stated requirements for quality (internal assessment processes may be referred to as quality control) (Introducing the WHO Quality Toolkit: WHO, 2022) |
| Quality Improvement | MESH  Quality Improvement  The attainment or process of attaining a new level of performance or quality.  Identify what matters most, design project and bring together a diverse team, discover solutions through involving those closest to the work, test ideas, implement and scale-up (Shah, 2020) |
