# Supplementary material for: Factors influencing the establishment of hospital accreditation programs in low- and middle-income countries: a scoping review
Source: Health Policy Plan. 2025 Feb 18;40(4):496–517. doi: 10.1093/heapol/czaf011 (PMC11979593; doi:10.1093/heapol/czaf011)
Supplement: czaf011_Supp [file czaf011_supp.zip › Supp/25-02-15_Scoping-Review-of-Hospital-Accreditation_V16_Supplementary_File_II.docx]

**Supplementary File II: Research Databases and Relevant Search Terms**

| **Database** | **Concept** |  | **Search Term** | **Count** |
| --- | --- | --- | --- | --- |
| Embase | Accreditation | 1 | ‘accredit*’:ti | 8,529 |
|  |  | 2 | ‘accredit*’:ab | 34,201 |
|  |  | 3 | 'accreditation'/exp | 69,916 |
|  |  | 4 | 1 or 2 or 3 | 91,199 |
|  | Health | 5 | 'health*':ti | 1,114,299 |
|  |  | 6 | 'health*':ab | 4,276,506 |
|  |  | 7 | 'health'/exp | 878,744 |
|  |  | 8 | 5 or 6 or 7 | 4,956,216 |
|  | Hospital | 9 | 'hospital':ab | 1,816,134 |
|  |  | 10 | 'hospital':ti | 367,025 |
|  |  | 11 | 'hospitals':ab | 383,530 |
|  |  | 12 | 'hospitals':ti | 71,126 |
|  |  | 13 | 'hospital'/exp | 1,435,487 |
|  |  | 14 | 'health care facility'/exp | 2,018,023 |
|  |  | 15 | 9 or 10 or 11 or 12 or 13 or 14 | 3,365,707 |
|  |  | 16 | 4 AND 8 AND 15 | 8,765 |
|  |  | 15 | 'afghanistan*' OR 'albania*' OR 'angola*' OR 'armenia*' OR 'azerbaijan*' OR 'bangladesh*' OR 'belarus*' OR 'belize*' OR 'benin*' OR 'bhutan*' OR 'bolivia*' OR 'bosnia and herzegovina*' OR 'botswana*' OR 'bulgaria*' OR 'burkina faso*' OR 'burundi*' OR 'cabo verde*' OR 'cambodia*' OR 'cameroon*' OR 'central african republic*' OR 'chad*' OR 'chile*' OR 'chin*' OR 'colombia*' OR 'comoros*' OR 'democratic republic of congo*' OR 'congo, republic*' OR 'costa rica*' OR 'côte divoire*' OR 'croatia*' OR 'cuba*' OR 'curaçao*' OR 'czech republic*' OR 'djibouti*' OR 'dominica*' OR 'dominican republic*' OR 'ecuador*' OR 'egypt*' OR 'el salvador*' OR 'equatorial guinea*' OR 'eritrea*' OR 'estonia*' OR 'eswatini*' OR 'ethiopia*' OR 'fiji*' OR 'gambia*, the' OR 'georgia*' OR 'ghana*' OR 'grenada*' OR 'guatemala*' OR 'guinea*' OR 'guinea-bissa*' OR 'guyan*' OR 'haiti*' OR 'honduras*' OR 'india*' OR 'indonesia*' OR 'jamaica*' OR 'jordan*' OR 'kazakhstan*' OR 'kenya*' OR 'kiribati*' OR 'korea*' OR 'korea, democratic republic.' OR 'kosovo*' OR 'kyrgyz*' OR 'lao*' OR 'latvia*' OR 'leban*' OR 'lesotho*' OR 'liberia*' OR 'lithuania*' OR 'madagascar*' OR 'malawi*' OR 'malaysia*' OR 'maldiv*' OR 'mali*' OR 'marshall island*' OR 'mauritania*' OR 'mauriti*' OR 'mexic*' OR 'micronesia*' OR 'micronesia, federal state' OR 'moldov*' OR 'mongolia*' OR 'morocc*' OR 'mozambiq*' OR 'myanmar*' OR 'namibia*' OR 'nepal*' OR 'nicaragua*' OR 'niger*' OR 'nigeria*' OR 'north macedonia*' OR 'mariana island*' OR 'pakistan*' OR 'papua new guinea*' OR 'paraguay*' OR 'peru*' OR 'philippin*' OR 'poland' OR 'polish' OR 'russia*' OR 'rwanda*' OR 'samoa*' OR 'são tomé and príncipe*' OR 'senegal*' OR 'sierra leone*' OR 'slovak* republic' OR 'solomon island*' OR 'somalia*' OR 'south africa*' OR 'south sudan*' OR 'sri lanka*' OR 'st. lucia*' OR 'st. vincent and the grenadi*' OR 'sudan*' OR 'syrian arab republic' OR 'syrian*' OR 'tajikistan*' OR 'tanzania*' OR 'thailand*' OR 'timor-leste*' OR 'togo*' OR 'tonga*' OR 'tunisia*' OR 'türkiye' OR 'turk*' OR 'turkmenistan*' OR 'tuvalu*' OR 'uganda*' OR 'ukrain*' OR 'uzbekistan*' OR 'vanuatu*' OR 'vietnam*' OR 'west bank and gaza*' OR 'yemen*' OR 'zambia*' OR 'zimbabwe*':ti | 12,163,293 |
|  |  | **16** | 'afghanistan*' OR 'albania*' OR 'angola*' OR 'armenia*' OR 'azerbaijan*' OR 'bangladesh*' OR 'belarus*' OR 'belize*' OR 'benin*' OR 'bhutan*' OR 'bolivia*' OR 'bosnia and herzegovina*' OR 'botswana*' OR 'bulgaria*' OR 'burkina faso*' OR 'burundi*' OR 'cabo verde*' OR 'cambodia*' OR 'cameroon*' OR 'central african republic*' OR 'chad*' OR 'chile*' OR 'chin*' OR 'colombia*' OR 'comoros*' OR 'democratic republic of congo*' OR 'congo, republic*' OR 'costa rica*' OR 'côte divoire*' OR 'croatia*' OR 'cuba*' OR 'curaçao*' OR 'czech republic*' OR 'djibouti*' OR 'dominica*' OR 'dominican republic*' OR 'ecuador*' OR 'egypt*' OR 'el salvador*' OR 'equatorial guinea*' OR 'eritrea*' OR 'estonia*' OR 'eswatini*' OR 'ethiopia*' OR 'fiji*' OR 'gambia*, the' OR 'georgia*' OR 'ghana*' OR 'grenada*' OR 'guatemala*' OR 'guinea*' OR 'guinea-bissa*' OR 'guyan*' OR 'haiti*' OR 'honduras*' OR 'india*' OR 'indonesia*' OR 'jamaica*' OR 'jordan*' OR 'kazakhstan*' OR 'kenya*' OR 'kiribati*' OR 'korea*' OR 'korea, democratic republic.' OR 'kosovo*' OR 'kyrgyz*' OR 'lao*' OR 'latvia*' OR 'leban*' OR 'lesotho*' OR 'liberia*' OR 'lithuania*' OR 'madagascar*' OR 'malawi*' OR 'malaysia*' OR 'maldiv*' OR 'mali*' OR 'marshall island*' OR 'mauritania*' OR 'mauriti*' OR 'mexic*' OR 'micronesia*' OR 'micronesia, federal state' OR 'moldov*' OR 'mongolia*' OR 'morocc*' OR 'mozambiq*' OR 'myanmar*' OR 'namibia*' OR 'nepal*' OR 'nicaragua*' OR 'niger*' OR 'nigeria*' OR 'north macedonia*' OR 'mariana island*' OR 'pakistan*' OR 'papua new guinea*' OR 'paraguay*' OR 'peru*' OR 'philippin*' OR 'poland' OR 'polish' OR 'russia*' OR 'rwanda*' OR 'samoa*' OR 'são tomé and príncipe*' OR 'senegal*' OR 'sierra leone*' OR 'slovak* republic' OR 'solomon island*' OR 'somalia*' OR 'south africa*' OR 'south sudan*' OR 'sri lanka*' OR 'st. lucia*' OR 'st. vincent and the grenadi*' OR 'sudan*' OR 'syrian arab republic' OR 'syrian*' OR 'tajikistan*' OR 'tanzania*' OR 'thailand*' OR 'timor-leste*' OR 'togo*' OR 'tonga*' OR 'tunisia*' OR 'türkiye' OR 'turk*' OR 'turkmenistan*' OR 'tuvalu*' OR 'uganda*' OR 'ukrain*' OR 'uzbekistan*' OR 'vanuatu*' OR 'vietnam*' OR 'west bank and gaza*' OR 'yemen*' OR 'zambia*' OR 'zimbabwe*':ab | 12,163,293 |
|  |  | 17 | 'afghanistan' OR 'albania' OR 'angola' OR 'armenia' OR 'azerbaijan' OR 'bangladesh' OR 'belarus' OR 'belize' OR 'benin' OR 'bhutan' OR 'bolivia' OR 'bosnia and herzegovina' OR 'botswana' OR 'bulgaria' OR 'burkina faso' OR 'burundi' OR 'cabo verde' OR 'cambodia' OR 'cameroon' OR 'central african republic' OR 'chad' OR 'chile' OR 'china' OR 'colombia' OR 'comoros' OR 'democratic republic of congo' OR 'congo, republic' OR 'costa rica' OR 'côte divoire' OR 'croatia' OR 'cuba' OR 'curaçao' OR 'czech republic' OR 'djibouti' OR 'dominica' OR 'dominican republic' OR 'ecuador' OR 'egypt' OR 'el salvador' OR 'equatorial guinea' OR 'eritrea' OR 'estonia' OR 'eswatini' OR 'ethiopia' OR 'fiji' OR 'gambia, the' OR 'georgia' OR 'ghana' OR 'grenada' OR 'guatemala' OR 'guinea' OR 'guinea-bissau' OR 'guyana' OR 'haiti' OR 'honduras' OR 'india' OR 'indonesia' OR 'jamaica' OR 'jordan' OR 'kazakhstan' OR 'kenya' OR 'kiribati' OR 'korea, dem. rep.' OR 'kosovo' OR 'kyrgyz republic' OR 'lao pdr' OR 'latvia' OR 'lebanon' OR 'lesotho' OR 'liberia' OR 'lithuania' OR 'madagascar' OR 'malawi' OR 'malaysia' OR 'maldives' OR 'mali' OR 'marshall islands' OR 'mauritania' OR 'mauritius' OR 'mexico' OR 'micronesia, fed. sts.' OR 'moldova' OR 'mongolia' OR 'morocco' OR 'mozambique' OR 'myanmar' OR 'namibia' OR 'nepal' OR 'nicaragua' OR 'niger' OR 'nigeria' OR 'north macedonia' OR 'northern mariana islands' OR 'pakistan' OR 'papua new guinea' OR 'paraguay' OR 'peru' OR 'philippines' OR 'poland' OR 'russian federation' OR 'rwanda' OR 'samoa' OR 'são tomé and príncipe' OR 'senegal' OR 'sierra leone' OR 'slovak republic' OR 'solomon islands' OR 'somalia' OR 'south africa' OR 'south sudan' OR 'sri lanka' OR 'st. lucia' OR 'st. vincent and the grenadines' OR 'sudan' OR 'syrian arab republic' OR 'tajikistan' OR 'tanzania' OR 'thailand' OR 'timor-leste' OR 'togo' OR 'tonga' OR 'tunisia' OR 'türkiye' OR 'turkmenistan' OR 'tuvalu' OR 'uganda' OR 'ukraine' OR 'uzbekistan' OR 'vanuatu' OR 'vietnam' OR 'west bank and gaza' OR 'yemen' OR 'zambia' OR 'zimbabwe':ab | 9,030,603 |
|  |  | **18** | 'afghanistan' OR 'albania' OR 'angola' OR 'armenia' OR 'azerbaijan' OR 'bangladesh' OR 'belarus' OR 'belize' OR 'benin' OR 'bhutan' OR 'bolivia' OR 'bosnia and herzegovina' OR 'botswana' OR 'bulgaria' OR 'burkina faso' OR 'burundi' OR 'cabo verde' OR 'cambodia' OR 'cameroon' OR 'central african republic' OR 'chad' OR 'chile' OR 'china' OR 'colombia' OR 'comoros' OR 'democratic republic of congo' OR 'congo, republic' OR 'costa rica' OR 'côte divoire' OR 'croatia' OR 'cuba' OR 'curaçao' OR 'czech republic' OR 'djibouti' OR 'dominica' OR 'dominican republic' OR 'ecuador' OR 'egypt' OR 'el salvador' OR 'equatorial guinea' OR 'eritrea' OR 'estonia' OR 'eswatini' OR 'ethiopia' OR 'fiji' OR 'gambia, the' OR 'georgia' OR 'ghana' OR 'grenada' OR 'guatemala' OR 'guinea' OR 'guinea-bissau' OR 'guyana' OR 'haiti' OR 'honduras' OR 'india' OR 'indonesia' OR 'jamaica' OR 'jordan' OR 'kazakhstan' OR 'kenya' OR 'kiribati' OR 'korea, dem. rep.' OR 'kosovo' OR 'kyrgyz republic' OR 'lao pdr' OR 'latvia' OR 'lebanon' OR 'lesotho' OR 'liberia' OR 'lithuania' OR 'madagascar' OR 'malawi' OR 'malaysia' OR 'maldives' OR 'mali' OR 'marshall islands' OR 'mauritania' OR 'mauritius' OR 'mexico' OR 'micronesia, fed. sts.' OR 'moldova' OR 'mongolia' OR 'morocco' OR 'mozambique' OR 'myanmar' OR 'namibia' OR 'nepal' OR 'nicaragua' OR 'niger' OR 'nigeria' OR 'north macedonia' OR 'northern mariana islands' OR 'pakistan' OR 'papua new guinea' OR 'paraguay' OR 'peru' OR 'philippines' OR 'poland' OR 'russian federation' OR 'rwanda' OR 'samoa' OR 'são tomé and príncipe' OR 'senegal' OR 'sierra leone' OR 'slovak republic' OR 'solomon islands' OR 'somalia' OR 'south africa' OR 'south sudan' OR 'sri lanka' OR 'st. lucia' OR 'st. vincent and the grenadines' OR 'sudan' OR 'syrian arab republic' OR 'tajikistan' OR 'tanzania' OR 'thailand' OR 'timor-leste' OR 'togo' OR 'tonga' OR 'tunisia' OR 'türkiye' OR 'turkmenistan' OR 'tuvalu' OR 'uganda' OR 'ukraine' OR 'uzbekistan' OR 'vanuatu' OR 'vietnam' OR 'west bank and gaza' OR 'yemen' OR 'zambia' OR 'zimbabwe':ti | 9,030,605 |
|  |  | **19** | 'developing country'/exp | 101,535 |
|  |  | **20** | 'developing countr*' OR 'third world countr*' OR 'low income countr*' OR 'middle-income countr*' OR 'resource constrained setting*' OR 'low- and middle-income countr*' OR 'lmic*' OR 'under-developed countr*' OR 'under developed countr*' OR 'less-developed countr*' OR 'less developed countr*' OR 'least developed countr*':ab | 215,479 |
|  |  | **21** | 'developing countr*' OR 'third world countr*' OR 'low income countr*' OR 'middle-income countr*' OR 'resource constrained setting*' OR 'low- and middle-income countr' OR 'lmic*' OR 'under-developed countr*' OR 'under developed countr*' OR 'less-developed countr*' OR 'less developed countr*' OR 'least developed countr*':ti | 215,312 |
|  |  | **22** | 15 OR 16 OR 17 OR 18 OR 19 OR 20 OR 21 | 12,232,704 |
|  |  | **23** | **4 AND 8 AND 14 AND 22** | **2,159** |

| **Database** | **Concept** |  | **Search Term** | **Count** |
| --- | --- | --- | --- | --- |
| **MEDLINE** | Accreditation | 1 | TI "accredit*" | 6,538 |
|  |  | 2 | AB "accredit*" | 22,959 |
|  |  | 3 | MH accreditation | 15,044 |
|  |  | 4 | 1 or 2 or 3 | 34,218 |
|  | Health | 5 | TI "health*" | 942,301 |
|  |  | 6 | AB "health*" | 3,156,647 |
|  |  | 7 | MH health | 25,045 |
|  |  | 8 | 5 or 6 or 7 | 3,521,163 |
|  | Hospital | 9 | AB "hospital" | 1,090,829 |
|  |  | 10 | TI "hospital" | 261,947 |
|  |  | 11 | AB "hospitals" | 250,174 |
|  |  | 12 | TI "hospitals" | 58,104 |
|  |  | 13 | MH hospitals or hospital | 98,946 |
|  |  | 14 | MH hospitals or health care facilities | 98,946 |
|  |  | 15 | 9 or 10 or 11 or 12 or 13 or 14 | 1,422,174 |
|  |  | 16 | 4 AND 8 AND 15 | 2,705 |
|  |  | 15 | AB “afghanistan*” OR “albania*” OR “angola*” OR “armenia*” OR “azerbaijan*” OR “bangladesh*” OR “belarus*” OR “belize*” OR “benin*” OR “bhutan*” OR “bolivia*” OR “bosnia and herzegovina*” OR “botswana*” OR “bulgaria*” OR “burkina faso*” OR “burundi*” OR “cabo verde*” OR “cambodia*” OR “cameroon*” OR “central african republic*” OR “chad*” OR “chile*” OR “chin*” OR “colombia*” OR “comoros*” OR “democratic republic of congo*” OR “congo, republic*” OR “costa rica*” OR “côte divoire*” OR “croatia*” OR “cuba*” OR “curaçao*” OR “czech republic*” OR “djibouti*” OR “dominica*” OR “dominican republic*” OR “ecuador*” OR “egypt*” OR “el salvador*” OR “equatorial guinea*” OR “eritrea*” OR “estonia*” OR “eswatini*” OR “ethiopia*” OR “fiji*” OR “gambia*, the” OR “georgia*” OR “ghana*” OR “grenada*” OR “guatemala*” OR “guinea*” OR “guinea-bissa*” OR “guyan*” OR “haiti*” OR “honduras*” OR “india*” OR “indonesia*” OR “jamaica*” OR “jordan*” OR “kazakhstan*” OR “kenya*” OR “kiribati*” OR “korea*” OR “korea, democratic republic.” OR “kosovo*” OR “kyrgyz*” OR “lao*” OR “latvia*” OR “leban*” OR “lesotho*” OR “liberia*” OR “lithuania*” OR “madagascar*” OR “malawi*” OR “malaysia*” OR “maldiv*” OR “mali*” OR “marshall island*” OR “mauritania*” OR “mauriti*” OR “mexic*” OR “micronesia*” OR “micronesia, federal state” OR “moldov*” OR “mongolia*” OR “morocc*” OR “mozambiq*” OR “myanmar*” OR “namibia*” OR “nepal*” OR “nicaragua*” OR “niger*” OR “nigeria*” OR “north macedonia*” OR “mariana island*” OR “pakistan*” OR “papua new guinea*” OR “paraguay*” OR “peru*” OR “philippin*” OR “poland” OR “polish” OR “russia*” OR “rwanda*” OR “samoa*” OR “são tomé and príncipe*” OR “senegal*” OR “sierra leone*” OR “slovak* republic” OR “solomon island*” OR “somalia*” OR “south africa*” OR “south sudan*” OR “sri lanka*” OR “st. lucia*” OR “st. vincent and the grenadi*” OR “sudan*” OR “syrian arab republic” OR “syrian*” OR “tajikistan*” OR “tanzania*” OR “thailand*” OR “timor-leste*” OR “togo*” OR “tonga*” OR “tunisia*” OR “türkiye” OR “turk*” OR “turkmenistan*” OR “tuvalu*” OR “uganda*” OR “ukrain*” OR “uzbekistan*” OR “vanuatu*” OR “vietnam*” OR “west bank and gaza*” OR “yemen*” OR “zambia*” OR “zimbabwe*” | 2,103,131 |
|  |  | **16** | TI “afghanistan*” OR “albania*” OR “angola*” OR “armenia*” OR “azerbaijan*” OR “bangladesh*” OR “belarus*” OR “belize*” OR “benin*” OR “bhutan*” OR “bolivia*” OR “bosnia and herzegovina*” OR “botswana*” OR “bulgaria*” OR “burkina faso*” OR “burundi*” OR “cabo verde*” OR “cambodia*” OR “cameroon*” OR “central african republic*” OR “chad*” OR “chile*” OR “chin*” OR “colombia*” OR “comoros*” OR “democratic republic of congo*” OR “congo, republic*” OR “costa rica*” OR “côte divoire*” OR “croatia*” OR “cuba*” OR “curaçao*” OR “czech republic*” OR “djibouti*” OR “dominica*” OR “dominican republic*” OR “ecuador*” OR “egypt*” OR “el salvador*” OR “equatorial guinea*” OR “eritrea*” OR “estonia*” OR “eswatini*” OR “ethiopia*” OR “fiji*” OR “gambia*, the” OR “georgia*” OR “ghana*” OR “grenada*” OR “guatemala*” OR “guinea*” OR “guinea-bissa*” OR “guyan*” OR “haiti*” OR “honduras*” OR “india*” OR “indonesia*” OR “jamaica*” OR “jordan*” OR “kazakhstan*” OR “kenya*” OR “kiribati*” OR “korea*” OR “korea, democratic republic.” OR “kosovo*” OR “kyrgyz*” OR “lao*” OR “latvia*” OR “leban*” OR “lesotho*” OR “liberia*” OR “lithuania*” OR “madagascar*” OR “malawi*” OR “malaysia*” OR “maldiv*” OR “mali*” OR “marshall island*” OR “mauritania*” OR “mauriti*” OR “mexic*” OR “micronesia*” OR “micronesia, federal state” OR “moldov*” OR “mongolia*” OR “morocc*” OR “mozambiq*” OR “myanmar*” OR “namibia*” OR “nepal*” OR “nicaragua*” OR “niger*” OR “nigeria*” OR “north macedonia*” OR “mariana island*” OR “pakistan*” OR “papua new guinea*” OR “paraguay*” OR “peru*” OR “philippin*” OR “poland” OR “polish” OR “russia*” OR “rwanda*” OR “samoa*” OR “são tomé and príncipe*” OR “senegal*” OR “sierra leone*” OR “slovak* republic” OR “solomon island*” OR “somalia*” OR “south africa*” OR “south sudan*” OR “sri lanka*” OR “st. lucia*” OR “st. vincent and the grenadi*” OR “sudan*” OR “syrian arab republic” OR “syrian*” OR “tajikistan*” OR “tanzania*” OR “thailand*” OR “timor-leste*” OR “togo*” OR “tonga*” OR “tunisia*” OR “türkiye” OR “turk*” OR “turkmenistan*” OR “tuvalu*” OR “uganda*” OR “ukrain*” OR “uzbekistan*” OR “vanuatu*” OR “vietnam*” OR “west bank and gaza*” OR “yemen*” OR “zambia*” OR “zimbabwe*” | 1,221,921 |
|  |  | 17 | AB “afghanistan” OR “albania” OR “angola” OR “armenia” OR “azerbaijan” OR “bangladesh” OR “belarus” OR “belize” OR “benin” OR “bhutan” OR “bolivia” OR “bosnia and herzegovina” OR “botswana” OR “bulgaria” OR “burkina faso” OR “burundi” OR “cabo verde” OR “cambodia” OR “cameroon” OR “central african republic” OR “chad” OR “chile” OR “china” OR “colombia” OR “comoros” OR “democratic republic of congo” OR “congo, republic” OR “costa rica” OR “côte divoire” OR “croatia” OR “cuba” OR “curaçao” OR “czech republic” OR “djibouti” OR “dominica” OR “dominican republic” OR “ecuador” OR “egypt” OR “el salvador” OR “equatorial guinea” OR “eritrea” OR “estonia” OR “eswatini” OR “ethiopia” OR “fiji” OR “gambia, the” OR “georgia” OR “ghana” OR “grenada” OR “guatemala” OR “guinea” OR “guinea-bissau” OR “guyana” OR “haiti” OR “honduras” OR “india” OR “indonesia” OR “jamaica” OR “jordan” OR “kazakhstan” OR “kenya” OR “kiribati” OR “korea, dem. rep.” OR “kosovo” OR “kyrgyz republic” OR “lao pdr” OR “latvia” OR “lebanon” OR “lesotho” OR “liberia” OR “lithuania” OR “madagascar” OR “malawi” OR “malaysia” OR “maldives” OR “mali” OR “marshall islands” OR “mauritania” OR “mauritius” OR “mexico” OR “micronesia, fed. sts.” OR “moldova” OR “mongolia” OR “morocco” OR “mozambique” OR “myanmar” OR “namibia” OR “nepal” OR “nicaragua” OR “niger” OR “nigeria” OR “north macedonia” OR “northern mariana islands” OR “pakistan” OR “papua new guinea” OR “paraguay” OR “peru” OR “philippines” OR “poland” OR “russian federation” OR “rwanda” OR “samoa” OR “são tomé and príncipe” OR “senegal” OR “sierra leone” OR “slovak republic” OR “solomon islands” OR “somalia” OR “south africa” OR “south sudan” OR “sri lanka” OR “st. lucia” OR “st. vincent and the grenadines” OR “sudan” OR “syrian arab republic” OR “tajikistan” OR “tanzania” OR “thailand” OR “timor-leste” OR “togo” OR “tonga” OR “tunisia” OR “türkiye” OR “turkmenistan” OR “tuvalu” OR “uganda” OR “ukraine” OR “uzbekistan” OR “vanuatu” OR “vietnam” OR “west bank and gaza” OR “yemen” OR “zambia” OR “zimbabwe”: | 944,105 |
|  |  | **18** | TI “afghanistan” OR “albania” OR “angola” OR “armenia” OR “azerbaijan” OR “bangladesh” OR “belarus” OR “belize” OR “benin” OR “bhutan” OR “bolivia” OR “bosnia and herzegovina” OR “botswana” OR “bulgaria” OR “burkina faso” OR “burundi” OR “cabo verde” OR “cambodia” OR “cameroon” OR “central african republic” OR “chad” OR “chile” OR “china” OR “colombia” OR “comoros” OR “democratic republic of congo” OR “congo, republic” OR “costa rica” OR “côte divoire” OR “croatia” OR “cuba” OR “curaçao” OR “czech republic” OR “djibouti” OR “dominica” OR “dominican republic” OR “ecuador” OR “egypt” OR “el salvador” OR “equatorial guinea” OR “eritrea” OR “estonia” OR “eswatini” OR “ethiopia” OR “fiji” OR “gambia, the” OR “georgia” OR “ghana” OR “grenada” OR “guatemala” OR “guinea” OR “guinea-bissau” OR “guyana” OR “haiti” OR “honduras” OR “india” OR “indonesia” OR “jamaica” OR “jordan” OR “kazakhstan” OR “kenya” OR “kiribati” OR “korea, dem. rep.” OR “kosovo” OR “kyrgyz republic” OR “lao pdr” OR “latvia” OR “lebanon” OR “lesotho” OR “liberia” OR “lithuania” OR “madagascar” OR “malawi” OR “malaysia” OR “maldives” OR “mali” OR “marshall islands” OR “mauritania” OR “mauritius” OR “mexico” OR “micronesia, fed. sts.” OR “moldova” OR “mongolia” OR “morocco” OR “mozambique” OR “myanmar” OR “namibia” OR “nepal” OR “nicaragua” OR “niger” OR “nigeria” OR “north macedonia” OR “northern mariana islands” OR “pakistan” OR “papua new guinea” OR “paraguay” OR “peru” OR “philippines” OR “poland” OR “russian federation” OR “rwanda” OR “samoa” OR “são tomé and príncipe” OR “senegal” OR “sierra leone” OR “slovak republic” OR “solomon islands” OR “somalia” OR “south africa” OR “south sudan” OR “sri lanka” OR “st. lucia” OR “st. vincent and the grenadines” OR “sudan” OR “syrian arab republic” OR “tajikistan” OR “tanzania” OR “thailand” OR “timor-leste” OR “togo” OR “tonga” OR “tunisia” OR “türkiye” OR “turkmenistan” OR “tuvalu” OR “uganda” OR “ukraine” OR “uzbekistan” OR “vanuatu” OR “vietnam” OR “west bank and gaza” OR “yemen” OR “zambia” OR “zimbabwe”: | 635,688 |
|  |  | **19** | MH developing countries or developing nations or third world or low income countries | 80,712 |
|  |  | **20** | TI ("developing countr*" or "third world countr*" or "low income countr*" or "middle-income countr*" or "resource constrained setting*" or "Low- and middle-income countr*" or "LMIC*" or “Under-Developed Countr*” or “Under Developed Countr*” or “Less-Developed Countr*” or “Less Developed Countr*” or "Least Developed Countr*") | 22,877 |
|  |  | **21** | AB ("developing countr*" or "third world countr*" or "low income countr*" or "middle-income countr*" or "resource constrained setting*" or "Low- and middle-income countr*" or "LMIC*" or “Under-Developed Countr*” or “Under Developed Countr*” or “Less-Developed Countr*” or “Less Developed Countr*” or "Least Developed Countr*") | 100,793 |
|  |  | **22** | 15 OR 16 OR 17 OR 18 OR 20 OR 21 OR 22 | 2,570,086 |
|  |  | **23** | **4 AND 8 AND 14 AND 22** | **400** |

| **Database** | **Concept** |  | **Search Term** | **Count** |
| --- | --- | --- | --- | --- |
| CINAHL with Full Text | Accreditation | 1 | TI "accredit*" | 4,869 |
|  |  | 2 | AB "accredit*" | 12,459 |
|  |  | 3 | MH accreditation | 11,658 |
|  |  | 4 | 1 or 2 or 3 | 21,884 |
|  | Health | 5 | TI "health*" | 557,317 |
|  |  | 6 | AB "health*" | 1,231,273 |
|  |  | 7 | MH health | 20,452 |
|  |  | 8 | 5 or 6 or 7 | 1,500,183 |
|  | Hospital | 9 | AB "hospital" | 353,580 |
|  |  | 10 | TI "hospital" | 103,931 |
|  |  | 11 | AB "hospitals" | 105,250 |
|  |  | 12 | TI "hospitals" | 25,717 |
|  |  | 13 | MH hospitals or hospital | 64,627 |
|  |  | 14 | MH hospitals or health care facilities | 64,627 |
|  |  | 15 | 9 or 10 or 11 or 12 or 13 or 14 | 504,770 |
|  |  | 16 | 4 AND 8 AND 15 | 1,444 |
|  |  | 15 | AB “afghanistan*” OR “albania*” OR “angola*” OR “armenia*” OR “azerbaijan*” OR “bangladesh*” OR “belarus*” OR “belize*” OR “benin*” OR “bhutan*” OR “bolivia*” OR “bosnia and herzegovina*” OR “botswana*” OR “bulgaria*” OR “burkina faso*” OR “burundi*” OR “cabo verde*” OR “cambodia*” OR “cameroon*” OR “central african republic*” OR “chad*” OR “chile*” OR “chin*” OR “colombia*” OR “comoros*” OR “democratic republic of congo*” OR “congo, republic*” OR “costa rica*” OR “côte divoire*” OR “croatia*” OR “cuba*” OR “curaçao*” OR “czech republic*” OR “djibouti*” OR “dominica*” OR “dominican republic*” OR “ecuador*” OR “egypt*” OR “el salvador*” OR “equatorial guinea*” OR “eritrea*” OR “estonia*” OR “eswatini*” OR “ethiopia*” OR “fiji*” OR “gambia*, the” OR “georgia*” OR “ghana*” OR “grenada*” OR “guatemala*” OR “guinea*” OR “guinea-bissa*” OR “guyan*” OR “haiti*” OR “honduras*” OR “india*” OR “indonesia*” OR “jamaica*” OR “jordan*” OR “kazakhstan*” OR “kenya*” OR “kiribati*” OR “korea*” OR “korea, democratic republic.” OR “kosovo*” OR “kyrgyz*” OR “lao*” OR “latvia*” OR “leban*” OR “lesotho*” OR “liberia*” OR “lithuania*” OR “madagascar*” OR “malawi*” OR “malaysia*” OR “maldiv*” OR “mali*” OR “marshall island*” OR “mauritania*” OR “mauriti*” OR “mexic*” OR “micronesia*” OR “micronesia, federal state” OR “moldov*” OR “mongolia*” OR “morocc*” OR “mozambiq*” OR “myanmar*” OR “namibia*” OR “nepal*” OR “nicaragua*” OR “niger*” OR “nigeria*” OR “north macedonia*” OR “mariana island*” OR “pakistan*” OR “papua new guinea*” OR “paraguay*” OR “peru*” OR “philippin*” OR “poland” OR “polish” OR “russia*” OR “rwanda*” OR “samoa*” OR “são tomé and príncipe*” OR “senegal*” OR “sierra leone*” OR “slovak* republic” OR “solomon island*” OR “somalia*” OR “south africa*” OR “south sudan*” OR “sri lanka*” OR “st. lucia*” OR “st. vincent and the grenadi*” OR “sudan*” OR “syrian arab republic” OR “syrian*” OR “tajikistan*” OR “tanzania*” OR “thailand*” OR “timor-leste*” OR “togo*” OR “tonga*” OR “tunisia*” OR “türkiye” OR “turk*” OR “turkmenistan*” OR “tuvalu*” OR “uganda*” OR “ukrain*” OR “uzbekistan*” OR “vanuatu*” OR “vietnam*” OR “west bank and gaza*” OR “yemen*” OR “zambia*” OR “zimbabwe*” | 397,893 |
|  |  | **16** | TI “afghanistan*” OR “albania*” OR “angola*” OR “armenia*” OR “azerbaijan*” OR “bangladesh*” OR “belarus*” OR “belize*” OR “benin*” OR “bhutan*” OR “bolivia*” OR “bosnia and herzegovina*” OR “botswana*” OR “bulgaria*” OR “burkina faso*” OR “burundi*” OR “cabo verde*” OR “cambodia*” OR “cameroon*” OR “central african republic*” OR “chad*” OR “chile*” OR “chin*” OR “colombia*” OR “comoros*” OR “democratic republic of congo*” OR “congo, republic*” OR “costa rica*” OR “côte divoire*” OR “croatia*” OR “cuba*” OR “curaçao*” OR “czech republic*” OR “djibouti*” OR “dominica*” OR “dominican republic*” OR “ecuador*” OR “egypt*” OR “el salvador*” OR “equatorial guinea*” OR “eritrea*” OR “estonia*” OR “eswatini*” OR “ethiopia*” OR “fiji*” OR “gambia*, the” OR “georgia*” OR “ghana*” OR “grenada*” OR “guatemala*” OR “guinea*” OR “guinea-bissa*” OR “guyan*” OR “haiti*” OR “honduras*” OR “india*” OR “indonesia*” OR “jamaica*” OR “jordan*” OR “kazakhstan*” OR “kenya*” OR “kiribati*” OR “korea*” OR “korea, democratic republic.” OR “kosovo*” OR “kyrgyz*” OR “lao*” OR “latvia*” OR “leban*” OR “lesotho*” OR “liberia*” OR “lithuania*” OR “madagascar*” OR “malawi*” OR “malaysia*” OR “maldiv*” OR “mali*” OR “marshall island*” OR “mauritania*” OR “mauriti*” OR “mexic*” OR “micronesia*” OR “micronesia, federal state” OR “moldov*” OR “mongolia*” OR “morocc*” OR “mozambiq*” OR “myanmar*” OR “namibia*” OR “nepal*” OR “nicaragua*” OR “niger*” OR “nigeria*” OR “north macedonia*” OR “mariana island*” OR “pakistan*” OR “papua new guinea*” OR “paraguay*” OR “peru*” OR “philippin*” OR “poland” OR “polish” OR “russia*” OR “rwanda*” OR “samoa*” OR “são tomé and príncipe*” OR “senegal*” OR “sierra leone*” OR “slovak* republic” OR “solomon island*” OR “somalia*” OR “south africa*” OR “south sudan*” OR “sri lanka*” OR “st. lucia*” OR “st. vincent and the grenadi*” OR “sudan*” OR “syrian arab republic” OR “syrian*” OR “tajikistan*” OR “tanzania*” OR “thailand*” OR “timor-leste*” OR “togo*” OR “tonga*” OR “tunisia*” OR “türkiye” OR “turk*” OR “turkmenistan*” OR “tuvalu*” OR “uganda*” OR “ukrain*” OR “uzbekistan*” OR “vanuatu*” OR “vietnam*” OR “west bank and gaza*” OR “yemen*” OR “zambia*” OR “zimbabwe*” | 292,240 |
|  |  | 17 | AB “afghanistan” OR “albania” OR “angola” OR “armenia” OR “azerbaijan” OR “bangladesh” OR “belarus” OR “belize” OR “benin” OR “bhutan” OR “bolivia” OR “bosnia and herzegovina” OR “botswana” OR “bulgaria” OR “burkina faso” OR “burundi” OR “cabo verde” OR “cambodia” OR “cameroon” OR “central african republic” OR “chad” OR “chile” OR “china” OR “colombia” OR “comoros” OR “democratic republic of congo” OR “congo, republic” OR “costa rica” OR “côte divoire” OR “croatia” OR “cuba” OR “curaçao” OR “czech republic” OR “djibouti” OR “dominica” OR “dominican republic” OR “ecuador” OR “egypt” OR “el salvador” OR “equatorial guinea” OR “eritrea” OR “estonia” OR “eswatini” OR “ethiopia” OR “fiji” OR “gambia, the” OR “georgia” OR “ghana” OR “grenada” OR “guatemala” OR “guinea” OR “guinea-bissau” OR “guyana” OR “haiti” OR “honduras” OR “india” OR “indonesia” OR “jamaica” OR “jordan” OR “kazakhstan” OR “kenya” OR “kiribati” OR “korea, dem. rep.” OR “kosovo” OR “kyrgyz republic” OR “lao pdr” OR “latvia” OR “lebanon” OR “lesotho” OR “liberia” OR “lithuania” OR “madagascar” OR “malawi” OR “malaysia” OR “maldives” OR “mali” OR “marshall islands” OR “mauritania” OR “mauritius” OR “mexico” OR “micronesia, fed. sts.” OR “moldova” OR “mongolia” OR “morocco” OR “mozambique” OR “myanmar” OR “namibia” OR “nepal” OR “nicaragua” OR “niger” OR “nigeria” OR “north macedonia” OR “northern mariana islands” OR “pakistan” OR “papua new guinea” OR “paraguay” OR “peru” OR “philippines” OR “poland” OR “russian federation” OR “rwanda” OR “samoa” OR “são tomé and príncipe” OR “senegal” OR “sierra leone” OR “slovak republic” OR “solomon islands” OR “somalia” OR “south africa” OR “south sudan” OR “sri lanka” OR “st. lucia” OR “st. vincent and the grenadines” OR “sudan” OR “syrian arab republic” OR “tajikistan” OR “tanzania” OR “thailand” OR “timor-leste” OR “togo” OR “tonga” OR “tunisia” OR “türkiye” OR “turkmenistan” OR “tuvalu” OR “uganda” OR “ukraine” OR “uzbekistan” OR “vanuatu” OR “vietnam” OR “west bank and gaza” OR “yemen” OR “zambia” OR “zimbabwe”: | 191,929 |
|  |  | **18** | TI “afghanistan” OR “albania” OR “angola” OR “armenia” OR “azerbaijan” OR “bangladesh” OR “belarus” OR “belize” OR “benin” OR “bhutan” OR “bolivia” OR “bosnia and herzegovina” OR “botswana” OR “bulgaria” OR “burkina faso” OR “burundi” OR “cabo verde” OR “cambodia” OR “cameroon” OR “central african republic” OR “chad” OR “chile” OR “china” OR “colombia” OR “comoros” OR “democratic republic of congo” OR “congo, republic” OR “costa rica” OR “côte divoire” OR “croatia” OR “cuba” OR “curaçao” OR “czech republic” OR “djibouti” OR “dominica” OR “dominican republic” OR “ecuador” OR “egypt” OR “el salvador” OR “equatorial guinea” OR “eritrea” OR “estonia” OR “eswatini” OR “ethiopia” OR “fiji” OR “gambia, the” OR “georgia” OR “ghana” OR “grenada” OR “guatemala” OR “guinea” OR “guinea-bissau” OR “guyana” OR “haiti” OR “honduras” OR “india” OR “indonesia” OR “jamaica” OR “jordan” OR “kazakhstan” OR “kenya” OR “kiribati” OR “korea, dem. rep.” OR “kosovo” OR “kyrgyz republic” OR “lao pdr” OR “latvia” OR “lebanon” OR “lesotho” OR “liberia” OR “lithuania” OR “madagascar” OR “malawi” OR “malaysia” OR “maldives” OR “mali” OR “marshall islands” OR “mauritania” OR “mauritius” OR “mexico” OR “micronesia, fed. sts.” OR “moldova” OR “mongolia” OR “morocco” OR “mozambique” OR “myanmar” OR “namibia” OR “nepal” OR “nicaragua” OR “niger” OR “nigeria” OR “north macedonia” OR “northern mariana islands” OR “pakistan” OR “papua new guinea” OR “paraguay” OR “peru” OR “philippines” OR “poland” OR “russian federation” OR “rwanda” OR “samoa” OR “são tomé and príncipe” OR “senegal” OR “sierra leone” OR “slovak republic” OR “solomon islands” OR “somalia” OR “south africa” OR “south sudan” OR “sri lanka” OR “st. lucia” OR “st. vincent and the grenadines” OR “sudan” OR “syrian arab republic” OR “tajikistan” OR “tanzania” OR “thailand” OR “timor-leste” OR “togo” OR “tonga” OR “tunisia” OR “türkiye” OR “turkmenistan” OR “tuvalu” OR “uganda” OR “ukraine” OR “uzbekistan” OR “vanuatu” OR “vietnam” OR “west bank and gaza” OR “yemen” OR “zambia” OR “zimbabwe”: | 155,975 |
|  |  | **19** | MH developing countries or developing nations or third world or low income countries | 19,808 |
|  |  | **20** | TI ("developing countr*" or "third world countr*" or "low income countr*" or "middle-income countr*" or "resource constrained setting*" or "Low- and middle-income countr*" or "LMIC*" or “Under-Developed Countr*” or “Under Developed Countr*” or “Less-Developed Countr*” or “Less Developed Countr*” or "Least Developed Countr*") | 9,035 |
|  |  | **21** | AB ("developing countr*" or "third world countr*" or "low income countr*" or "middle-income countr*" or "resource constrained setting*" or "Low- and middle-income countr*" or "LMIC*" or “Under-Developed Countr*” or “Under Developed Countr*” or “Less-Developed Countr*” or “Less Developed Countr*” or "Least Developed Countr*") | 29,948 |
|  |  | **22** | 15 OR 16 OR 17 OR 18 OR 19 OR 20 OR 21 | 538,369 |
|  |  | **23** | **4 AND 8 AND 14 AND 22** | **196** |

| **Database** | **Concept** |  | **Search Term** | **Count** |
| --- | --- | --- | --- | --- |
| APA PsycInfo | Accreditation | 1 | TI "accredit*" | 1,151 |
|  |  | 2 | AB "accredit*" | 8,330 |
| MA = MESH |  | 3 | MA accreditation | 580 |
|  |  | 4 | 1 or 2 or 3 | 8,613 |
|  | Health | 5 | TI "health*" | 228,111 |
|  |  | 6 | AB "health*" | 834,573 |
|  |  | 7 | MA health | 256,596 |
|  |  | 8 | 5 or 6 or 7 | 958,488 |
|  | Hospital | 9 | AB "hospital" | 113,172 |
|  |  | 10 | TI "hospital" | 21,424 |
|  |  | 11 | AB "hospitals" | 33,977 |
|  |  | 12 | TI "hospitals" | 4,440 |
|  |  | 13 | MH hospitals or hospital | 32,707 |
|  |  | 14 | MH hospitals or health care facilities | 32,707 |
|  |  | 15 | 9 or 10 or 11 or 12 or 13 or 14 | 152,920 |
|  |  | 16 | 4 AND 8 AND 15 | 420 |
|  |  | 15 | AB “afghanistan*” OR “albania*” OR “angola*” OR “armenia*” OR “azerbaijan*” OR “bangladesh*” OR “belarus*” OR “belize*” OR “benin*” OR “bhutan*” OR “bolivia*” OR “bosnia and herzegovina*” OR “botswana*” OR “bulgaria*” OR “burkina faso*” OR “burundi*” OR “cabo verde*” OR “cambodia*” OR “cameroon*” OR “central african republic*” OR “chad*” OR “chile*” OR “chin*” OR “colombia*” OR “comoros*” OR “democratic republic of congo*” OR “congo, republic*” OR “costa rica*” OR “côte divoire*” OR “croatia*” OR “cuba*” OR “curaçao*” OR “czech republic*” OR “djibouti*” OR “dominica*” OR “dominican republic*” OR “ecuador*” OR “egypt*” OR “el salvador*” OR “equatorial guinea*” OR “eritrea*” OR “estonia*” OR “eswatini*” OR “ethiopia*” OR “fiji*” OR “gambia*, the” OR “georgia*” OR “ghana*” OR “grenada*” OR “guatemala*” OR “guinea*” OR “guinea-bissa*” OR “guyan*” OR “haiti*” OR “honduras*” OR “india*” OR “indonesia*” OR “jamaica*” OR “jordan*” OR “kazakhstan*” OR “kenya*” OR “kiribati*” OR “korea*” OR “korea, democratic republic.” OR “kosovo*” OR “kyrgyz*” OR “lao*” OR “latvia*” OR “leban*” OR “lesotho*” OR “liberia*” OR “lithuania*” OR “madagascar*” OR “malawi*” OR “malaysia*” OR “maldiv*” OR “mali*” OR “marshall island*” OR “mauritania*” OR “mauriti*” OR “mexic*” OR “micronesia*” OR “micronesia, federal state” OR “moldov*” OR “mongolia*” OR “morocc*” OR “mozambiq*” OR “myanmar*” OR “namibia*” OR “nepal*” OR “nicaragua*” OR “niger*” OR “nigeria*” OR “north macedonia*” OR “mariana island*” OR “pakistan*” OR “papua new guinea*” OR “paraguay*” OR “peru*” OR “philippin*” OR “poland” OR “polish” OR “russia*” OR “rwanda*” OR “samoa*” OR “são tomé and príncipe*” OR “senegal*” OR “sierra leone*” OR “slovak* republic” OR “solomon island*” OR “somalia*” OR “south africa*” OR “south sudan*” OR “sri lanka*” OR “st. lucia*” OR “st. vincent and the grenadi*” OR “sudan*” OR “syrian arab republic” OR “syrian*” OR “tajikistan*” OR “tanzania*” OR “thailand*” OR “timor-leste*” OR “togo*” OR “tonga*” OR “tunisia*” OR “türkiye” OR “turk*” OR “turkmenistan*” OR “tuvalu*” OR “uganda*” OR “ukrain*” OR “uzbekistan*” OR “vanuatu*” OR “vietnam*” OR “west bank and gaza*” OR “yemen*” OR “zambia*” OR “zimbabwe*” | 331,779 |
|  |  | **16** | TI “afghanistan*” OR “albania*” OR “angola*” OR “armenia*” OR “azerbaijan*” OR “bangladesh*” OR “belarus*” OR “belize*” OR “benin*” OR “bhutan*” OR “bolivia*” OR “bosnia and herzegovina*” OR “botswana*” OR “bulgaria*” OR “burkina faso*” OR “burundi*” OR “cabo verde*” OR “cambodia*” OR “cameroon*” OR “central african republic*” OR “chad*” OR “chile*” OR “chin*” OR “colombia*” OR “comoros*” OR “democratic republic of congo*” OR “congo, republic*” OR “costa rica*” OR “côte divoire*” OR “croatia*” OR “cuba*” OR “curaçao*” OR “czech republic*” OR “djibouti*” OR “dominica*” OR “dominican republic*” OR “ecuador*” OR “egypt*” OR “el salvador*” OR “equatorial guinea*” OR “eritrea*” OR “estonia*” OR “eswatini*” OR “ethiopia*” OR “fiji*” OR “gambia*, the” OR “georgia*” OR “ghana*” OR “grenada*” OR “guatemala*” OR “guinea*” OR “guinea-bissa*” OR “guyan*” OR “haiti*” OR “honduras*” OR “india*” OR “indonesia*” OR “jamaica*” OR “jordan*” OR “kazakhstan*” OR “kenya*” OR “kiribati*” OR “korea*” OR “korea, democratic republic.” OR “kosovo*” OR “kyrgyz*” OR “lao*” OR “latvia*” OR “leban*” OR “lesotho*” OR “liberia*” OR “lithuania*” OR “madagascar*” OR “malawi*” OR “malaysia*” OR “maldiv*” OR “mali*” OR “marshall island*” OR “mauritania*” OR “mauriti*” OR “mexic*” OR “micronesia*” OR “micronesia, federal state” OR “moldov*” OR “mongolia*” OR “morocc*” OR “mozambiq*” OR “myanmar*” OR “namibia*” OR “nepal*” OR “nicaragua*” OR “niger*” OR “nigeria*” OR “north macedonia*” OR “mariana island*” OR “pakistan*” OR “papua new guinea*” OR “paraguay*” OR “peru*” OR “philippin*” OR “poland” OR “polish” OR “russia*” OR “rwanda*” OR “samoa*” OR “são tomé and príncipe*” OR “senegal*” OR “sierra leone*” OR “slovak* republic” OR “solomon island*” OR “somalia*” OR “south africa*” OR “south sudan*” OR “sri lanka*” OR “st. lucia*” OR “st. vincent and the grenadi*” OR “sudan*” OR “syrian arab republic” OR “syrian*” OR “tajikistan*” OR “tanzania*” OR “thailand*” OR “timor-leste*” OR “togo*” OR “tonga*” OR “tunisia*” OR “türkiye” OR “turk*” OR “turkmenistan*” OR “tuvalu*” OR “uganda*” OR “ukrain*” OR “uzbekistan*” OR “vanuatu*” OR “vietnam*” OR “west bank and gaza*” OR “yemen*” OR “zambia*” OR “zimbabwe*” | 178,058 |
|  |  | 17 | AB “afghanistan” OR “albania” OR “angola” OR “armenia” OR “azerbaijan” OR “bangladesh” OR “belarus” OR “belize” OR “benin” OR “bhutan” OR “bolivia” OR “bosnia and herzegovina” OR “botswana” OR “bulgaria” OR “burkina faso” OR “burundi” OR “cabo verde” OR “cambodia” OR “cameroon” OR “central african republic” OR “chad” OR “chile” OR “china” OR “colombia” OR “comoros” OR “democratic republic of congo” OR “congo, republic” OR “costa rica” OR “côte divoire” OR “croatia” OR “cuba” OR “curaçao” OR “czech republic” OR “djibouti” OR “dominica” OR “dominican republic” OR “ecuador” OR “egypt” OR “el salvador” OR “equatorial guinea” OR “eritrea” OR “estonia” OR “eswatini” OR “ethiopia” OR “fiji” OR “gambia, the” OR “georgia” OR “ghana” OR “grenada” OR “guatemala” OR “guinea” OR “guinea-bissau” OR “guyana” OR “haiti” OR “honduras” OR “india” OR “indonesia” OR “jamaica” OR “jordan” OR “kazakhstan” OR “kenya” OR “kiribati” OR “korea, dem. rep.” OR “kosovo” OR “kyrgyz republic” OR “lao pdr” OR “latvia” OR “lebanon” OR “lesotho” OR “liberia” OR “lithuania” OR “madagascar” OR “malawi” OR “malaysia” OR “maldives” OR “mali” OR “marshall islands” OR “mauritania” OR “mauritius” OR “mexico” OR “micronesia, fed. sts.” OR “moldova” OR “mongolia” OR “morocco” OR “mozambique” OR “myanmar” OR “namibia” OR “nepal” OR “nicaragua” OR “niger” OR “nigeria” OR “north macedonia” OR “northern mariana islands” OR “pakistan” OR “papua new guinea” OR “paraguay” OR “peru” OR “philippines” OR “poland” OR “russian federation” OR “rwanda” OR “samoa” OR “são tomé and príncipe” OR “senegal” OR “sierra leone” OR “slovak republic” OR “solomon islands” OR “somalia” OR “south africa” OR “south sudan” OR “sri lanka” OR “st. lucia” OR “st. vincent and the grenadines” OR “sudan” OR “syrian arab republic” OR “tajikistan” OR “tanzania” OR “thailand” OR “timor-leste” OR “togo” OR “tonga” OR “tunisia” OR “türkiye” OR “turkmenistan” OR “tuvalu” OR “uganda” OR “ukraine” OR “uzbekistan” OR “vanuatu” OR “vietnam” OR “west bank and gaza” OR “yemen” OR “zambia” OR “zimbabwe”: | 163,520 |
|  |  | **18** | TI “afghanistan” OR “albania” OR “angola” OR “armenia” OR “azerbaijan” OR “bangladesh” OR “belarus” OR “belize” OR “benin” OR “bhutan” OR “bolivia” OR “bosnia and herzegovina” OR “botswana” OR “bulgaria” OR “burkina faso” OR “burundi” OR “cabo verde” OR “cambodia” OR “cameroon” OR “central african republic” OR “chad” OR “chile” OR “china” OR “colombia” OR “comoros” OR “democratic republic of congo” OR “congo, republic” OR “costa rica” OR “côte divoire” OR “croatia” OR “cuba” OR “curaçao” OR “czech republic” OR “djibouti” OR “dominica” OR “dominican republic” OR “ecuador” OR “egypt” OR “el salvador” OR “equatorial guinea” OR “eritrea” OR “estonia” OR “eswatini” OR “ethiopia” OR “fiji” OR “gambia, the” OR “georgia” OR “ghana” OR “grenada” OR “guatemala” OR “guinea” OR “guinea-bissau” OR “guyana” OR “haiti” OR “honduras” OR “india” OR “indonesia” OR “jamaica” OR “jordan” OR “kazakhstan” OR “kenya” OR “kiribati” OR “korea, dem. rep.” OR “kosovo” OR “kyrgyz republic” OR “lao pdr” OR “latvia” OR “lebanon” OR “lesotho” OR “liberia” OR “lithuania” OR “madagascar” OR “malawi” OR “malaysia” OR “maldives” OR “mali” OR “marshall islands” OR “mauritania” OR “mauritius” OR “mexico” OR “micronesia, fed. sts.” OR “moldova” OR “mongolia” OR “morocco” OR “mozambique” OR “myanmar” OR “namibia” OR “nepal” OR “nicaragua” OR “niger” OR “nigeria” OR “north macedonia” OR “northern mariana islands” OR “pakistan” OR “papua new guinea” OR “paraguay” OR “peru” OR “philippines” OR “poland” OR “russian federation” OR “rwanda” OR “samoa” OR “são tomé and príncipe” OR “senegal” OR “sierra leone” OR “slovak republic” OR “solomon islands” OR “somalia” OR “south africa” OR “south sudan” OR “sri lanka” OR “st. lucia” OR “st. vincent and the grenadines” OR “sudan” OR “syrian arab republic” OR “tajikistan” OR “tanzania” OR “thailand” OR “timor-leste” OR “togo” OR “tonga” OR “tunisia” OR “türkiye” OR “turkmenistan” OR “tuvalu” OR “uganda” OR “ukraine” OR “uzbekistan” OR “vanuatu” OR “vietnam” OR “west bank and gaza” OR “yemen” OR “zambia” OR “zimbabwe”: | 81,117 |
|  |  | **19** | MA developing countries or developing nations or third world or low income countries | 4,329 |
|  |  | **20** | TI ("developing countr*" or "third world countr*" or "low income countr*" or "middle-income countr*" or "resource constrained setting*" or "Low- and middle-income countr*" or "LMIC*" or “Under-Developed Countr*” or “Under Developed Countr*” or “Less-Developed Countr*” or “Less Developed Countr*” or "Least Developed Countr*") | 3,313 |
|  |  | **21** | AB ("developing countr*" or "third world countr*" or "low income countr*" or "middle-income countr*" or "resource constrained setting*" or "Low- and middle-income countr*" or "LMIC*" or “Under-Developed Countr*” or “Under Developed Countr*” or “Less-Developed Countr*” or “Less Developed Countr*” or "Least Developed Countr*") | 15,994 |
|  |  | **22** | 15 OR 16 OR 17 OR 18 OR 19 OR 20 OR 21 | 359,900 |
|  |  | **23** | **4 AND 8 AND 15 AND 22** | **34** |

| **Database** | **Concept** |  | **Search Term** | **Count** |
| --- | --- | --- | --- | --- |
| Web of Science | Accreditation | 1 | TS=("accredit*") | 38,807 |
|  | Health | 2 | TS=("health*") | 4,415,395 |
|  | Hospital | 3 | TS=("hospital") | 1,142,426 |
|  |  | 4 | TS=("hospitals") | 270,574 |
|  |  | 5 | TS=("health care facilit*") | 8,338 |
|  |  | 6 | 3 OR 4 OR 5 | 1,307,241 |
|  |  | 7 | 1 AND 2 AND 6 | 2,583 |
| Topic:  Searches title, abstract, author keywords, and Keywords Plus. |  | 8 | TS=(“afghanistan*” OR “albania*” OR “angola*” OR “armenia*” OR “azerbaijan*” OR “bangladesh*” OR “belarus*” OR “belize*” OR “benin*” OR “bhutan*” OR “bolivia*” OR “bosnia and herzegovina*” OR “botswana*” OR “bulgaria*” OR “burkina faso*” OR “burundi*” OR “cabo verde*” OR “cambodia*” OR “cameroon*” OR “central african republic*” OR “chad*” OR “chile*” OR “chin*” OR “colombia*” OR “comoros*” OR “democratic republic of congo*” OR “congo, republic*” OR “costa rica*” OR “côte divoire*” OR “croatia*” OR “cuba*” OR “curaçao*” OR “czech republic*” OR “djibouti*” OR “dominica*” OR “dominican republic*” OR “ecuador*” OR “egypt*” OR “el salvador*” OR “equatorial guinea*” OR “eritrea*” OR “estonia*” OR “eswatini*” OR “ethiopia*” OR “fiji*” OR “gambia*, the” OR “georgia*” OR “ghana*” OR “grenada*” OR “guatemala*” OR “guinea*” OR “guinea-bissa*” OR “guyan*” OR “haiti*” OR “honduras*” OR “india*” OR “indonesia*” OR “jamaica*” OR “jordan*” OR “kazakhstan*” OR “kenya*” OR “kiribati*” OR “korea*” OR “korea, democratic republic.” OR “kosovo*” OR “kyrgyz*” OR “lao*” OR “latvia*” OR “leban*” OR “lesotho*” OR “liberia*” OR “lithuania*” OR “madagascar*” OR “malawi*” OR “malaysia*” OR “maldiv*” OR “mali*” OR “marshall island*” OR “mauritania*” OR “mauriti*” OR “mexic*” OR “micronesia*” OR “micronesia, federal state” OR “moldov*” OR “mongolia*” OR “morocc*” OR “mozambiq*” OR “myanmar*” OR “namibia*” OR “nepal*” OR “nicaragua*” OR “niger*” OR “nigeria*” OR “north macedonia*” OR “mariana island*” OR “pakistan*” OR “papua new guinea*” OR “paraguay*” OR “peru*” OR “philippin*” OR “poland” OR “polish” OR “russia*” OR “rwanda*” OR “samoa*” OR “são tomé and príncipe*” OR “senegal*” OR “sierra leone*” OR “slovak* republic” OR “solomon island*” OR “somalia*” OR “south africa*” OR “south sudan*” OR “sri lanka*” OR “st. lucia*” OR “st. vincent and the grenadi*” OR “sudan*” OR “syrian arab republic” OR “syrian*” OR “tajikistan*” OR “tanzania*” OR “thailand*” OR “timor-leste*” OR “togo*” OR “tonga*” OR “tunisia*” OR “türkiye” OR “turk*” OR “turkmenistan*” OR “tuvalu*” OR “uganda*” OR “ukrain*” OR “uzbekistan*” OR “vanuatu*” OR “vietnam*” OR “west bank and gaza*” OR “yemen*” OR “zambia*” OR “zimbabwe*”) | 5,982,181 |
| Topic:  Searches title, abstract, author keywords, and Keywords Plus. |  | 9 | TS=(“afghanistan” OR “albania” OR “angola” OR “armenia” OR “azerbaijan” OR “bangladesh” OR “belarus” OR “belize” OR “benin” OR “bhutan” OR “bolivia” OR “bosnia and herzegovina” OR “botswana” OR “bulgaria” OR “burkina faso” OR “burundi” OR “cabo verde” OR “cambodia” OR “cameroon” OR “central african republic” OR “chad” OR “chile” OR “china” OR “colombia” OR “comoros” OR “democratic republic of congo” OR “congo, republic” OR “costa rica” OR “côte divoire” OR “croatia” OR “cuba” OR “curaçao” OR “czech republic” OR “djibouti” OR “dominica” OR “dominican republic” OR “ecuador” OR “egypt” OR “el salvador” OR “equatorial guinea” OR “eritrea” OR “estonia” OR “eswatini” OR “ethiopia” OR “fiji” OR “gambia, the” OR “georgia” OR “ghana” OR “grenada” OR “guatemala” OR “guinea” OR “guinea-bissau” OR “guyana” OR “haiti” OR “honduras” OR “india” OR “indonesia” OR “jamaica” OR “jordan” OR “kazakhstan” OR “kenya” OR “kiribati” OR “korea, dem. rep.” OR “kosovo” OR “kyrgyz republic” OR “lao pdr” OR “latvia” OR “lebanon” OR “lesotho” OR “liberia” OR “lithuania” OR “madagascar” OR “malawi” OR “malaysia” OR “maldives” OR “mali” OR “marshall islands” OR “mauritania” OR “mauritius” OR “mexico” OR “micronesia, fed. sts.” OR “moldova” OR “mongolia” OR “morocco” OR “mozambique” OR “myanmar” OR “namibia” OR “nepal” OR “nicaragua” OR “niger” OR “nigeria” OR “north macedonia” OR “northern mariana islands” OR “pakistan” OR “papua new guinea” OR “paraguay” OR “peru” OR “philippines” OR “poland” OR “russian federation” OR “rwanda” OR “samoa” OR “são tomé and príncipe” OR “senegal” OR “sierra leone” OR “slovak republic” OR “solomon islands” OR “somalia” OR “south africa” OR “south sudan” OR “sri lanka” OR “st. lucia” OR “st. vincent and the grenadines” OR “sudan” OR “syrian arab republic” OR “tajikistan” OR “tanzania” OR “thailand” OR “timor-leste” OR “togo” OR “tonga” OR “tunisia” OR “türkiye” OR “turkmenistan” OR “tuvalu” OR “uganda” OR “ukraine” OR “uzbekistan” OR “vanuatu” OR “vietnam” OR “west bank and gaza” OR “yemen” OR “zambia” OR “zimbabwe”:) | 3,469,846 |
|  |  | **10** | TS=('developing countr*' OR 'third world countr*' OR 'low income countr*' OR 'middle-income countr*' OR 'resource constrained setting*' OR 'low- and middle-income countr' OR 'lmic*' OR 'under-developed countr*' OR 'under developed countr*' OR 'less-developed countr*' OR 'less developed countr*' OR 'least developed countr*') | 455,932 |
|  |  | **11** | 8 OR 9 OR 10 | 6,241,043 |
|  |  | **12** | **1 AND 2 AND 6 AND 11** | **597** |

| **Database** | **Concept** |  | **Search Term** | **Count** |
| --- | --- | --- | --- | --- |
| JBI EBP (via Ovid) | Accreditation | 1 | "accredit*".af. | 185 |
|  |  | 2 | "accredit* ".m_titl. | 3 |
|  |  | 3 | 1 or 2 | 185 |
|  | Health | 4 | "Health*".mp. [mp=text, heading word, subject area node word, title] | 7,632 |
|  |  | 5 | "health* ".m_titl. | 579 |
|  |  | 6 | 3 or 4 | 7,632 |
|  | Hospital | 7 | "hospital".mp. [mp=text, heading word, subject area node word, title] | 2,932 |
|  |  | 8 | hospital.m_titl | 158 |
|  |  | 9 | hospitals.mp. [mp=text, heading word, subject area node word, title] | 1,044 |
|  |  | 10 | hospitals.m_titl. | 22 |
|  |  | 11 | health care facilities.mp. [mp=text, heading word, subject area node word, title] | 74 |
|  |  | 12 | health care facilities.m_titl. | 1 |
|  |  | 13 | 7 or 8 or 9 or 10 or 11 or 12 | 3,132 |
|  |  | 14 | 3 AND 6 AND 13 | 145 |
|  |  | **15** | ("developing countr*" or "developing nation*" or "third world" or "low income countr*").mp. [mp=text, heading word, subject area node word, title] | 332 |
|  |  | **11** | **3 AND 6 AND 13 AND 15** | **20** |
