# Supplementary material for: Factors influencing the establishment of hospital accreditation programs in low- and middle-income countries: a scoping review
Source: Health Policy Plan. 2025 Feb 18;40(4):496–517. doi: 10.1093/heapol/czaf011 (PMC11979593; doi:10.1093/heapol/czaf011)
Supplement: czaf011_Supp [file czaf011_supp.zip › Supp/25-02-15_Scoping-Review-of-Hospital-Accreditation_V16_Supplementary_File_III.docx]

**Supplementary File III: Description of Data Extraction Items**

| **Theme and Sub-Themes** | **Description** | **Examples** |
| --- | --- | --- |
| 1. **Antecedent Influences** | Adapted from the Cambridge Dictionary, the antecedent is “something existing or happening before, especially as the cause or origin of something existing or happening later,” and influence is “the power to have an effect on people or things or a person or thing that is able to do this.” In the Antecedent Behaviour Consequence (ABC) model, antecedent “can be any event or environmental factor that occurs before a behaviour, prompting that behaviour.”  **Accordingly, Antecedent Influences were described in this theme as any event or environmental factor that occurs before the establishment and has the power to have an effect on the establishment of an accreditation program.** | Accreditation program initiations in Latin America and East Mediterranean Regions were mainly due to the regional influences of the Pan American Health Organization (PAHO) and East Mediterranean Regional Offices (EMRO) of the World Health Organization (WHO) |
| - 1. Antecedent Influences of the previous systems | **Any event or environmental factor that has the power to have an effect on the failure or the success of previously established accreditation systems was included in this theme** | Political and economic instability due to Arab Spring, as in the case of the previous Egyptian accreditation program  The need for monitoring of religious-based health facilities as in the case of the previous accreditation system in Lesotho |
| - 1. Antecedent influences of the present system | **Any event or environmental factor that has the power to have an effect on the failure or the success of the present system was included in this theme.** | USAID funding and intention to strengthen the African health systems mainly in relation to capacity development in HIV-AIDS |
|  |  |  |
| 1. **Legislation and Governance** | Legislation is described in Medical Subject Headings (MeSH) terms as “Works about the enactment of laws and ordinances and their regulation by official organs of a nation, state, or other legislative organization.” The Governance Institute of Australia mentioned that “Governance encompasses the system by which an organisation is controlled and operates, and the mechanisms by which it, and its people, are held to account.” (Governance Institute of Australia, 2023).  **Accordingly, any laws, ordinances, regulations, policies, operating and controlling systems, and accountability mechanisms related to the establishment of accreditation programs were collectively included in this theme.** | South African constitution, National Health Policy, The National Health Bill, and The White Paper for the Transformation of the Health System in South Africa provided the legislative framework for the national accreditation body. Accordingly, the Council for Health Service Accreditation of Southern Africa (COHSASA) was established as an independent, non-partisan unit. |
| - 1. Accreditation Legislation | **Any laws, ordinances, regulations, and policies related to the establishment of accreditation programs were included in this theme.** | The Lebanon accreditation system was based on the legislative decree #139 of 1983, which allowed the Ministry of Public Health to evaluate and accredit hospitals. |
| - 1. Accreditation Governance | The World Health Organization (WHO) describes the system as “a collection of interacting elements comprising all the organizations, institutions, and resources that are devoted to producing health actions” (World Health Organization, 2000). The organization structure defines “how activities such as task allocation, coordination, and supervision are directed toward the achievement of organizational aims” (Pugh, 1990). The International Society for Quality in Health Care (ISQua) described accountability as the “responsibility and requirement to answer for tasks or activities” (ISQua, 2015).  **Accordingly, the collection of interacting elements (institutions, people, resources) that operate and control the establishment of accreditation programs and accountability mechanisms (responsibility and requirement to answer for allocation, supervision, and coordination of tasks or activities) contributed to the establishment of accreditation programs were included in this theme.** | SafeCare is a collaboration between PharmAccess, JCI of the United States, and COHSASA of South Africa and is designed to adapt, establish, and implement ISQua-accredited standards in resource-constrained settings. |
| - - 1. Organizational Structure | **Organizations, Institutions, or their sub-entities that are responsible and required to answer for allocation, supervision, and coordination of tasks or activities of the accreditation program were described in this theme.** | During the Safe Care establishment, a steering committee and a technical committee were formed. |
| - - 1. Composition | **People or people groups who are responsible and required to answer for allocation, supervision, and coordination of tasks or activities of the accreditation program were described in this theme.** | During the Safe Care establishment, a steering committee was formed with high-level representatives from the National Hospital Insurance Fund (NHIF), the Health in Africa Initiative, and PharmAccess. A technical committee was formed with the quality managers and assessors from the above three organizations. |
| - - 1. Finances | **Financial resources (external and internal) utilized for responsibility and requirement to answer for allocation, supervision, and coordination of tasks or activities of the accreditation program were described in this theme.** | The SafeCare accreditation program in Kenya was established as a collaboration of the Kenyan Ministry of Health, NHIF, the Health in Africa Initiative of the International Finance Corporation, and PharmAccess to ensure Universal Health Coverage. |
| - - 1. Operating procedures | The ISQua defined the procedure as “Written sets of instructions conveying the approved and recommended steps for a particular act or series of acts..” (ISQua, 2015). ISQua identified the following processes in setting up an external evaluation organization, i.e., establishing a preliminary board or advisory committee, proposing a governance board and framework, funding of the program, and setting up strategic, operational, and financial management systems (Fortune et al., 2015).  **Therefore, Written sets of instructions conveying the approved and recommended steps, which are responsible and required to answer for allocation, supervision, and coordination of tasks or activities of the accreditation program, were described in this theme, and these were in** **relation to the setting up strategic, operational and financial management systems** (Survey management was described in theme 3.6) | During the Safe Care establishment, the functions of the steering committee were to discuss the strategic directions, prepare a MoU describing objectives, designing of operation plans, and decide activities for supervising the implementation of operational plans. The functions of the technical committee were to draft and implement the operational work plan and to report back to the steering committee. This committee was responsible for the capacity development of NHIF assessors, software implementation, planning standard-based assessments, preparation of standards for assessments, integration of assessments to the step-wise certification system, and the contracting of healthcare providers. |
|  |  |  |
| 1. **Establishment Characteristics** | All accreditation programs have four main elements. These include (1) the development of an organization in which accreditation efforts are housed. (2) The development of standards and the accompanying specific criteria; (3) The implementation of the survey process, including hiring and training surveyors and scoring and reporting the results. 4. Incentives/disincentives and institutional support (Smits et al., 2014). ISQua has also recognized that developing standards and assessment methodologies are two key elements in accreditation establishment, other than organizational aspects and evaluation systems (Fortune et al., 2015). Organizational characteristics are described in Theme 2, and evaluation systems / survey processes are described in Theme 4.  **Accordingly, the development of standards, principles of the standards, assessors, and incentives were described in this theme.** | The Colombian accreditation system is composed of Accreditation, Information, Audit, and Incentive Systems. The design was done in three stages. (1) The Planning Stage was composed of a national review, an international review, and a marketing study. The reviews identified key concepts in an accreditation system. (2) The Design Stage was composed of the designing of standards and incentive systems. It identified nine principles for the organization of the accreditation system. (3) The implementation phase is composed of the selection and training of assessors and the development of tools for assessment. One of the barriers encountered was the change of political leadership, resulting in delays in finalizing the legislative framework. |
| - 1. System of Developing the Standards | The ISQua defined standard as “a desired and achievable level of performance against which actual performance is measured” (ISQua, 2015). The WHO defined the system as “a collection of interacting elements comprising all the organizations, institutions, and resources that are devoted to producing health actions” (World Health Organization, 2000).  **Organizations, institutions, resources, and actions that contributed to the development of desired and achievable levels of performance were described in this theme**. | The initial Ugandan accreditation standards and tools were finalized during a discussion with 34 participants who formed the Ugandan standards committee. After the finalization of standards, they were pilot-tested in 40 Ugandan hospitals. |
| - - 1. Standard Committee | **Organizations, Institutions, or their sub-entities that are responsible and required to answer for the development of desired and achievable levels of performance were described in this theme.** | Participants who were familiar with the Ugandan health policy were assigned to the standard committee. |
| - - 1. Standard Committee Composition | **People or people groups who are responsible and required to answer for the development of desired and achievable levels of performance were described in this theme.** | The Ugandan standards committee included representatives from the Ministry of Health, National Bureau of Standards, district health offices, hospital directors, professional associations, the World Health Organization, university academia, private practitioners, and funding agencies. |
| - - 1. Process of developing standards (including pilot testing) | The ISQua defined the process as a “series of interrelated activities and communications which accomplish services.” (ISQua, 2015). The ISQua guidelines described 16 principles of planning, developing, and evaluating accreditation standards (Environmental scanning, relationship to other standards, planned process, evidence-based adaptation, participatory approach, range of services, clarity, clear framework, unambiguous, pilot-tested, approval, documentation, information and education, implementation, feedback, and publication) (ISQua, 2015).  **A series of interrelated activities and communications contributed to developing, planning, and evaluating standards in the above 16 areas were described in this theme.** | Ugandan standards committee participants were divided into seven small groups for each of the seven domains and expected to finalize the standards and decide on the authority to administer them.  Then, the standards were pilot-tested in 40 out of 131 Ugandan hospitals and requested to conduct the self-assessment based on the finalized standards. The study team visited the hospitals, trained hospital staff (hospital administrators, nursing officers, unit heads, and officers who conducted the self-assessment) on the tool, and conducted the survey within the hospital to confirm the accuracy and consistency of the ratings given by the hospital staff, validated the responses with the staff through a de-briefing session and answered all the questions. The process was conducted for 1 - 3 days. |
| - 1. Principles of Standards | The ISQua defined standard as “a desired and achievable level of performance against which actual performance is measured” (ISQua, 2015). ISQua described the following principles of standards, i.e., assessing and submitting institutional performance through self-assessment tools, rating scales, core criteria, and non-applicable criteria, risk assessment, and technical review (ISQua, 2015). Out of these, self-assessment and final technical review are described in Theme 4. ISQua also described the range of standards, but it was excluded as a theme, as this scoping review is only focusing on hospital standards.  **This theme described the rating scale, categorization of core and non-applicable criteria, and risk assessment.** | Iranian accreditation standards were revised in three stages. The first version's issues were more focused on structure and processes and less focused on outcomes and performance, a high number of standards, and unclear standards (2010 - 3754 standards and 8104 measurable elements). The second version issues were still having a high number of standards (2157 measurable elements). The third version had 248 standards and 903 measurable elements with modified standards and assessment methodology. |
| - - 1. Content | ISQua described core criteria as follows: central organisational processes processes safeguarding competencies, processes with immediate impact on patient safety and clinical effectiveness, and formally approved, evidence-based standards with a clear purpose. In addition, standards are classified as structural, process, and outcome standards. ISQua defines structural standards as “standards which address the relatively stable characteristics of healthcare providers, their staff, tools and resources, and physical and organizational settings.” Process Standards are “standards which address the interrelated processes of different organizational and clinical functions and activities.” Outcome Standards are “standards which address the results, consequences or outcomes of the performance and measurement of activities, systems and functions” (Fortune et al., 2015).  **Any content in the standard (“a desired and achievable level of performance) with respect to inclusion of structural, process and outcome aspects is described in this theme.** | Exploration of perspectives of a diverse group (hospital owners or administrators, professional associations, government officials, consumer organizations, and insurance and financial institutions) on the establishment of a private hospital accreditation system in India revealed that Despite non-agreements on the monitoring of hospital and professional charges, there was a consensus that physical aspects, equipment, consumer satisfaction, types and follow-up care, qualifications, and quantity of human resources should be monitored through the accreditation system. |
| - - 1. Categorization | **Any categorization of standards (“a desired and achievable level of performance) was described in this theme.** | In establishing Ugandan accreditation standards, standards were finalized following seven small group discussions for each of the seven domains, and 485 standards were finalized. The seven domains were: 1. Governance, management, and leadership, 2. Quality improvement and patient safety, 3. Physical infrastructure, 4. Human resource management 5. Clinical services, 6. Records and health management information systems, 7. Infection control and waste management. Two main categories of standards were prepared at the end of the meeting. (1) A basic checklist for minimum acceptable levels. (2) A more advanced guide. |
| - - 1. Rating Scale | ISQua has suggested rating the achievement level of performance as Excellent. Good, Fair, and Poor with a rating scale, which “should enable users to rate and measure consistently” (ISQua, 2015).  **Accordingly, any rating of the performance levels within the standards during the survey was described in this theme.** | In Zambia, a scoring form for each standard, the category of achievement of the standard (met, partially met, not met) with a brief explanation, overall score, and decision algorithm was developed. |
| - - 1. Risk Assessment | The risk assessment based on the risk matrix is determined by how likely the identified risk will actually happen or materialise (the likelihood) and the impact on the organisation if the risk does materialise or happen (the impact) (ISQua, 2015).  **Accordingly, the conduction of risk assessment during the survey was described in this theme.** | (Selected publications did not mention any risk assessment strategies.) |
| - 1. Surveyor | The ISQua defined a surveyor as an “external peer reviewer, assessor of organisational performance against agreed standards.” (ISQua, 2015) | In Kenya, surveyors for the NHIF accreditation program were NHIF quality assurance officers who were representative of 15 NHIF regions. |
| - - 1. Characteristics | ISQua mentioned the following characteristics of the surveyors. Number, skill mix, and mix of paid/employed or voluntary surveyors, required competencies, including personal attributes, professional qualifications, and experience, knowledge, and skill set relevant to the program (Fortune et al., 2015).  **The above characteristics (number, skill mix, competencies, and employed/voluntary) of the surveyors were described in this theme.** | The Kenyan NHIF assessors were selected from various disciplines, such as clinicians, nurses, laboratory technologists, and public health officers. |
| - - 1. Recruitment | ISQua indicated that the following competencies are required by a surveyor, i.e., “personal attributes, including the ability to communicate effectively and to work as a team member, professional qualifications and experience, current healthcare or social care sector knowledge and skills in the areas covered by the program.”  **Methods of recruitment, the process of recruitment, and the defined competencies of the prospective surveyors were described in the theme.** | The Kenyan NHIF assessors were selected based on their performance, motivation, and ability to steer quality improvement in their regions as champions. |
| - - 1. Induction Training | Induction training is the process of integrating people, processes, and technology that is required to optimize a new entrant to the organization to achieve outcomes. (Hendricks & Louw-Potgieter, 2012). ISQua has indicated that the following topics should be covered in the initial training program, i.e., standards interpretation, survey process, interviewing and observation skills, documentation review, specific areas, e.g., safety, and report writing techniques (Fortune et al., 2015). Any training process should include needs assessment, formulation of objectives, design, implementation, and evaluation (Blanchard, 2018).    **The needs assessment, formulation of objectives, design of the training, implementation, and evaluation of the process of integrating people, processes, and technology that is required to optimize a new surveyor to the accreditation system to achieve desired outcomes were described in this theme.** | In Jordan, a competency development system was also developed by developing a curriculum, instructor guides, and resource materials for six certification courses targeting surveyors and others. The assessor certification is composed of classroom teaching, survey observations, survey conduction under supervision, and examination. On successful completion, HCAC assessor certification for two years would be granted. |
| - - 1. Continuous Professional Development | Continuous Professional Development (CPD) is defined by the Health Care Professions Council of the United Kingdom as a range of learning activities through which health professionals maintain and develop throughout their careers to ensure that they retain their capacity to practice safely, effectively, and legally within their evolving scope of practice (Health Care Professions Council, 2012, 2017)  **A range of learning activities through which accreditation surveyors maintain and develop their competencies to ensure that they retain their capacity to practice effectively within the evolving scope of practice was described in this theme.** | Certification is granted for two years for the Jordanian HCAC assessors. Within the two years of certification, certified assessors were required to conduct two surveys, attend four continuous education sessions, and be oriented to new standards as and when they were updated. |
| - 1. Incentives | Strategies that are useful to promote and sustain accreditation programs, especially when the program is not mandatory (Fortune et al., 2015). | Multiple incentives were mentioned by the participating stakeholders in the Joint Learning Network (JLN) |
| - - 1. Financial | According to the Cambridge Dictionary, financial means “relating to money or how money is managed” (Cambridge University Press, 2023).  **Accordingly, strategies related to money or management of money, which were useful to promote and sustain accreditation programs, were described in this theme.** | JLN participants mentioned the following financial incentives, i.e., fast-track payments and greater reimbursement rates of insurance reimbursements from accredited hospitals in collaboration with insurance schemes, availability of accreditation ratings for marketing of medical tourism (Egypt, Brazil, and Mexico), micro-financing solutions for the accredited hospitals, social marketing, and branding campaign for communities to enhance the utilization of services from accredited hospitals (Kenya) |
| - - 1. Non-Financial | According to the Cambridge Dictionary, financial means “relating to money or how money is managed” (Cambridge University Press, 2023).  **Accordingly, strategies that were not related to money or management of money and which were useful to promote and sustain accreditation programs were described in this theme.** | Moroccan study participants mentioned following non-financial incentives in their accreditation program, i.e., recognized by the institution, encouraged by the management, efforts exerted towards was worth, motivated by a desire to improve practices and to learn. |
| - 1. Communication of Standards to Stakeholders | According to the Cambridge Dictionary, communication is “the various methods of sending information between people and places,” and stakeholders are “people who are involved with an organization, society, etc. and therefore have responsibilities towards it and an interest in its success” (Cambridge University Press, 2023).  **Accordingly, methods of sending information regarding the standards, once they are finalized, to the people who were involved with the accreditation, had responsibilities towards it, and had an interest in its success were described in this theme.** | In Liberia, early and regular engagement with relevant stakeholders through pre-implementation communication campaigns, meetings, sharing of standards, tools and schedules, feedback on questions on standards, and press releases enabled the support and motivation of all stakeholders for the accreditation process. |
| - 1. Management of Surveying Process | The ISQua defined the process as a “series of interrelated activities and communications which accomplish services.” (ISQua, 2015) and described four steps in the survey management process, i.e., contracting with the client organization, planning and conducting the surveys, report writing, and performance indicators (Fortune et al., 2015).  **Out of these, the interrelated activities and communications related to contracting with client organization (service agreement, training and educational support, provision of facilitators, pre-survey reviews and mock surveys, excluding self-assessment) and planning of the survey (scope and duration of the survey and composition and size of the survey team) were described in this there.**  Survey conduction, report writing, and performance monitoring were described in these four, and the operating procedures of the accreditation body were described in theme 2. | In Colombia, during the final phase, the Hospital Management Centre supported the training and transfer of knowledge to the accreditation body and institutions to conduct assessments.  In Botswana, awareness to introduce the accreditation program was done to Ministry of Health officials and facility managers by COHSASA. Subsequently, COHSASA trained health staff on conducting self-evaluations, integrating compliance data into the online monitoring system, and performing quality improvements. |
|  |  |  |
| 1. **Accreditation Program Implementation (Survey)** | The ISQua defined a survey as an “external peer review which measures the performance of the organisation against an agreed set of standards” (ISQua, 2015). | In 2019, the second set of Lebanese standards was revised by the French consultants, which gained ISQua accreditation, and surveys were conducted by four contracted private companies. |
| - 1. Pre-survey activities | The ISQua defined a survey as an “external peer review which measures the performance of the organisation against an agreed set of standards” (ISQua, 2015)  **Any activities that were conducted prior to the external peer review were described in this theme, and it included the self-assessment conducted by the health facility.** | The pre-survey process of accreditation in India is composed of implementing standards for three months, conducting an internal self-assessment, and submitting the application with the compiled self-assessment checklist. |
| - 1. Process of Survey | The ISQua defined the process as a “series of interrelated activities and communications which accomplish services.” (ISQua, 2015).  **Accordingly, a series of interrelated activities and communications to accomplish external peer review, which measured the performance of the organisation against an agreed set of standards, were described in this theme. It was extended from the commencement to the end of the on-site survey up to the submission of the survey report.**  Self-assessment and any other activities prior to the on-site assessment were described in theme 4.1, and management of the surveying process was described in theme 3.6. The level of decision-making on the award of accreditation will be described in theme 4.3. | In Zambia, trained surveyors conducted the accreditation surveys by using a review of documents, site tours, staff interviews, patient interviews, and observations, and it was expected that surveys would conduct every two to three years. However, the Zambia Health Accreditation Council (ZHAC) did not communicate the results until late 2000, which was one of the reasons for failure. |
| - 1. Characteristics of the Survey | Specific characteristics of the external peer review process, such as step-wise surveys and voluntary or mandatory periodicity, of the accreditation surveys were described in this theme. | Participating stakeholders in the Joint Learning Network (JLN) recommended a guided, step-wise implementation process in LMICs to address the capacity limitations and for motivation, as in India, where the same set of standards was used to grant three categories of accreditation awards. In Ghana and Kenya, staged implementation was used as a strategy to assess the resource-poor facilities ("SafeCare" Surveys). |
| - 1. System of Awarding Accreditation | The WHO defined the system “as a collection of interacting elements comprising all the organizations, institutions and resources that are devoted to producing health actions” (World Health Organization, 2000). The ISQua indicated that the awarding of accreditation will be based on recognition decisions, which are demonstrated by compliance with the standards. Awarding of accreditation will be based on the on-site assessment report submitted by surveyors and may be decided by a separate system (Fortune et al., 2015).  **The interacting elements of all the institutions and resources that were devoted to producing accreditation awarding decisions were described in this theme.** | Interpreting survey data and making accreditation decisions was the responsibility of the accreditation agency, Zambia Health Accreditation Council (ZHAC). It included review, discussion, and agreement of survey results based on ZHAC policies and compilation of reports to be distributed to hospitals. |
| - 1. Characteristics of the Awarding of Accreditation | ISQua mentioned that “Accreditation status is normally awarded for a period of between one and four years. Sometimes there are different grades of achievement, e.g., conditional, or with commendations, or exemplary” (Fortune et al., 2015).  **Accordingly, specific characteristics of awarding the accreditation, such as grading of achievement and periodicity or the duration of the award, were described in this theme.** | In Rwanda, three leveled system was developed (Level 1 - developed policies, procedures, and plans to critical standards with staff awareness; Level 2 - implementation of policies with risk-reduction strategies; Level 3 - Data to evidence the compliance of standards and monitoring system to evidence improving quality) |
| - 1. Surveillance and public disclosure of data from the surveys | According to the Centre for Diseases Control, USA surveillance is “the ongoing, systematic collection, analysis, and interpretation of health-related data essential to planning, implementation, and evaluation of public health practice, closely integrated with the timely dissemination of these data to those responsible for prevention and control” (Centers for Disease Control and Prevention (CDC), 2014).  **The ongoing, systematic collection, analysis, and interpretation of accreditation survey data, integrated with the timely dissemination to those who were responsible for planning, implementation, and evaluation of hospital practices, were described in this theme. The dissemination of survey data to the public was described as “public disclosure.”** | Public disclosure of the survey findings and surveillance of key performance indicators will be described.  In Uganda, as a phase II, the first national survey was conducted in 2002/2002, and only 47 out of 128 hospitals achieved accreditation status. Although the results were not made to the public, some accredited hospitals used scores for marketing. |
|  |  |  |
| 1. **Evaluation of Accreditation Program Outcomes** | The ISQua defined evaluation as “Assessment of the degree of success in meeting the goals and expected results (outcomes) of the organisation, services, programs or clients.” (ISQua, 2015)  **Accordingly, assessment of the degree of success in meeting the goals and expected results of an accreditation program were described in this theme.** | In Lebanon, only 47 out of 128 hospitals achieved accreditation status in the first national survey. In the Second national survey, 85 out of 142 surveyed hospitals achieved status of accreditation. |
| - 1. Process of outcome evaluation | The ISQua defined the process as a “series of interrelated activities and communications which accomplish services.” (ISQua, 2015).  **Accordingly, interrelated activities and communications in relation to the assessment of the degree of success in meeting the goals and expected results of an accreditation program were described in this theme.** | In Rwanda, initial situation analysis and formulation of standards (Essential Hospital Accreditation Standards Framework) was conducted by technical experts of Management Sciences for Health (MSH). |
| - 1. Results of outcome evaluation | **The results of the assessment of the degree of success in meeting the goals and expected results of an accreditation program were described in this theme** | Following the assessment in Rwanda, facilitators (Quality Improvement Officers) were appointed, and by 2014, four out of five hospitals achieved level 1 accreditation. |
|  |  |  |
| 1. **Contextual Factors during establishment** | Adapted from the Cambridge Dictionary, context is “the situation within which something exists or happens, and that can help explain it.” (Cambridge University Press, 2023). According to the implementation sciences concept, “context is the set of circumstances and characteristics or unique factors that surround a particular implementation effort” (Nilsen & Bernhardsson, 2019).  **Accordingly, circumstances, characteristics, and factors that contributed during the establishment phase of an accreditation program were included in this theme.** | In Lesotho and Swaziland, the decision to establish hospital accreditation was mainly driven by external forces, the desire to improve the quality of care, the influence of the global trend towards accreditation in the 1990s, and global actors such as WHO, ISQua, and self-inclination to mimic best practices.  In Egypt, Lebanon, and Jordan, there is a lack of physical resources, infrastructure, finances for infrastructure development and improvement activities, and shortages of human resources within the health care organizations and system issues such as lack of licensing systems, non-availability of incentives, and inadequate finances for training and certification were contributed to the failures of accreditation programs. |
| - 1. Enablers | **An enabler is a set of circumstances and characteristics or unique factors that support and facilitate the establishment of an accreditation.** | In Botswana, enablers were the supportive strategies employed by the Ministry of Health, empowerment of staff due to training, regular review of the program by COHSASA advisors, and the existence of an online data system. |
| - 1. Barriers | **A barrier is a set of circumstances and characteristics or unique factors that impede and challenge the establishment of an accreditation.** | In Botswana, the barriers experienced were a lack of sense of ownership due to externally imposed programs, perception of accreditation as a time-consuming activity, centralized procurement system preventing, prompt remedial actions for shortcomings, and loss of experiences and knowledge due to the transfer of human resources between health facilities. |

**References**

Blanchard, P. N. (2018). *Effective training: Systems, strategies, and practices*. SAGE Publications.

Cambridge University Press. (2023). *Cambridge Dictionary*. Cambridge University Press. Retrieved 11th September 2023 from <https://dictionary.cambridge.org/dictionary/english/context>

Centers for Disease Control and Prevention (CDC). (2014). *Introduction to Public Health*. U.S. Department of Health and Human Services. Retrieved 16th September 2023 from <https://www.cdc.gov/training/publichealth101/surveillance.html>

Fortune, T., O’Connor, E., & Donaldson, B. (2015). Guidance on designing healthcare external evaluation programmes including accreditation. *Dublin, Ireland: International Society for Quality in Healthcare (ISQua)*.

Governance Institute of Australia. (2023). *What is governance?* Governance Institute of Australia Retrieved 13th September 2023 from <https://www.governanceinstitute.com.au/resources/what-is-governance/>

Health Care Professions Council. (2012). *Your guide to our standards for continuing professional development*. HCPC.

Health Care Professions Council. (2017). *Continuing professional development and your registration*. HCPC.

Hendricks, K., & Louw-Potgieter, J. (2012). A theory evaluation of an induction programme. *SA journal of human resource management*, *10*(3), 1-9.

ISQua. (2015). Guidelines and Principles for the Development of Health and Social Care Standards. In (4th ed.). Dublin, Ireland: ISQua EEA.

Pugh, D. S. (1990). *Organization Theory: Selected Readings*. Penguin.

Smits, H., Supachutikul, A., & Mate, K. S. (2014). Hospital accreditation: lessons from low- and middle-income countries. *Globalization and health*, *10*, Article 65. <https://doi.org/10.1186/s12992-014-0065-9>

World Health Organization. (2000). *The world health report 2000: health systems: improving performance*. World Health Organization.
