# Supplementary material for: Factors influencing the establishment of hospital accreditation programs in low- and middle-income countries: a scoping review
Source: Health Policy Plan. 2025 Feb 18;40(4):496–517. doi: 10.1093/heapol/czaf011 (PMC11979593; doi:10.1093/heapol/czaf011)
Supplement: czaf011_Supp [file czaf011_supp.zip › Supp/25-02-15_Scoping-Review-of-Hospital-Accreditation_V16_Supplementary_File_IV.docx]

**Supplementary File IV: Summary of Exclusion Criteria during Abstract Screening**

| **Excluded** | **2312** |
| --- | --- |
| **Other** | **1134** |
| Organ Donation and Transplantation/ Organ Donation / Tissue Banking / Medical Ethics |  |
| Personalized Medicine / Bio-banking |  |
| Microbiology-related topics Communicable Diseases / Infection Control / Epidemiology |  |
| Nursing Homes / Aged Care / Hospice Care |  |
| Accreditation as Recommendations and /or Guidelines, / Accreditation tools and /or Accredited Setting used for Study Design |  |
| Private Sector |  |
| Ayurveda / Chinese Medicine/ Acupuncture/ Integrative Medicine / Complementary Medicine |  |
| Health Technology / Biomedical Department / Bio-Medical Engineering |  |
| Information Technology / Information and Communication Technology / Social Media / Digital Health |  |
| Insurance / **Universal Health Coverage**/ **Health Insurance**/ Assessment of Social Value/ Social Determinants of Health / Health Financing Mechanisms |  |
| Chaplaincy Programs / Spirituality |  |
| Supportive Care - Palliative Care / Rehabilitation / Nutrition / Pre-hospital Care / Disaster Management / Occupational Health / Physiotherapy / Speech and Language Therapy / Optometry / Baby Friendly Hospitals / Medico-legal Services |  |
| Clinical Care - Endoscopy Services / Bariatric Surgery / Neonatal Care / Intensive Care / Trauma Management / Emergency Medicine / Cardiology and Coronary Care/ Toxicology / Paediatrics / Obstetrics / COVID–19 Management / Oncology / Nephrology / Pain Management / Mental Health / Pulmonary Function Laboratory / Cardiac Catheterization Laboratory / Sleep Laboratory |  |
| Management – Policy / Health System / Human Resources / Management of Health Services / Leadership / Hospital Design / Environmental Management / Waste Management |  |
| Satisfaction – Employee / Patient / Staff Motivation |  |
| Medical Tourism / Preventing Cross-border Transfer for Medical Care (Indonesia – Malaysia) |  |
| Quality-related (Patient Safety / TQM / QI / CQI / QA / QC / Protocol Adherence / Outcomes / Audits / Readiness Assessments for Accreditation) | 593 |
| Health Professional Education - Education including Continuous Professional Development / Mentorship Programs / Training / Competency Development / Community Health Workers and Volunteer Education (ASHA - Accredited Social Health Activist Education) / Educational Strategies e.g., Curriculum Development | 505 |
| Laboratory | 344 |
| Community Settings - Community Screening / Community Interventions / Community Health workers / Community Interventions / Sporting Events / Accredited Social Health Workers / Rural Health / Preventive Health / Gender Based Violence and Safe Abortion Care in Community / Community Care / Patient-centred medical homes / Integrated Community-Based Home Care (ICHC) / Residential Care (Excluding Community Pharmacists / Pharmacy) | 183 |
| Pharmacy / Pharmacists / Medication Management/ Medication Reconciliation/ Anti-Coagulation Stewardship / Pharmaceutical Care / Clinical Pharmacists / Community Pharmacists / Community Pharmacy / Anti-microbial Stewardship / (Excluding Pharmacy Education) | 160 |
| Educational Certifications - Credentialing / Professional or Competency Certifications | 150 |
| Studies from Developed Countries | 142 |
| Licensing or Certification (Quality Related) – Practice Certifications / Disease specific care certifications | 84 |
| Public Education - Patient Education/ Health Promotion / Community Engagement / Partnership between Caregivers, Patients and Healthcare Professionals / Smoking Cessation / Health Education / Care-giver Education / Education of Food-Handlers (Educational level as a factor was NOT included) | 74 |
| Primary Healthcare / Preventive Health | 74 |
| Transfusion Medicine | 73 |
| Reviews | 46 |
| Medical Records and Record Certifications - Death Certification / Cause of Death / Civil Registration and Vital Statistics / End of Life Certification / Birth Certification / Puerperal Sterilization with Well Baby Certificate / Medical Certificates | 40 |
| Other Certifications – Disease Eradication Certifications / Architectural Certifications / IT System Certifications / Medical Device Certifications / Fitness Certifications / Medicine Certifications | 36 |
| ISO and Other Accreditation Systems / Shari'ah Compliant Hospital | 32 |
| Radiology | 26 |
| Research Related (Research Priorities, Research Ethics / Research Capacity) | 24 |
| Animal Studies | 07 |
|  |  |
