# Supplementary material for: Factors influencing the establishment of hospital accreditation programs in low- and middle-income countries: a scoping review
Source: Health Policy Plan. 2025 Feb 18;40(4):496–517. doi: 10.1093/heapol/czaf011 (PMC11979593; doi:10.1093/heapol/czaf011)
Supplement: czaf011_Supp [file czaf011_supp.zip › Supp/25-02-15_Scoping-Review-of-Hospital-Accreditation_V16_Supplementary_File_V.docx]

Supplementary File V: Summary Study Publications with Synthesis of Study Findings, Policy Implications and QuADS Score

| **Publication Title**  **Journal** | **Author**  **Year** | **Country**  **Name of the Accreditation Organization** | **Study Design**  **Aims**  **QuADS Score** | **Publication Synthesis and Policy Implications of the study findings** |
| --- | --- | --- | --- | --- |
| The accreditation program of hospital institutions in Argentina  Salud Publica de Mexico (Public Health of Mexico) | Marracino 1993 | Argentina ARG  The Mixed Commission for the Development of the Quality of Medical Care (COMCAM) | Historical Case Study  This publication describes the establishment of the Argentine accreditation system and the results of a situational analysis, which evaluates the strategies and experiences of the establishment of the Argentine accreditation system.  **QuADS Score** - 12 / 39 | The Mixed Commission for the Development of the Quality of Medical Care (COMCAM) was established with the participation of medical and non-medical professional associations; social security associations and hospital associations and with the General Cooperation Agreement of Latin American Federation of Hospitals (FLH) and Pan American Health Organization (PAHO). COMCAM developed the accreditation system in 1991. The following activities were conducted between 1991 and 1992. (1) primary data evaluation of healthcare facilities (2) publication of the accreditation manual for health facilities (3) analysis of the health facility accreditation processes (5) publication of accreditation documents (6) publication of categorization of health facilities, which was depended on risks, services availability, and related practices. The Primary data evaluation identified notable deviances from the standards. Accreditation system comprised of 38 mandatory and non-mandatory standards with three levels of increasing demand to monitor and establish the gradual achievement of standards and to educate surveyors on how to achieve the next level of demand. |
| Chapter 9 Single Health Accreditation System in Colombia: An Opportunity for the Improvement of the Sector  In Certificación y acreditación en los servicios de salud. Modelos, estrategias y logros en México y Latinoamérica (Certification and accreditation in health services. Models, strategies and achievements in Mexico and Latin America) | Orjuela de Deeb 2007 | Colombia  Colombian Institute of Technical Standards and Certification (ICONTEC) | Historical Case Study  This study evaluates the Colombian accreditation program and the development process.  A pre-implementation marketing study was conducted among 183 secondary and tertiary-level public and private health sector entities.  **QuADS Score** - 15 / 39 | The overall system comprised Accreditation, Information, Audit, and Incentive Systems. The design was done in three stages. (1) The Planning Stage comprised a national review, an international review, and a marketing study. The reviews identified key concepts in an accreditation system. The marketing study indicated that the majority was aware of quality, only a minority favoured a national accreditation body, and the private sector was willing to pay between $10,000 to $50,000 for the accreditation. (2) The Design Stage was composed of designing standards and incentive systems. It identified nine principles for the organization of the accreditation system. (3) The implementation phase was  comprised of the selection and training of assessors and the development of tools for assessment. Finally, the accreditation system was organized on the following principles: (1) based on continuous quality improvement, (2) based on prestigious incentives, (3) self-evaluation and external assessment based on standards, (4) valid for three years (5) voluntary process (6) standards more focused on processes and outcomes (7) Accreditation Body was ICONTEC, which was chosen by Ministry of Health and composed of professionals with recognized track records and knowledge on quality (8) Supportive (9) Suitable and trained assessors. The Hospital Management Centre was considered as the advisory body and the knowledge and information centre in the process of accreditation. One of the barriers encountered was the change of political leadership, resulting in delays in finalizing the legislative framework |
| Chapter 8 Evaluation and accreditation of health services in Costa Rica, 1998-2005  In Certificación y acreditación en los servicios de salud. Modelos, estrategias y logros en México y Latinoamérica (Certification and accreditation in health services. Models, strategies and achievements in Mexico and Latin America) | Núñez 2007 | Costa Rica  Not mentioned in the publication | Historical Evaluation Case Study  This study evaluates the accreditation program and establishment in Costa Rica. The results from three accreditation evaluation rounds in 24 public general hospitals and four private hospitals from 1998 to 2002 were described in the publication.  **QuADS Score** - 12 / 39 | The accreditation program was initiated with the regional influence of PAHO/WHO in 1994, due to inadequate resource allocation to health services, despite increased provision services, and increased need to achieve Universal Health Coverage. Standards were developed through Twenty-two inter-disciplinary and inter-institutional committees. By 2002, three evaluations were completed. Results revealed that there were significant gaps in human resources and infrastructure standards due to resource constraints, but there were significant improvements, mainly evidenced in smaller hospitals. The study mentioned the importance of a participatory approach with stakeholders, linkages with the social security financing system, provision of resources, consumer participation, and ensuring the balance of independence between the evaluated entities, regulators, and evaluators and balance between structural with process and outcome standards |
| A stakeholder approach towards hospital accreditation in India.  Health Policy and Planning | Nandraj et al. 2001 | India IND  Not mentioned in the publication | Mixed-Method Cross-sectional study  Exploration of the perspectives of a diverse group of stakeholders on establishment characteristics of the private hospital accreditation system in India.  **QuADS Score** - 30 / 39 | The study indicated that the accreditation system should be supportive, educative, voluntary, and non-punitive, which aims to upgrade standards and continuous quality assurance. Everyone agreed that the accreditation body should be an independent, autonomous, non-profit body, and the process should initiate with a self-assessment and then be followed up by an external assessment. The differences of opinions were elicited for the composition of the accreditation body and on monitoring of hospital and professional charges. |
| The National Accreditation Board for Hospital and Health Care Providers accreditation program in India.  World Hospitals and Health Services | Gyani et al. 2014 | India IND  National Accreditation Board for Hospital and Health Care Providers (NABH) | Historical Case Study  This case study reviews the process of accreditation establishment in India, including benefits and encountered problems.  **QuADS Score** - 3 / 39 | The study described the reasons for the low level of participation in the accreditation program and problems with the accreditation process. Only a few healthcare organizations were accredited due to being a voluntary process, perception of non-value addition, no evidence of an increase in revenue, inadequate health professionals to guide the accreditation, and no enabling mechanisms. Problems with the accreditation process were that lack of buy-in from health professionals, especially medical staff, inability to meet timelines, especially regarding documentation, training, and implementation, lack of smooth information flow of quality indicators with concerns on accuracy and quality, poor reporting of safety information and poor conduction of medical audits |
| Hospital accreditation process impact evaluation. Final Report  Published by the USAID ASSIST Project. Chevy Chase, MD: University Research Co., LLC (URC) | Broughton et al., 2018 | Indonesia IDN  "National Hospital Accreditation Committee" (KARS) | Mixed-method Retrospective / Cross-sectional Study  This publication evaluates the impact of Indonesian accreditation program implementation. In addition, it analyses the differences in the implementation of JCI vs. JCI + National Accreditation (KARS) and the effect of National Health Insurance (JKN) on patient outcomes.  **QuADS Score** - 36 / 39 | The results of the study revealed that there were general improvements and improved performance across all the ten audited domains between the baseline and end-line periods in both KARS only and JCI-KARS hospitals at a similar degree. Interviews revealed that JCI was more detailed and performed with more attention than KARS, but perceived that KARS improved gradually in consistency and rigour. In addition, the initially exerted financial and logistical burden was gradually weaned off with better coordination with the insurance system and JKN had a highly positive impact on accreditation due to its incentive effect. There was a concern about the high cost of implementing JCI standards and the cost-benefit ratio. |
| Regulating the quality of health care: Lessons from hospital accreditation in Australia and Indonesia.  The Nossal Institute for Global Health, Working Paper Series | Hort et al., 2013 | Indonesia IDN  National Hospital Accreditation Committee" (KARS) | Historical Case Study  This publication was based on an analysis of hospital accreditation systems in Australia and Indonesia, using the framework of responsive regulation by Ayres and Braithwaite (1995).  **QuADS Score** - 21 / 39 | The Indonesian accreditation program was initiated in 1995 and the accreditation agency was established in 1998 as the "National Hospital Accreditation Committee" (KARS), with the influence of the Australian standards. However, as there was poor coverage and limited engagement of relevant stakeholders, modifications were initiated with legislative reforms. In 2010, existing standards were modified, using the JCI framework as the base, and the linkages with the government health insurance system were established. This review identified barriers as (1) Increased fragmentation due to the involvement of many groups such as BPRS, KKPRS, PPRA, and PPI and non-integration of BPRS into the accreditation structure. (2) Lack of clarity on accreditation between KARS and provincial and district office due to non-representation of KARS at provincial or district levels (3) Poor access to updated resource materials and guidelines on the process of accreditation, especially for the clinicians (4) Poor engagement and support from hospital staff, especially the unit heads and doctors, resulting poor implementation of clinical indicators (5) Issuing licenses to health facilities, that were not conform with the minimal standards, mainly due to political pressure. (6) Lack of resources and incentives to make required investments (7) Lack of capacity to manage the accreditation program (8) Lack of capacity for voluntary quality improvement by clinicians (9) Lack of engagement and support from the provincial and district level governments |
| Developing a hospital accreditation model: a Delphi study  BMC Health Services Research | Mosadeghrad et al. 2021 | Iran, Islamic Rep. IRN  The Office of Healthcare Accreditation of Ministry of Health and Medical Education (MOHME) | Mixed Method Prospective Study  This publication illustrates the process of development of a national accreditation model for Iranian hospitals.  **QuADS Score** - 36 / 39 | The first development round was implemented in 2012-2013, and by 2020, four rounds of modifications were done. Despite these repeated modifications there was no integration and cohesion between components, and inadequate attention was paid to the process and outcomes. In addition, high number of structural standards, more inclination to documentation, poor surveyor competencies and survey methods were contributed for the short-term changes. During the present round, a comparative review of 20 global accreditation models, interviews with health staff, and three rounds of modified Delphi technique with experts were used. The final model had seven enabling constructs and three results constructs |
| Developing a model for accreditation of Iranian teaching hospitals: a qualitative study  Ethiopian Journal of Health Sciences | Sharifi et al. 2019 | Iran, Islamic Rep. IRN  Office for Accreditation of Healthcare Institutions in the Ministry of Health, Treatment, and Medical Education (MOHTME) | Mixed Methods Prospective Longitudinal Study  This publication illustrates the process of accreditation standards establishment of Iranian teaching hospitals and analyse the effectiveness.  **QuADS Score** - 36 / 39 | The Iranian accreditation program for teaching hospitals was developed through a four-stage process. (1) Review of eight international accreditation models and compare the Iranian program (2) Identify the weaknesses of the existing system through interviews (3) Integration of findings of 1 and 2 to develop the new model (4) validation of the proposed model using Delphi technique with experts based on Applicability, Conformity with the top-level documents, Simplicity, Acceptance by beneficiaries, Coherence and Integrity, Comprehensiveness and Suitability to the context. At the end of the process, a model with 12 dimensions and 94 standards was created. |
| Iran hospital accreditation standards: challenges and solutions  The International Journal of Health Planning and Management | Ghazanfari et al. 2021 | Iran, Islamic Rep. IRN  The office for healthcare institutions’ accreditation at Ministry of Health | Qualitative Cross-sectional study  The study analysed the perceptions of the accreditation standards development process, challenges and contents of the standards.  **QuADS Score** - 30 / 39 | Iranian accreditation standards were revised in three stages due to multiple issues in previous versions. The study identified issues of hospital accreditation standards about the content and development process. Main concerns were non-availability of an independent scientific committee, low level of knowledge among committee members on accreditation standards, ambiguity of the development process, unbalanced, ambiguous, and unmeasurable standards, Inconsistencies in the construct of the standards, inappropriate classifications, unclear scoring system, and vague demarcation between licensing and accreditation standards. It also mentioned relevant solutions to the identified problems such as multi-disciplinary and independent scientific committee. to benchmark the experiences of already existing accreditation bodies. to employ systematic, scientific, and consistent approaches for the development and revision of accreditation standards, to improve communication and coordination between accreditation and licensing departments, to ensure a balance between structure, process, and outcome standards, to develop standards with the hospital representatives, and to conduct a pilot study before implementation |
| An analysis of hospital accreditation policy in Iran.  Iranian Journal of Public Health | Yousefinezhadi et al. 2017 | Iran, Islamic Rep. IRN  The Office for the Accreditation of Healthcare Institutions (OAHI) of the Ministry of Health and Medical Education | Qualitative Retrospective Study  This qualitative research explores the Iranian accreditation policy-making process, associate factors, and the impact to generate reflections for future processes.  **QuADS Score** - 33 / 39 | The study describes the establishment of Iranian accreditation in domains of Walt and Gilson framework, i.e., Context, Content, Actors, Policy Making Process - Agenda Setting, and Policy Formulation. The initial hospital evaluation system was transformed into a hospital accreditation system in 2012. More inclination to structural aspects, public dissatisfaction, and lack of safety were the main driving forces of transformation. Accreditation standards were developed and adapted to Iranian hospitals by reviewing standards of pioneering international and regional accreditation systems and through focus group discussions with accreditation experts. Standards were revised after the first round of surveys, depending on the inputs of hospital managers and health staff. Barriers in the first round were categorized into Human Resources, Standards, Process, and Surveyors. The second round was conducted with fewer standards, increasing surveyor training, emphasizing more on the process rather than documentation, and involving two senior surveyors as lead surveyors. However, standards and scores were still ambiguous, and there was a lack of inter-surveyor reliability. |
| Challenges of implementation of hospital accreditation in Iran: an exploratory factor analysis  Journal of the Egyptian Public Health Association | Tashayoei et al. 2020 | Iran, Islamic Rep. IRN  Ministry of Health | Mixed-method Cross-Sectional Study  The research analysed the challenges of implementing the accreditation program in Iranian hospitals.  **QuADS Score** - 36 / 39 | The Iranian accreditation program was established in 2010 and implemented in two phases in 2011 and 2012. The study categorized 40 challenges into six main areas: implementation, evaluation, content, structure, psychological, and management. The most prominent challenges were the inadequate time for the implementation, a high number of standards, creating confusion among health staff, increased emphasize on documentation, granting concessions only through documentation, stress, mental fatigue, and physical fatigue for the health staff. Participants perceived that assessor and assessment-related factors were least challenging |
| Jordan Healthcare Accreditation Project Final Report: June 17, 2007–March 17, 2013  USAID Publication | Arabji 2013 | Jordan JOR  Health Care Accreditation Council (HCAC) | Evaluation Case Study  This report evaluates the development of the Jordanian Accreditation program and the role played by key stakeholders, especially the University Research Co. LLC (URC).  **QuADS Score** - 3 / 39 | The study described the development pathway of the Jordan Healthcare Accreditation Project (JHAP), which focused on establishing an accreditation agency as the Health Care Accreditation Council (HCAC), the approval of a legislative framework, development of accreditation standards, and development of competency development system. The enablers were instilling confidence among staff and changing the culture, considering each hospital was a unique entity and approached differentially based on the context, conducting mini-assessments in initial two years to establish quality management structures and to create healthy completion, Implementation of standards through participatory top-bottom and bottom-up approaches, ensuring financial sustainability through development of plans, linkages with insurance sector, creating new businesses and innovations and enhancing the credibility of the HCAC. |
| The Business Case for Quality in Health Care  Improving Health Care in Low-and Middle-Income Countries: A Case Book | Spieker 2020 | Kenya KEN  Not mentioned in the publication | Historical Case Study  This case study focuses on introducing quality standards in a private hospital in Kenya and discusses the accreditation program establishment in Kenya.  **QuADS Score** - 6 / 39 | The Kenyan accreditation program was intended mainly to achieve Universal Health Coverage and was established as a collaboration between the government, insurers (NHIF), and NGO (PharmAccess). The study described the establishment of ISQua-accredited PharmAccess standards, termed “SafeCare.”, which was designed to adapt, establish, and implement ISQua accredited standards in resource-constrained settings. Surveyors were recruited from local level health professionals based on their performance, motivation and ability to steer quality improvement in their regions as champions and trained through a comprehensive training program with a component of evaluation. The provision of credit facilities in financial difficulties was an innovative aspect. Stepwise implementation was done at the institutional level, combined with on-site technical support and training. During this training, the Quality Improvement Plans were developed by the multi-disciplinary quality team with the PharmAccess Quality Officer. Trained staff in the quality team were required to initiate their own training for their departmental staff. The study described the enablers (Financial support, technical support, Comprehensive training, Linkages with Insurance) and barriers (challenges in scaling up due to competing interest of NHIF in provision of insurance and ensuring quality) of establishment and mentioned that 84% of the Kenyan health facilities have improved between first and second assessments. |
| Hospital accreditation policy in Lebanon: it's potential for quality improvement.  Le Journal medical libanais. (The Lebanese Medical Journal) | El-Jardali 2007 | Lebanon LBN  Not mentioned in the publication | Historical Case Study  This review reflected on the process of establishing the accreditation program in Lebanon and identified the barriers, in order to enhance hospital accreditation policy formulation and implementation.  **QuADS Score** - 12 / 39 | The Lebanese accreditation program was established in four phases. Standards were composed of basic and high-order standards and were focused on structural and process elements. The numerical scoring system was used. In the First national survey conducted, only 47 out of 128 hospitals achieved accreditation status. In the Second national survey, 85 out of 142 surveyed hospitals achieved the status of accreditation. Identified barriers to accreditation were the need for (1) Changes in Organizational Culture, (2) Source of ownership and funding, (3) Reimbursement vs. Quality Improvement (4) Regulations and Incentives, and (5) Outcome oriented accreditation |
| Experiences with the expansion of hospital accreditation into the developing world  Doctoral Dissertation | Babich 2015 | Lesotho and Swaziland  National Quality Assurance units - Lesotho and Swaziland | Qualitative Cross-sectional Study  This dissertation analysed the uniquely established accreditation programs in two Southern African countries against the established social theories. The study explores the factors of establishment of accreditation programs, globally and nationally (Chapter 2), linkages between quality improvement and accreditation (Chapter 3), and contextual appropriateness of establishment of contemporary accreditation programs in LMICs (Chapter 4).  **QuADS Score** - 39 / 39 | This study explored the perceptions of study participants and scrutinized the documentary evidence and archival records. The decision to establish hospital accreditation (Dissertation Chapter 2) was mainly driven by external forces, the desire to improve the quality of care. The influence of the global trend towards accreditation in the 1990s and global actors such as WHO, ISQua, and self-inclination to mimic best practices. In Swaziland, an accreditation program was initiated with USAID-SAHCD funding with the collaboration of COHSASA in 2006. In Lesotho, the previous program was abandoned in 2007, and COHSASA standards had to be adopted by 2010 due to a lack of technical capacity and less reputation of the own system and as an answer for the resource constraints. Participants from both countries perceived they lacked real ownership of the accreditation program but only satisfied the external reviewers. In addition, they also mentioned the lack of resources (both countries), lack of support and follow-up (MCDI of Lesotho), and lack of acceptance due to an externally driven process (Swaziland). All participants from Lesotho and hospital groups of Swaziland had concerns with the complexity of COHSASA standards, but national leaders of Swaziland did not perceive this.  Chapter 3 - generated six themes on the benefits and risks of hospital accreditation. All the participants agreed that establishing accreditation programs brought attention to the quality of care and changed the processes in many dimensions. However, the participants had mixed views on providing a system for overall vision and accountability for actions and behaviours to conform with accreditation standards. Participants from Swaziland felt the inspections were too inspective and criticizing rather than supporting and educative. In addition, participants from both countries did not feel the ownership of the program. However, this sense of lack of ownership was augmented by the limited availability of human, physical, and financial resources, and the resultant perception of standards was beyond the control at the local level. The findings illustrated that the supporting efforts of accreditation distracted activities from other quality-related strategies.  Chapter 4 - The study findings revealed that 91.1% of the standards of Lesotho (previous MCDI) and only 50.9% of the standards of Swaziland (COHSASA) were considered appropriate. Despite the perception of the importance of standards, the need to have written policies, diversion of limited time from patient care, non-inclusion of all the staff in implementation (Swaziland), inclusion of standards for non-available services, non-conformance of standards with national policies and lack of support from their national Quality Assurance Units, (This was claimed due to lack of transportation and human resources) were concerns. Most of the participants questioned the feasibility of the standards due to the lack of infrastructure, supportive services, and financial, physical, and human resources. Participants highlighted the need for discussions with more inclusive groups, pre-testing to align with the national context, the need for prior awareness and training on concepts and skills on accreditation, the need for gradual implementation processes, and the commitment of the leadership in mobilizing resources and providing implementation support. |
| Introducing Health Facility Accreditation in Liberia  Global Public Health | Cleveland et al. 2011 | Liberia LBR  Not mentioned in the publication | Historical Case Study  This report describes the reflections on the process and outcomes of establishing the accreditation program during the first year in Liberia.  **QuADS Score** - 9 / 39 | The Liberian accreditation program was initiated with the collaboration of Clinton HIV/AIDS Initiative (CHAI) to uplift the fragmented health system, by implementing the standardized services of Basic Package of Health Services (BPHS), introduced by the National Health Policy and National Health Plan (2007-2011). The study indicated that the financial and political support of the government, especially during the initial stages of accreditation, branding of the accreditation system and promoting public awareness, Involvement of clinicians and managers as peer-review assessors, early and regular engagement with relevant stakeholders, and the use of online assessment and data upload tool were enablers. However, unrealistic scheduling due to transport and logistics issues, non-awareness of health facilities due to issues in communication, and lack of information on the location of health facilities at the central level created challenges |
| Chapter 7: Accreditation of Medical Units for the System of Social Protection in Health in Mexico  In Certificación y acreditación en los servicios de salud. Modelos, estrategias y logros en México y Latinoamérica (Certification and accreditation in health services. Models, strategies and achievements in Mexico and Latin America) | Ruelas et al. 2007 | Mexico  the General Directorate for Quality and Health Education (Spanish - DGCES) | Historical Evaluation Case Study  This study evaluates the Mexican accreditation program and its’ establishment, which included 2,612 evaluation visits from January 2004 to September 2006  **QuADS Score** - 12 / 39 | The study described that the accreditation system was established as a part of a social security system to provide 249 healthcare interventions, known as the Universal Catalogue of Health Services – CAUSES. The model of accreditation was to ensure quality (minimum standards required for efficiency, technical capacity, and optimum treatment in care processes, for continuous quality improvement, and for ensuring adherence to main programs of health services), Capacity (minimum standards structure, competencies, equipment for correct intervention) and Safety (minimum standards for physical well-being and technical elements for safe practice). The evaluation was done in three modalities: (1) capacity to manage 249 interventions in CAUSES, (2) ability to avoid catastrophic expenses in 6 selected diseases, (3) capacity of networking and connectivity between units through referrals and coordination between curative and preventive services in chronic disease pathways. Evaluation results revealed that, by 2006, out of 240 hospitals 153 were fully accredited, and 73 were conditionally accredited. There were notable regional disparities in the rates of accredited hospitals. One of the notable features of the program was the highly satisfactory cost/benefit ratio of 400 dollars per evaluated visit. Another important feature was the high level of utilization of accreditation performance data. |
| Results of certification audit in Mexican hospitals, a review from 2009 to 2012.  Salud pública de México (Public Health of Mexico) | Galvan-Garcia et al. 2018 | Mexico MEX  The National System of Certification of Health Care Facilities (SiNaCEAM). | Quantitative Cross-sectional Study  This study analysed the effects of accreditation interventions in 136 Mexican hospitals which applied for accreditation between 2009 - 2012 by using the evaluation card with the approved JCI standards. In Mexico, certification was considered as similar to accreditation.  **QuADS Score** - 30 / 39 | The study mentioned that, after the initially suspended system due to variations in the assessments and incorrect practices, greater emphasis was given to the training of the assessors. As per the need to have international standards with more patient safety approach and the need to promote medical tourism, more conformity with the JCI standards was ensured. In addition, linkages with the insurance payments and government contracts were established. In 2010, accreditation became obligatory for the hospitals to collaborate with the Insurance. The results indicated that 88% of the hospitals obtained a passing score. Results of 2011 were significantly better than 2009 and 2010, indicating the positive impact of accreditation. Incentives, medical tourism, activities to induce participation and sharing experiences of accredited hospitals were the enablers.) |
| Organizational impact of preparation for healthcare institution accreditation in Morocco  Sante Publique (Public Health) | Mohssine et al. 2015 | Morocco MAR  Not mentioned in the publication | Mixed-method Case Study  The objective was to identify the organizational changes due to the integration of a hospital accreditation program in a healthcare institution in Morocco, which was used as one of the four pilot settings in the Moroccan accreditation program establishment.  **QuADS Score** - 30 / 39 | The publication described perceptions about six main elements: Vision - accreditation was a change agent, the main intention was quality improvement, and staff favoured participation and anticipated effects on practice. Skills - management was able to mobilize the staff with appropriate strategies, and all the departments were involved. (However, there were negative and opposite perceptions in FGDs, and there was a gradual decline in motivation due to repeated changes in the direction). Incentives - recognized by the institution, encouraged by the management, efforts exerting towards was worth, motivated by a desire to improve practices and to learn. (However, 53.3% of respondents perceived that incentives were not up to their expectations). Resources - Participants were inclined to search for resources from sponsors (The majority of the participants believed that resources were inadequate, and only 48.1% believed that they had autonomy). Action Plan - management had adequate action plan and communication (However, FGD participants mentioned that the communication and dissemination to the self-assessment teams was inadequate due to a centralized approach, inadequate communication, and changes in the direction and pace of the process). Transformation - evaluating the patient care services, use of performance indicators, changes to services, acting as a change agent, acquiring more resources, better use of resources, networking, and creating a positive climate. (However, only 46.4% believed patients appreciated the services offered). In addition, the document review mentioned major transformations in documentation. However, transformations did not incline to address transformations in infrastructure, transformations requiring large budgets and administrative procedures. |
| Hospital accreditation: lessons from low and middle-income countries.  Globalization and Health | Smits et al. 2014 | Multiple Countries - LMICs Included  Multiple Countries - LMICs Included | Qualitative Cross-Sectional Study  The review explored the innovations employed by the participating developing countries in terms of organizational structure, standards, implementation, incentives and monitoring, and applicability of these modifications to developed countries (reverse innovation).  **QuADS Score** - 12 / 39 | The publication described that structures of the accreditation programs in developing countries were either fully or partially government-owned or owned by major insurers. It highlighted that the development of standards should be inclined to include quality improvement and gradual upgrading to higher levels with revisions, the implementation process to be guided, and stepwise, a system of rapid reporting of results, and the use of multiple incentives, were important. It mentioned that the way forward was having a free, web-based repository of all evidence and ideas about accreditation and ensuring the availability of standards from established accreditation programs at minimal or no cost through WHO or ISQua |
| Quality assurance programs through the accreditation of hospitals in Latin America and the Caribbean  Salud pública de México (Public health of Mexico) | de Moraes Novaes 1993 | Multiple Countries – LMICs Included  Multiple Countries – LMICs Included | Historical Case Study  This reflective case study describes the challenges of developing the accreditation model by the Pan American Health Organization (PAHO), World Health Organization, and Latin American Federation of Hospitals.  **QuADS Score** - 6 / 39 | An accreditation manual that comprised standards for all service levels was developed which can be adapted for the different Latin American and Caribbean Region contexts by the individual national committees and mentioned that each healthcare facility must develop its own policies and operating procedures, with the participation of its own health professionals, aligned with the standards.  The study highlighted the importance of multi-stakeholder participation in the accreditation development process and the importance of reputed and experienced surveyors. It described the challenges of government dependence, i.e., political pressure vs. provision of resources and authority. It also described the challenges of developing standards for the individual service levels and the need of accrediting the whole hospital system, due to integrated services, interrelated structures and processes. The publication recommended establishing communication strategies, providing strong incentive mechanisms, and enhancing the competencies of health staff. |
| International approaches for implementing accreditation programs in different healthcare facilities: a comparative case study in Australia, Botswana, Denmark, and Jordan  International Journal for Quality in Health Care | van Vliet et al. 2023 | Multiple Countries - LMICs Included (Botswana, Jordan)  (Botswana) The Ministry of Health and Wellness of Botswana; COHSASA (The Council for Health Services Accreditation of Southern Africa).  (Jordan) Health Care Accreditation Council (HCAC) | Qualitative Cross-sectional Study / Case Study  This publication used accreditation program document reviews and discussions with heads of accreditation bodies to comparatively analyse accreditation programs in four countries (Australia, Denmark, Botswana, and Jordan) and used a standard framework for the comparison (Goals - Why, Implementation - how Outcomes - What and lessons - Enablers and Barriers)  **QuADS Score** - 33 / 39 | (Botswana) Accreditation program in Botswana was implemented in 2009 by the Ministry of Health and Wellness of Botswana, with the collaboration of COHSASA. Following the initial comprehensive training programs for higher-level officers, facility managers and health staff, a two-staged pilot program was initiated. The barriers experienced were a lack of sense of ownership due to externally imposed programs, perception of accreditation as a time-consuming activity, centralized procurement system preventing, prompt remedial actions for shortcomings, and loss of experiences and knowledge due to the transfer of human resources between health facilities. Enablers were the supportive strategies employed by the Ministry of Health, empowerment of staff due to training, regular review of the program by COHSASA advisors, and the existence of an online data system.  (Jordan) The accreditation program in Jordan was initiated in 2004 with the collaboration of USAID and was a unique initiation as there was no initial government involvement. Later, an independent Health Care Accreditation Council (HCAC) was created in 2007. Enablers were participatory approach with engagement of all the relevant stakeholders, use of training programs, self-assessment templates and quality improvement strategies, buy-in from leaders, who use accreditation reports for managing health facilities, trained and well-prepared frontline staff. Barriers were initial suspicion and perception of staff on inability to meet accreditation standards, resistance to changes, lack of incentives or disincentives, inadequate resources and finances, insufficient training and awareness for the staff, and increased workload. |
| SafeCare: an innovative approach for improving quality through standards, benchmarking, and improvement in low-and middle-income countries.  The Joint Commission Journal on Quality and Patient Safety | Johnson et al. 2016 | Multiple Countries - LMICs Included (Ghana, Kenya, Nigeria, Namibia, Tanzania, and Zambia)  SafeCare Foundation | Evaluation Case Study  This publication describes the establishment and implementation of "SafeCare" Standards, through public-private partnerships.  **QuADS Score** - 6 / 39 | The study described the pathway of establishing SafeCare, which was implemented by three organizations, such as the SafeCare Foundation. ISQua accredited SafeCare Standards were intended to achieve stepwise improvements by focusing on important aspects of quality, safety, and risks in resource-constrained settings where fully pledged accreditation programs could not adequately be implemented. There were five levels of achievement combined with the quality strength levels. The program was combined with ISQua accredited surveyor training. Improvements were incentivized with pay-for-performance incentives, discounted rates for the loans and supply contracts, enhanced access to credit, and improved linkages with health insurance programs and public partnerships (private sector–supported health financing model). High cost of the program, need of utilize high qualified staff for at least two days, and non-apparent positive health outcomes in short-term and in level one or two implementations, were considered as challenges. |
| National accreditation programs for hospitals in the Eastern Mediterranean Region: Case studies from Egypt, Jordan, and Lebanon  The International Journal of Health Planning and Management | Mansour et al. 2021 | Multiple Countries - LMICs Included Egypt, Jordan, and Lebanon  (Egypt) – Initial establishment The Egyptian Accreditation Board (2003); Second implementation - "General Authority for Healthcare Accreditation and Regulation" (GAHAR) (2018).  (Lebanon) No government accreditation body.  (Jordan) Health Care Accreditation Council (HCAC); Overlooked by the High Health Council. | Qualitative Cross-sectional Study  This qualitative study explores the characteristics (issues, influencing factors, and lessons for LMICs) of establishing accreditation programs in Jordan, Egypt, and Lebanon, using the policy transfer framework of Dolowitz and Marsh  **QuADS Score** - 39 / 39 | (Egypt) After the initial accreditation program was terminated in 2010 due to the Arab Spring, an independent accreditation body was established in 2018. The standards were reactivated to gain ISQua re-accreditation in 2017. Barriers were political and economic instability, rapid turnover of ministers of health with subsequent changes in policies, rapid turnover of staff, poor maintenance, and lack of finances for quality improvement, continuous education, and training. Political support and ownership of the Ministry of Health, commitment of national leaders and the supportive role of multiple donor agencies, adaptation of international standards to local contexts, stage-wise implementation, non-financial incentives, staff capacity development activities, and introduction of post-graduate and certificate courses in accreditation were also enablers. The review highlighted the need for continuous support by international donor agencies by creating networks, pooling resources and expertise, and regular interactions and incremental stage-wise implementation of accreditation programs in resource-poor settings  (Lebanon) The initial accreditation was terminated due to political and economic instability following the Arab Spring and due to resource constraints following the end of funding. In 2019, the second set of standards was revised by the French consultants, which gained ISQua accreditation, and surveys were conducted by four contracted private companies. In addition, for Lebanon, common barriers for all three countries, documentation, and lack of availability of electronic medical records were perceived as barriers. Linkage of the accreditation system to regulation, reimbursement, and capacity development were the perceived enablers.  (Jordan) The accreditation program in Jordan was initiated in 2007 as the Jordan Healthcare Accreditation Project (JHAP). 2007, the project was handed over to University Research Corporation (URC). URC engaged in initial capacity-building activities, establishing an independent accreditation body, the Health Care Accreditation Council (HCAC), and standards development. Common barriers between the three countries were less pronounced in Jordan due to continued funding by USAID, where funding was also used for hospital infrastructure development and refurbishment.  The commitment of the King, legal backup for the accreditation program, financial and non-financial incentives, intention to become a hub for international medical tourism, continued USAID funding, technical expertise from JCI during initial standards adaptation and formulation of four training programs related to accreditation with the collaboration of universities were the enablers.)  During the standards development, all three countries adopted international standards, especially from JCI, to local contexts through a series of national and sub-regional meetings and revised to formulate a basic set of standards. In all three countries, there was a lack of physical resources, infrastructure, finances for infrastructure development and improvement activities and shortages of human resources within the health care organizations and system issues such as lack of licensing systems, non-availability of incentives, and inadequate finances for training and certification were contributed to the failures of accreditation programs. |
| The emergence of hospital accreditation programs in East Africa: Lessons from Uganda, Kenya, and Tanzania  Global Journal of Medicine and Public Health | Lane et al. 2014 | Multiple Countries - LMICs Included Uganda, Kenya, and Tanzania  (Uganda) Ugandan Ministry of Health.  (Kenya) National Hospital Insurance Fund (NHIF).  (Tanzania) Tanzanian NHIF | Qualitative Cross-Sectional Study  The study used a qualitative review of accreditation systems in Uganda, Kenya, and Tanzania to reflect on the lessons for the sustainable implementation of accreditation programs in LMICs.  **QuADS Score** - 30 / 39 | Ugandan Accreditation system was termed the "Yellow Star" Program and was USAID funded. The weaknesses noted in the Ugandan system were lack of national ownership of the program, paper-based recording of assessment data, and financial and human resource constraints. The participants indicated that the accreditation body should be independent of Ministry of Health and Insurance systems. The Tanzanian accreditation system had almost similar characteristics as the Kenyan system. Both systems funded by the premiums of National Hospital Insurance Fund (NHIF) and employed NHIF surveyors. Kenyan insurance reimbursement rates would depend on the accreditation assessment scores, which was a strong incentive. However, participants were ambiguous about the linkage between reimbursement rates and assessment scores. Tanzanian participants highlighted the need for an independent accreditation body, which was outside the insurance system, despite linkages made accreditation programs more sustainable. |
| Creating a pathway for public hospital accreditation in Rwanda: progress, challenges and lessons learned.  International Journal for Quality in Health Care | Binagwaho et al. 2020 | Rwanda RWA  Not mentioned in the publication | Retrospective Mixed Methods Study  This publication evaluates the experiences of the establishment and the progress of the national accreditation program in Rwanda.  **QuADS Score** - 6 / 39 | The study indicated that the number of level-1 accredited hospitals in Rwanda has increased from four in 2014 to 24 in 2018. The key successful factors were the stepwise implementation of the program combined with the training of assessors and linking the accreditation with Performance Based Financing. Identified barriers were financial, resource, and infrastructure constraints, turnover of leadership and staff, non-prioritization of accreditation, as it was a newly introduced concept, and perception of accreditation as additional work by the health staff.) |
| Status of a health care quality review program in South Africa.  International Journal for Quality in Health Care | Whittaker et al. 2000 | South Africa  ZAF  Council for Health Service Accreditation of Southern Africa (COHSASA) | Historical Case Study  This paper reviews the development of an accreditation program for South African health facilities by The Council for Health Service Accreditation of Southern Africa (COHSASA)  **QuADS Score** - 6 / 39 | The study described the three phases of the development of standards as the Normative Phase, Empirical Phase, and Accommodation Phase. The standards were developed by reviewing international standards and with the participation of multiple stakeholders. The program initiated with the influence of the systems in United Kingdom and expanded from a university setting to national level, independent, not-for-profit establishment - the Council for Health Service Accreditation of Southern Africa (COHSASA). It also described the legislative framework (constitution, policy, National Health Bill and the White Paper). There were no formal incentives in the system, but there was enhanced public image and recognition and data for evidence-based policy and planning decisions. South African health staff believed that the accreditation program contributed to significant improvements in management functions, communication between service areas, and conformance with standards and 193 facilities had entered the program. |
| Comparison of health care professionals and surveyors’ opinions on problems and obstacles in implementing quality management system in Thailand: a national survey  International Journal for Quality in Health Care | Pongpirul et al 2006 | Thailand THA  Not mentioned in the publication | Quantitative Cross-sectional study  This research explores the perceptions of healthcare professionals and hospital accreditation surveyors in Thailand on problems in accreditation standards and obstacles in implementing Quality Management systems.  **QuADS Score** - 30 / 39 | The study revealed that the main obstacles were integrating and utilizing information and staff adequacy. However, surveyors were more inclined towards processes-related obstacles, while healthcare professionals were more inclined towards resources-related obstacles. Both groups mentioned integration and utilization of information and staff adequacy. |
| Developing Hospital Accreditation Standards in Uganda  The International Journal of Health Planning and Management | Galukande et al. 2016 | Uganda UGA  Not mentioned in the publication | Qualitative Cross-Sectional Study  This publication described the process of establishing and using self-assessment accreditation tools in Uganda following a failed attempt with the USAID funding ("fizzling out" phenomenon)  **QuADS Score** - 27 / 39 | This establishment was following a failed attempt due to termination of USAID funding, resources constraints and non-alignment with the country-specific context. During current establishment, after the initial development of the tool with multi-stakeholder participation, 485 standards were finalized in two main categories, i.e., (1) A basic checklist for minimum acceptable levels. (2) A more advanced guide. Then, the standards were tested in 40 Ugandan hospitals. During the pilot testing, the study team visited the hospitals, introduced and trained the hospital staff on the tool who conducted the self-assessment, conducted the survey within the hospital to confirm the accuracy and consistency of the ratings given by the hospital staff, validated the responses with the staff through a de-briefing session and answered all the questions. |
| Problems of regulating the quality of medical care in Ukraine and the main directions of their solution  Медичні перспективи (Medical Perspective) | Tolstanov et al. 2022 | Ukraine UKR  Accreditation Commission of the Ministry of Health | Historical Case Study  This case study explores the globally available tools for improving quality and existing systems for further regulating and improving healthcare quality in Ukraine.  **QuADS Score** - 12 / 39 | This case study reviewed existing publications, recommendations from experts, and legal documents. The review of the existing system revealed drawbacks such as more inclination to administrative aspects, non-optimum process of accreditation mechanisms, poor quality of the standards, and lack of incentives. The review identified that preparation and certification surveyors and introducing accreditation methodology were challenges for developing a new system. The study also described expectations during the establishment of future accreditation programs, i.e., independent accreditation body with multi-stakeholder participation, standards inclusive of patient safety, training of surveyors, use of electronic registers and increase payments for services |
| Implementing a national hospital accreditation program: the Zambian experience.  International Journal for Quality in Health Care | Bukonda et al. 2002 | Zambia ZMB  Zambia Health Accreditation Council (ZHAC) | Retrospective Qualitative Study  This study was conducted to reflect on the Zambian hospital accreditation program, development, implementation, and challenges in the implementation of 10 milestones between 1997 - 2000.  **QuADS Score** - 18 / 39 | The study described ten milestones in accreditation program development and barriers and enablers in the domains of Mission and Philosophy, Infrastructure and Authority, Management of field operations, published standards and accreditation decision-making, Accreditation database, and Accreditation program sustainability (Donohue and O’Leary’s framework). USAID funding, integrated approach with multiple stakeholders was used to develop the accreditation program. establishment of accreditation structures (CBoH and ZHAC) with defined policies, recruitment of experienced surveyors, use of education surveys to raise awareness and use of software to manage accreditation surveys were positive factors. Staff was more in favour of accreditation over supervision, as supervision was fault-finding and was not facilitative. However, the key concerns were resource availability (human resources and financial), legal recognition and continued funding for the accreditation structures and functions, surveyor attrition, timely feedback to hospitals, ongoing training, and technical assistance |
